# DNA Checkpoint and Repair Factors Are Nuclear Sensors for Intracellular Organelle Stresses—Inflammations and Cancers Can Have High Genomic Risks

**DOI:** 10.3389/fphys.2018.00516

**Published:** 2018-05-11

**Authors:** Huihong Zeng, Gayani K. Nanayakkara, Ying Shao, Hangfei Fu, Yu Sun, Ramon Cueto, William Y. Yang, Qian Yang, Haitao Sheng, Na Wu, Luqiao Wang, Wuping Yang, Hongping Chen, Lijian Shao, Jianxin Sun, Xuebin Qin, Joon Y. Park, Konstantinos Drosatos, Eric T. Choi, Qingxian Zhu, Hong Wang, Xiaofeng Yang

**Affiliations:** ^1^Department of Histology and Embryology, Basic Medical School, Nanchang University, Nanchang, China; ^2^Center for Metabolic Disease Research, Lewis Katz School of Medicine at Temple University, Philadelphia, PA, United States; ^3^Cardiovascular Research Center, Lewis Katz School of Medicine at Temple University, Philadelphia, PA, United States; ^4^Department of Ultrasound, Xijing Hospital, Shaanxi, China; ^5^Department of Emergency Medicine, Shengjing Hospital, Liaoning, China; ^6^Department of Endocrinology, Shengjing Hospital, Liaoning, China; ^7^Department of Cardiovascular Medicine, The First Affiliated Hospital of Kunming Medical University, Yunnan, China; ^8^Jiangxi Provincial Key Laboratory of Preventive Medicine, Nanchang University, Nanchang, China; ^9^Department of Medicine, Sidney Kimmel Medical College, Thomas Jefferson University, Philadelphia, PA, United States; ^10^Department of Neuroscience, Lewis Katz School of Medicine at Temple University, Philadelphia, PA, United States; ^11^Center for Translational Medicine, Lewis Katz School of Medicine at Temple University, Philadelphia, PA, United States; ^12^Departments of Pharmacology, and Surgery, Lewis Katz School of Medicine at Temple University, Philadelphia, PA, United States

**Keywords:** DNA damage checkpoint and repair factors, inflammation, cancers, genomic instability, danger associated molecular patterns (DAMPs)

## Abstract

Under inflammatory conditions, inflammatory cells release reactive oxygen species (ROS) and reactive nitrogen species (RNS) which cause DNA damage. If not appropriately repaired, DNA damage leads to gene mutations and genomic instability. DNA damage checkpoint factors (DDCF) and DNA damage repair factors (DDRF) play a vital role in maintaining genomic integrity. However, how DDCFs and DDRFs are modulated under physiological and pathological conditions are not fully known. We took an experimental database analysis to determine the expression of 26 DNA DD*C*Fs and 42 DNA DD*R*Fs in 21 human and 20 mouse tissues in physiological/pathological conditions. We made the following significant findings: (1) Few DDCFs and DDRFs are ubiquitously expressed in tissues while many are differentially regulated.; (2) the expression of DDCFs and DDRFs are modulated not only in cancers but also in sterile inflammatory disorders and metabolic diseases; (3) tissue methylation status, pro-inflammatory cytokines, hypoxia regulating factors and tissue angiogenic potential can determine the expression of DDCFs and DDRFs; (4) intracellular organelles can transmit the stress signals to the nucleus, which may modulate the cell death by regulating the DDCF and DDRF expression. Our results shows that sterile inflammatory disorders and cancers increase genomic instability, therefore can be classified as pathologies with a high genomic risk. We also propose a new concept that as parts of cellular sensor cross-talking network, DNA checkpoint and repair factors serve as nuclear sensors for intracellular organelle stresses. Further, this work would lead to identification of novel therapeutic targets and new biomarkers for diagnosis and prognosis of metabolic diseases, inflammation, tissue damage and cancers.

## Introduction

Chronic inflammation is induced by damage associated molecular patterns (DAMPs) derived from endogenous metabolites. Further, infectious agents-, and physiochemical factors-derived pathogen-associated molecular patterns (PAMPs) generated during tissue injury or microbial invasion promote inflammation via responsive innate immune system (Yang et al., 2008). Generally, chronic inflammation contributes to approximately 25% of human cancers (Kawanishi et al., 2017). Epidemiologic studies implicated that the risk for development of cancer is increased in patients with inflammatory cardiovascular disease (van Kruijsdijk et al., 2013; Alameddine et al., 2017; Hasin et al., 2017).

Under inflammatory conditions, inflammatory cells release reactive oxygen species (ROS) and reactive nitrogen species (RNS) which cause DNA damage. If not appropriately repaired, DNA damage leads to gene mutations and genomic instability. One of the early events in the DNA damage response (DDR) is the recruitment of poly (ADP-ribose) polymerase 1 (PARP1). In addition, DNA damage checkpoint factors (DDCFs) play a crucial role by arresting the cell cycle to allow the time to repair once a damage to DNA has taken place. The detailed characterization of DD*C*Fs has classified as many as 26 human proteins into four categories including DNA damage sensors, mediators, transducers and effectors (Blanpain et al., 2011). Additionally, DNA damage repair machinery is composed of 42 DNA damage repair factors (DD*R*Fs). DDRFs are grouped into eight subgroups based on their DNA repairing mechanisms such as base excision repair, nucleotide excision repair, homologous recombination repair, non-homologous end joining, microhomology-mediated end-joining, mismatch repair, and shared mechanism subgroups (Blanpain et al., 2011; Iyama and Wilson, 2013). However, how chronic inflammatory disorders such as metabolic cardiovascular diseases and cancers modulate the expression of these DDCFs and DDRFs remains poorly defined.

In spite of recent significant progress in this front, there are a few aspects of DDCFs and DDRFs that are not explored. The questions regarding how DDCFs and DDRFs expressed in tissue and whether altered homeostasis modulate the expression pattern are not known (Lu et al., 2004, 2017). Furthermore, whether the expression of DDCFs and DDRFs is modulated in sterile inflammatory disorders and metabolic diseases is not clear (Archacki and Wang, 2004). Most of the chronic inflammatory disorders induce the production of ROS and intracellular organelle stresses. Organelle stresses such as ER (endoplasmic reticulum) stress, and mitochondrial stress induce cell death signaling pathways (Rathore et al., 2015). We recently found that caspase-1/inflammasome pathway plays a significant role in sensing metabolic danger signal associated molecular patterns (DAMPs) in hyperlipidemia (Yin et al., 2009, 2015; Shen et al., 2010; Lopez-Pastrana et al., 2015; Li Y. F. et al., 2016, 2017; Wang L. et al., 2016), hyperhomocysteinemia (Xi et al., 2016) and chronic kidney disease (Ferrer et al., 2016) and initiate inflammation/inflammatory cell death (pyroptosis). We also found that pro-atherogenic endogenous metabolites lysophospholipids such as lysophosphatidylcholines (lysoPC) act as conditional DAMPs. We previously proposed that lysoPC bind to their intrinsic receptors (conditional DAMP receptors) (Wang X. et al., 2016; Shao et al., 2017; Sun et al., 2018), and induce mitochondrial reactive oxygen species (mitoROS) (Li et al., 2013, 2016, 2017a; Cheng et al., 2017) to upregulate aortic endothelial cell activation genes via histone 3 lysine 14 acetylation-AP1-dependent pathway (Li et al., 2018). However, it is not clear whether DDCFs and DDRFs act as the nuclear sensor for intracellular organelle stress, a part of our newly proposed cellular sensor cross-talking network. We postulated that the expression of DDCFs and DDRFs is modulated in response to intracellular organelle stresses and ultimately determine the genomic stability and cell death.

To address these questions, we took a “panoramic view” at the tissue expression patterns of 26 DDCFs and 42 DDRFs. Our results demonstrated that (1) 15 out of 21 human tissues express most of DDCFs and 14 human tissues express most of DDRFs; (2) four DDCFs such as PARP1, XRCC6, XRCC5, PRKDC, and six DDRFs including APEX1, XPC, ERCC3, ERCC5, HMGB1, and MLH1 are ubiquitously expressed. We also found that selective DDCFs and DDRFs are upregulated in cells from patients with coronary artery disease, rheumatoid arthritis, Hutchinson syndrome and various cancers. Furthermore, our analysis showed that intracellular organelle stresses can modulate the gene expression of DDCFs and DDRFs. Therefore, our findings suggest that via signaling pathways that are yet to be determined, intracellular organelle stresses are conveyed to the nucleus; and in response to these stresses, DDCFs, and DDRFs may play a significant role in determining the cell fate. Our findings provide novel insights on DDCFs and DDRFs as new therapeutic targets in metabolic diseases and inflammations.

## Materials and methods

### Tissue expression profiles of DDCF and DDRF genes

**Figure 1:**
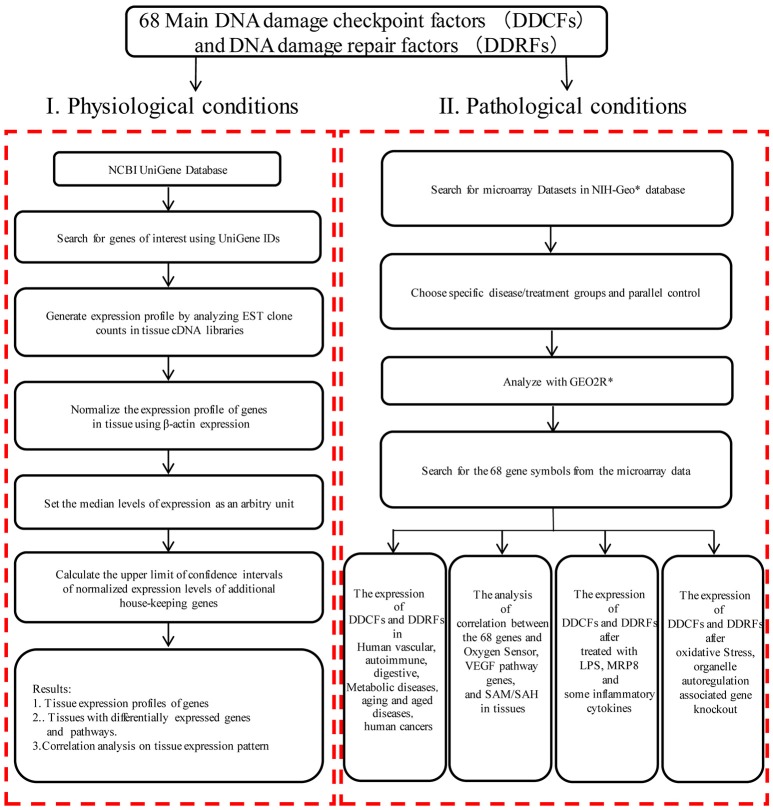
Flow chart of database mining strategy and two parts of data organization. **(I)** Database mining strategy was used to generate tissue expression profiles of genes in physiological condition. DDCFs, DNA damage checkpoint factors; DDRFs, DNA damage repair factors; NCBI, National Center of Biotechnology Information; IDs, gene identifications; EST, Expressed sequence tag; VEGF, Vascular endothelial growth factor; SAM, S-adenosylmethionine; SAH:S-adenosylhomocysteine. **(II)** DNA damage checkpoint and repair factors expression changes were analyzed with experimental data from the microarray datasets in diseases, after treated with LPS, MRP8, some inflammatory cytokines, and after oxidative stress, organelle autoregulation associated gene knockout. ^*^NIH-Geo website: www.ncbi.nlm.nih.gov/geo/; GEO2R website: www.ncbi.nlm.nih.gov/geo/geo2r/.

An experimental data mining strategy (Figure 1) was used to analyze the expression profiles of mRNA transcripts of 26 DDCF and 42 DDRF genes in 21 different human and 20 mouse tissues including heart and vasculature as we previously described (Xu et al., 2018).

### Expression profiles of DDCFs and DDRFs in disease models and cell activity

Microarray datasets were collected from the Array Express of European Bioinformatics Institute, which stores data from high-throughput functional genomics experiments. These data include the information of the expression of DDCFs and DDRFs through experiments submitted directly to Array Express or imported from the NCBI Gene Expression Omnibus database (Figure 1). The numbers of GEO datasets that were used are as follows: GSE55235, GSE81622, GSE57376, GSE27335, GSE16879, GSE27411, GSE41751, GSE1297, GSE32614, GSE13205, GSE3253, GSE34378, GSE77955, GSE79973, GSE62452, GSE45670, GSE45001, GSE70951, GSE46602, GSE36668, GSE3218, GSE75037, GSE71963, GSE9327, GSE49598, GSE11322, GSE75150, GSE39621, GSE56102, GSE67227, GSE40207, GSE60413, GSE67676, GSE13512, GSE57691, GSE9874, GSE23561, GSE19339, GSE30528, GSE25724, GSE23561, GSE55100, GSE6088, GSE43760, GSE60436, GSE46262, GSE15575, GSE9490, GSE13139, GSE36287, GSE68942, GSE56681, GSE6257, GSE37624, GSE39629, GSE8969, GSE35124, GSE6623, GSE8726, GSE92530, GSE52550. The dataset numbers that were used for each analysis is shown in tables.

### Tissue SAH and SAM measurements in mice

The concentration of S-adenosyl methionine (SAM) and S-adenosyl homocysteine (SAH) were measured in six tissues (heart, liver, lung, kidney, spleen, and brain) as we described elsewhere (Wang et al., 2017).

## Results

### Most of the DNA damage checkpoint factors and DNA damage repair factors are differentially expressed in human and mouse tissues

#### DDCF expression profile in human tissues

Twenty-six DDCFs have been characterized and classified in to three subgroups named DNA damage sensors, DNA damage mediators and transducers (Blanpain et al., 2011). However, whether the different stimulatory/suppressive environments and different types and numbers of cells present in a tissue determine the expression levels of three DDCF subgroups is not known. Therefore, we hypothesized that the differences in stimulatory/suppressive environments in tissues may differentially modulate the expression of DDCFs in human tissues.

**Figure 2:**
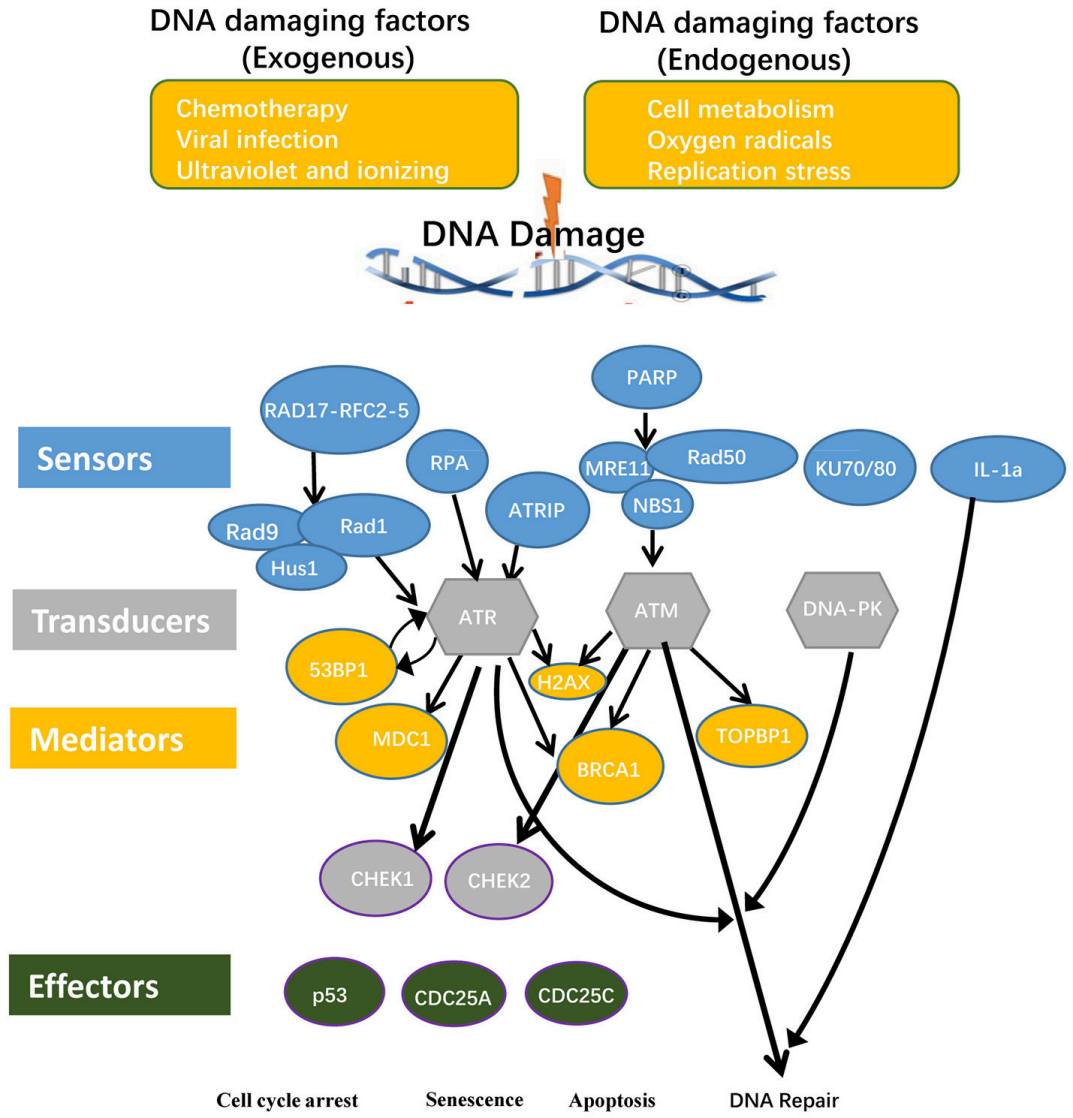
DNA damage checkpoints and the components involved in their signal transduction pathways are classified in to conceptual categories named sensors, mediators, transducers, and effectors. Consequently, DNA damage may lead to cell cycle arrest, senescence, and apoptosis. RAD17, RAD17 checkpoint clamp loader component; RFC, Replication factor C; RAD9, chromatin-binding protein; RAD1, RAD1 checkpoint DNA exonuclease; HUS1, Hus1 checkpoint homolog; RPA, replication protein A; ATRIP, ATR interacting protein; PARP1, Poly(rC)-binding protein 1; PARP2, Poly(rC)-binding protein 2; MRE11, meiotic recombination 11 homolog A; RAD50, RAD50 homolog; NBS1, Nibrin; Ku70, X-ray repair cross complementing 6 (XRCC6); Ku80, X-ray repair cross complementing 5 (XRCC5); 53BP, P53 binding protein 1; MDC1, mediator of DNA damage checkpoint protein 1; H2AX, H2A histone family, member X; BRCA1, Breast cancer 1; TopBP1, topoisomerase II binding protein 1; ATM, Ataxia telangiectasia mutated; ATR, Ataxia telangiectasia and Rad3 related; DNA-PK, DNA-activated, catalytic polypeptide; CHEK1, checkpoint kinase 1; CHEK2, checkpoint kinase 2; CDC25A, cell division cycle 25A; CDC25C, cell division cycle 25C.

To examine this hypothesis we conducted an extensive literature survey on most recently published articles. We selected 16 DNA damage sensors, 5 DNA damage mediators and 5 DNA damage transducers (Supplementary Table 1 and Figure 2) for our analysis. We then examined the expression patterns of all the 26 DDCFs in 21 human tissues by searching DNA sequencing-based data of mRNA levels at physiological conditions. Based on the level of DDCFs expression amongst human tissues examined, we classified the tissues into following three groups: if the expression of the gene is higher than the threshold, the gene was categorized in to highly expressed (++) group, if below, then the gene was considered to be low expressed gene (+). The genes that are not expressed is shown as () (Supplementary Table 2). The method of calculation the threshold is shown in Supplemental Figure 1 and was explained previously (Wang X. et al., 2016; Wang et al., 2017; Xu et al., 2018).

Out of the 26 DDCFs analyzed, four factors including three DNA damage sensor factors PARP1 (sensor), XRCC6 (sensor), XRCC5 (sensor) and one DNA damage transducer PRKDC are ubiquitously expressed in 21 human tissues in normal physiological conditions. This emphasize that these 4 genes have important house-keeping functions in sensing and monitoring DNA damage in tissues. The rest of the DDCFs are differentially expressed in human tissues. Interestingly, testis and intestine expressed all the DDCFs that we analyzed in this study. The adipose tissue expressed the least number of DDCFs. In contrast to the rest of the tissues, muscles, lymph nodes and testis had the most DDCF categorized in to highly expressed (++) group (73.1, 23.1, and 19.2% respectively). Therefore, it can be suggested that these tissues may require larger contribution from cell cycle checkpoints and house-keeping checkpoints to maintain genome integrity.

#### DDCF expression profile in mouse tissues

In addition, we also examined the mouse homologs of DDCFs (Supplementary Table 3). Unlike for human tissues, we could not identify any DDCFs that are ubiquitously expressed. However, Parp1 and Rpa1 were expressed in most of the mouse tissues included in this study. Interestingly, we could identify only Parp1 expressed in the adrenal gland. Bone marrow, brain, embryonic tissue and pancreas expressed a larger variety of DDCFs compared to the rest of the tissues analyzed. In contrast to the human tissues, most of the DDCF fell in to highly expressed (++) category in mouse tissue.

These results suggest the consistent expression of DDCFs in most human and mouse tissues, implying their physiological functions in sensing and guarding the cells against DNA damages. Our results on high expression of DDCFs in muscle are correlated well with the report demonstrating that long living tissues, such as muscle, are composed of terminally differentiated cells that irreversibly withdraw the cell cycle, and do not have the opportunity to cyclically monitor the integrity of their genome, by means of cell cycle checkpoints as dividing cells do (Latella and Puri, 2015).

#### DDRF expression in human tissues

Many pathological DNA breaks are induced by ionizing radiation, reactive oxygen species, DNA replication errors, inadvertent cleavage by nuclear enzymes, as well as physiological (regulated) breaks occurring during V(D)J recombination and immunoglobulin heavy chain class switch recombination, require end processing by nucleases and DNA polymerases to repair the DNA (Chang et al., 2017). Therefore, DDRFs play an important role in maintaining genomic integrity in cells. Herein, we hypothesized that similarly to DDCFs tissue expression profile, DDRFs may adapt a tissue expression pattern with a few house-keeping DDRFs and some inducible DDRFs.

**Figure 3:**
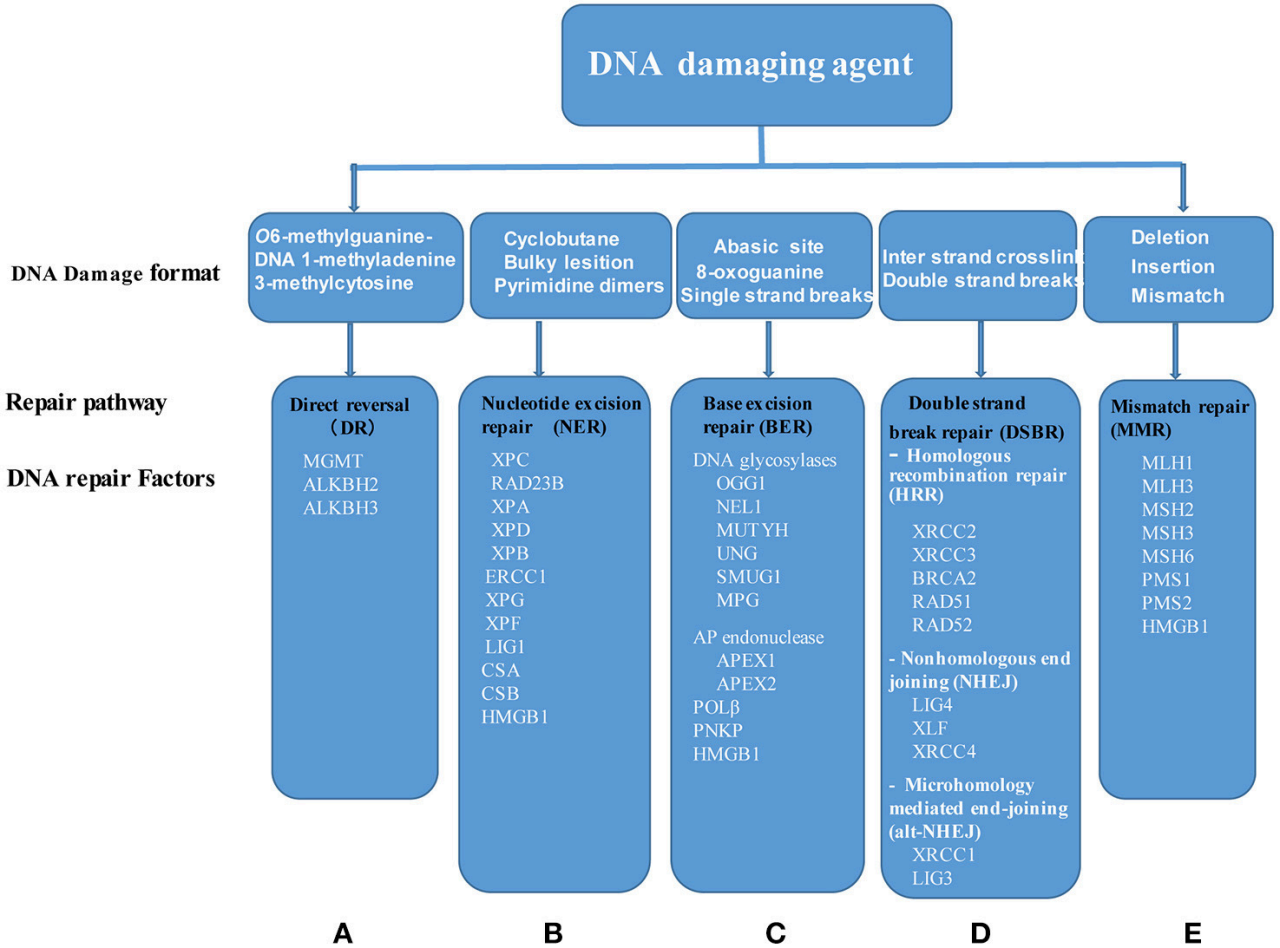
Five DNA damage repair systems have been characterized including: **(A)** DR, Direct reversal; **(B)** NER, Nucleotide excision repair; **(C)** BER, Base excision repair; **(D)** DSBR, Double strand break repair, and **(E)** MMR, mismatch repair. Of note, DSBR is divided into three major pathways: Homologous recombination repair, nonhomologous end joining and microhomology mediated end-joining. Each of DNA change repair systems are triggered by specific DNA damage agents. Of note, HMGB1 is involved in three pathways, NER, BER, and MMR. MGMT, O6-methylguanine-DNA methyltransferase; ALKBH2, AlkB, alkylation repair homolog 2; ALKBH3, AlkB, alkylation repair homolog 3; XPC, Xeroderma pigmentosum, complementation group C; RAD23B, RAD23 homolog B; XPA, Xeroderma pigmentosum, complementation group A; XPD, Excision repair cross-complementing rodent repair deficiency, complementation group 2; XPB, Excision repair cross-complementing rodent repair deficiency, complementation group 3; ERCC1, Excision repair cross-complementing rodent repair deficiency, complementation group 1; XPG, Excision repair cross-complementing rodent repair deficiency, complementation group 5; XPF, Excision repair cross-complementing rodent repair deficiency, complementation group 4; LIG1, Ligase I, DNA, ATP-dependent; CSA, Excision repair cross-complementing rodent repair deficiency, complementation group 8; CSB, Excision repair cross-complementing rodent repair deficiency, complementation group 6; HMGB1, High mobility group box 1; OGG1, 8-oxoguanine DNA glycosylase; NEIL1, Nei endonuclease VIII-like 1; MUTYH, mutY homolog; UNG, Uracil-DNA glycosylase; SMUG1, Single-strand-selective monofunctional uracil-DNA glycosylase 1; MPG, N-methylpurine-DNA glycosylase; APEX1, APEX nuclease (multifunctional DNA repair enzyme) 1; APEX2, APEX nuclease (apurinic/apyrimidinic endonuclease) 2; POLβ, Polymerase (DNA directed), beta; PNKP, Polynucleotide kinase 3′-phosphatase; XRCC2, X-ray repair complementing defective repair in Chinese hamster cells 2; XRCC3, X-ray repair complementing defective repair in Chinese hamster cells 3; BRCA2, Breast cancer 2; RAD52, RAD52 homolog; RAD51, RAD51 homolog; LIG4, Ligase IV, DNA, ATP-dependent; XLF, Nonhomologous end-joining factor 1; XRCC4, X-ray repair complementing defective repair in Chinese hamster cells 4; XRCC1, X-ray repair complementing defective repair in Chinese hamster cells 1; LIG3, Ligase III, DNA, ATP-dependent; MLH1, MutL homolog 1; MLH3, MutL homolog 3; MSH2, MutS homolog 2; MSH3, MutS homolog 3; MSH6, MutS homolog 6; PMS1, Postmeiotic segregation increased 1; PMS2, Postmeiotic segregation increased 2.

To examine this hypothesis, we collected 42 DDRFs classified into five subgroups as shown in Supplementary Table 4 and Figure 3 (Iyama and Wilson, 2013). We then categorized the 42 DDRFs in to highly expressed (++), low expressed (+) and not expressed () as we did for DDCFs (Supplementary Table 5). We found that six DDRFs including APEX1 (BER subgroup), XPC (NER), ERCC3 (NER), ERCC5 (NER), HMGB1 (MMR), and MLH1 (MMR) are ubiquitously expressed in all of 21 human tissues examined in physiological conditions. This emphasize that these 6 DDRFs have important house-keeping functions in DNA damage repair in tissues. The rest of the DDRFs are differentially expressed in human tissues. Similar to the trend seen for DDCFs expression, muscles and testis (except lymph node) had larger variety of DDRFs classified in to highly expressed (++) group. Adipose tissue and the adrenal gland recorded the least variety of DDRF expression.

#### Construction of a tissue pyramid model based on the expression level of DDCFs and DDRFs in human tissues

**Figure 4:**
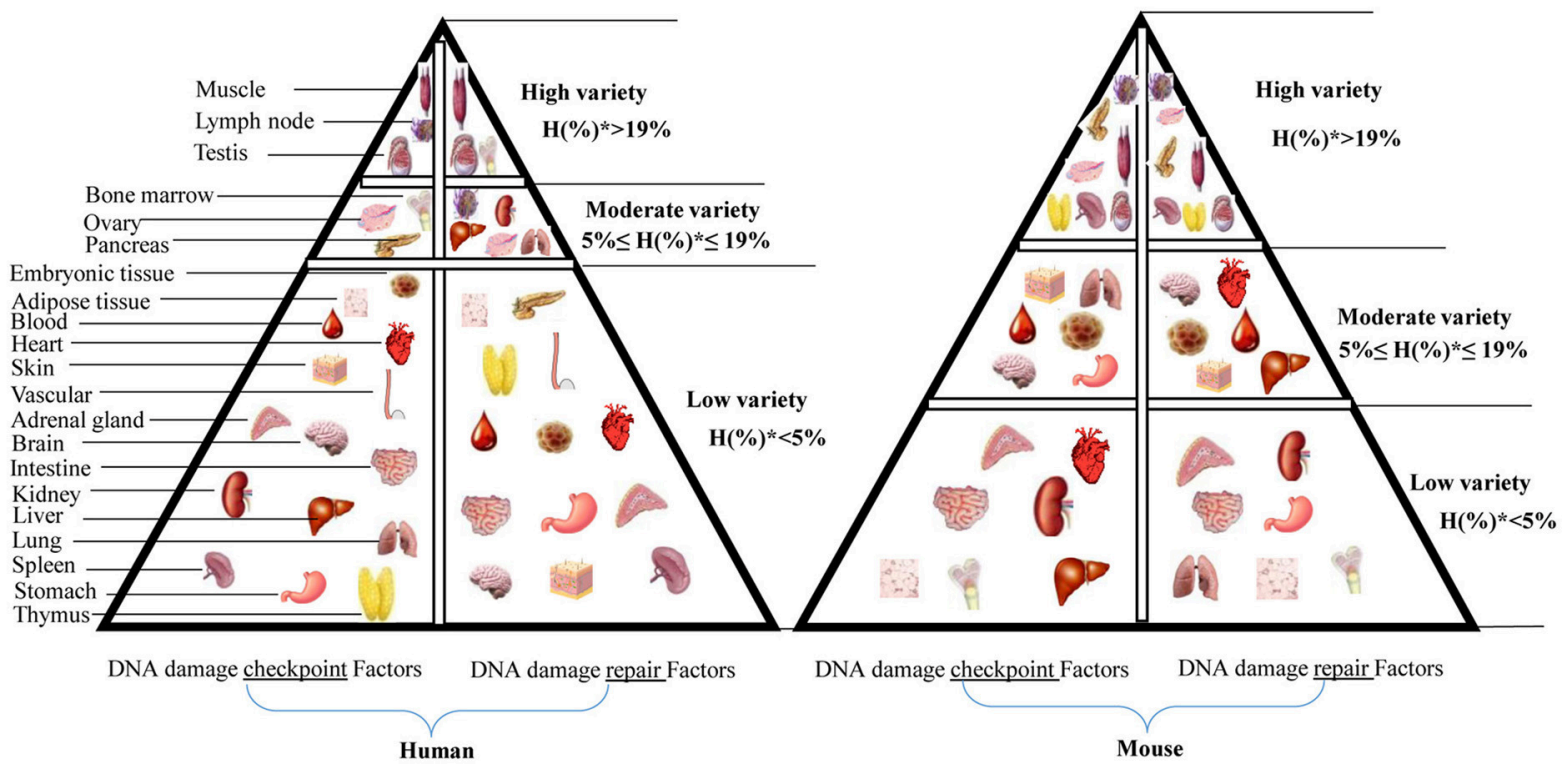
Newly proposed “DNA damage checkpoint and repair factors” model in humans and mice. This tissue pyramid model suggests that DNA damage response factors are differentially regulated in tissues. Further, abundance of DDCFs co-relate with the level of expression of DDRFs. H(%)* = number of genes with high expression in each tissue/ number of DDCFS or DDRFS.

Based on the number of DDCFs and DDRFs classified in to highly expressed (++) group, we further categorized the tissues in to three groups: high variety (tissues that had H%^*^ > 19 in Supplementary Tables 2, 3, 5, 6), moderate variety (5% ≤ H%^*^ ≤ 19% in Supplementary Tables 2, 3, 5, 6) and low variety (H%^*^ < 5% in Supplementary Tables 2, 3, 5, 6). We depicted these tissue categories for DDCFs and DDRFs in a tissue pyramid model as shown in Figure 4. Two separate tissue pyramid models were constructed for mouse and human tissues. Interestingly, muscle and testis is included in the first tier for both DDCFs and DDRFs in human tissue pyramid. Furthermore, the tissues that expressed most of DDCFs are correlated well with the tissues expressed most of DDRFs, implying that they are functionally connected in maintaining genomic integrity in tissues. Compared to human tissues, mice have higher numbers of tissues classified in top tier and middle tier, which may result from the differences of humans and mice in their living environments.

### Human vascular, autoimmune, digestive, aging diseases tend to upregulate DDCFs and DDRFs than downregulation, whereas metabolic diseases show equal modulation of DNA damage factors and repair factors

It has been reported that DNA damages and DNA strand breaks not only occur in physiological conditions but also happen in pathological conditions such as carcinogenesis/tumorigenesis (Chang et al., 2017) as well as in chronic inflammation (Kawanishi et al., 2017; Spanou et al., 2017). We hypothesized that various disease conditions, including cardiovascular diseases, autoimmune diseases, digestive diseases, aging diseases, metabolic diseases and cancers affect genomic integrity by modulating the expression of DDCFs and DDRFs. To examine this hypothesis, we performed extensive database mining and screening in the NIH-NCBI GEO DataSets database^1^ with the microarray experiments from various disease settings including vascular, autoimmune, digestive, aging, cancers/tumors from five systems, and metabolic diseases.

**Figure 5:**
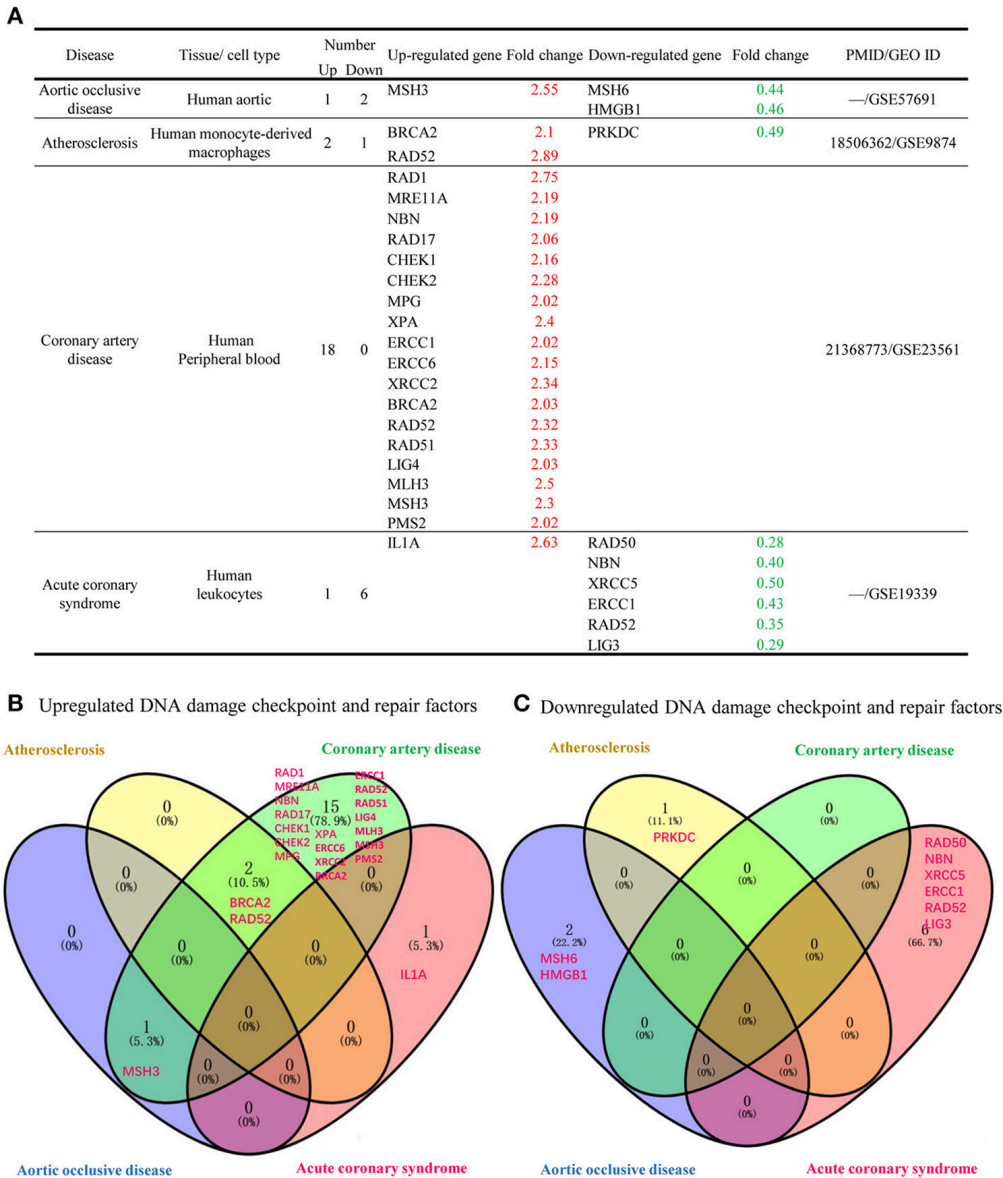
Venn Diagram shows that significantly changed DNA damage checkpoint and repair factor in tissues among 4 different human vascular diseases are mostly disease-specific. **(A)** DNA damage response factors has the tendency to be more upregulated that downregulated in 4 human vascular diseases included in this study. **(B)** Venn diagram shows that the DNA damage checkpoint and factors that are upregulated during vascular diseases are involved in distinct signaling pathways. **(C)** Downregulated DNA damage checkpoint and repair factors are involved in distinct signaling pathways in different vascular diseases.

We included 4 vascular diseases in to our study: aortic occlusive disease, atherosclerosis, coronary artery disease and acute coronary syndrome and analyzed the expression changes of DDCFs and DDRFs compared to healthy controls (Figure 5, Supplementary Tables 7, 8). The results showed that in the presence of vascular diseases, the tendency to upregulate the expression of DDCFs and DDRFs in blood cells increase. Furthermore, most of DDCFs and DDRFs tend to be upregulated in the blood cells in coronary artery disease (Figure 5B). In contrast, most of the downregulation of DDCFs and DDRFs expression in the blood cells was observed in acute coronary syndrome. The results showed that most of the vascular disease have the tendency to upregulate DDCFs and DDRFs. Furthermore, Venn analysis results showed that significantly changed DDCFs and DDRFs among four vascular diseases are mostly tissue-specific.

Interestingly, we saw a similar tendency in different autoimmune disorders we analyzed in this study. Our study included 5 autoimmune disorders such as rheumatoid arthritirs, osteoarthritis, systemic lupus erythematosus, lesional skin and asthma. Out of the 26 DDCFs analyzed, seven DDCFs were upregulated and we could not observe attenuation of any of the DDCFs. We saw an increase in expression of 3 DDRFs including Rad51 (Rheumatoid arthritis), XRCC4 (lesional skin), and BRCA2 (asthma) while 1DDRFs was downregulated (Supplementary Tables 9, 10). Similar to autoimmune disorders, the digestive disorders that we included in this study showed an upregulation of 5 DDCFs while we did not observe any downregulation of DDCFs. We studied 5 different digestive pathologies and CHEK1 was upregulated in four digestive disorders. Further, DDRFs also showed the tendency of being upregulated than being downregulated. DDRF RAD52 was only downregulated in ulcerative colitis (Supplementary Tables 11, 12).

**Figure 6:**
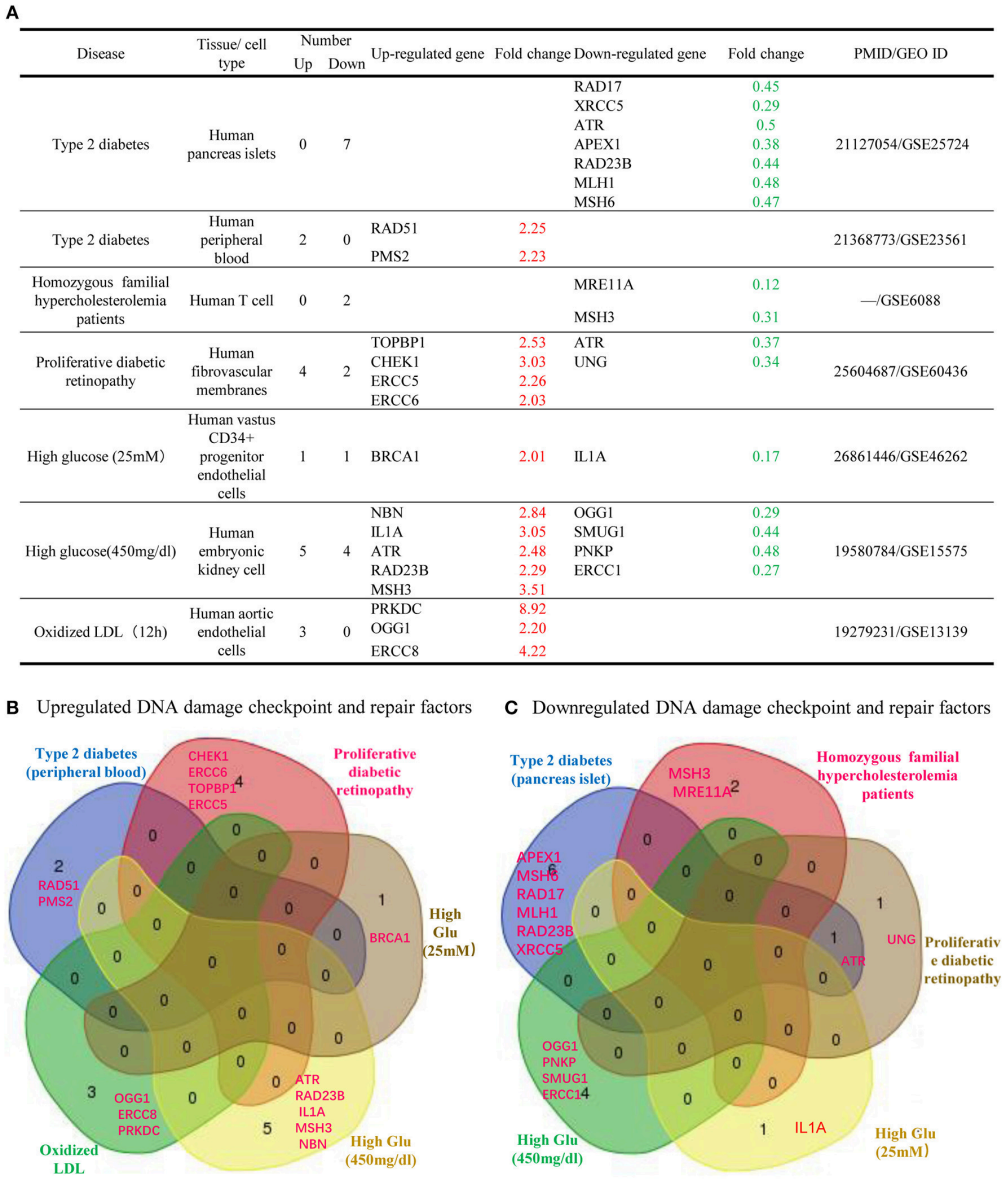
Venn Diagram shows that significantly changed DNA damage checkpoint and repair factor among metabolic diseases and in cells treated with high glucose and oxidized LDL treatment are mostly disease-specific. **(A)** Almost equal number of DNA damage response factors are induced and attenuated in metabolic diseases. Therefore, a distinct pattern of gene modulation cannot be discerned. **(B)** Venn diagram of the upregulated DNA damage response factors in different metabolic diseases. **(C)** Venn diagram of the downregulated DNA damage response factors in different metabolic diseases.

Furthermore, we analyzed whether DDCFs and DDRFs were modulated in metabolic disorders. For this analysis, we extracted microarray datasets that were conducted on Type 2 diabetes, familial hypercholesterolemia, diabetic retinopathy and also on cells that were treated with hyperglycemic and pro-atherogenic stimuli (Supplementary Tables 13, 14). The summary of our findings is shown in Figure 6. In type 2 diabetes, seven DDCFs and DDRFs were downregulated while 2 factors were upregulated in peripheral blood (Figure 6A). This indicates that the modulation of DDCFs and DDRFs by type 2 diabetes may be tissue specific. In addition, the Venn analysis revealed that modulation of DDCFs and DDRFs are disease specific (Figure 6B). However, the expression changes of DDCFs and DDRFs may be attributed to disease type; and tissue-specific responses to metabolic changes were noticed.

Moreover, we studied the effect of aging and aging disease (progeria) on the expression of DDCFs and DDRFs (Supplementary Tables 15, 16). According to our findings, most of the DDCFs and DDRFs that are modulated are prone to be upregulated than being downregulated.

Taken together, our results demonstrated that DDCFs and DDRFs tend to be upregulated than being downregulated in various vascular pathologies, autoimmune disorders, digestive disorders and aging diseases. In contrast, modulation of DDCFs in metabolic diseases did not reveal a distinct pattern (7 upregulated and 6 downregulated among 13 DDCFs that are modulated). Similarly, a distinct pattern could not be observed in modulation of DDRFs in metabolic disorders.

### Various human cancers upregulate the expression of DNA damage checkpoint factors, and repairing factors, with exceptions such as no changes in pancreatic and prostate cancers and downregulation in male germ cell tumors

It has been reported that DNA damages and DNA strand breaks often happen in carcinogenesis/tumorigenesis (Chang et al., 2017). We hypothesized that various cancers/tumors modulate the expression of DDCFs and DDRFs in differentially. To examine this hypothesis, we examined 12 malignancies from five systems including five digestive cancers, four reproductive cancers, one respiratory cancer, one urinary cancer and one lymphoma from hematopoietic system. As summarized in Supplementary Table 17, the gene expression changes of *DDCFs* were mostly upregulated in human colorectal carcinoma, gastric cancer, esophageal cell carcinoma, intrahepatic cancer, ovarian carcinomas, non-small cell lung cancers, clear cell renal cell carcinomas and Burkitt lymphoma. However, most of the modulated DDCF genes in adult male germ cell tumors and breast cancer tend to be attenuated in expression. Mostly, DDRFs also tend to increase in all the cancer types we analyzed except in breast cancer. In breast cancer a distinct pattern of DDRFs modulation could not be observed (3 DDRFs upregulated while 3 downregulated) (Supplementary Table 18).

Taken together, the results suggest that *first*, various human cancers significantly induce most of the DDCFs(44 out of total 58 changes, 75.9%) and DDRFs (32 out of total 45 changes, 71.1%); *second*, human ovary carcinomas show the highest induction of DDCFs and DDRFs where human male germ cell tumors induce depletion of DDCFs, suggesting that although these are all cancers in the reproductive system, the gender difference may cause differences in modulating the expression of DDCFs; *third*, unlike other cancer types we analyzed, breast cancer tends to downregulate the expression of DDCFs, suggesting that breast cancer is different from most other cancers in modulating DNA damage checkpoint and repair responses; and *fourth*, to our surprise, prostate cancer and pancreatic cancer do not induce any expression changes of DDCFs and DDRFs, suggesting that DNA damage and repair may not contribute significantly to the pathogenesis of those two cancers.

**Figure 7:**
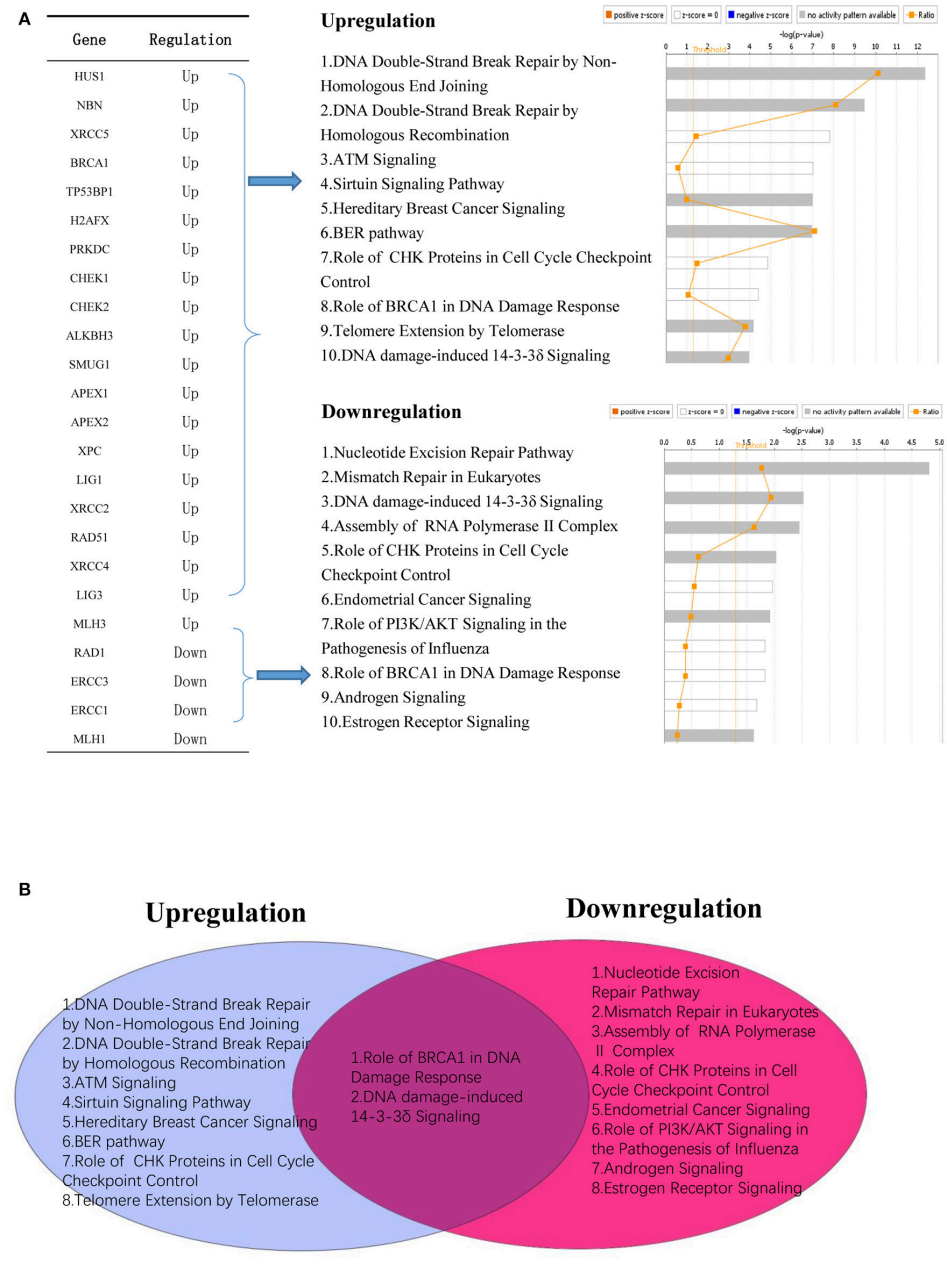
Modulated DNA response factors in cancer are involved in distinct cellular signaling networks. **(A)** Upregulated DNA damage response factors (20/68) and downregulated DNA damage response factors (4/68) modulate different signaling mechanism in human cancers. **(B)** Venn diagram shows that two signaling mechanisms are shared between upregulated and downregulated DNA damage response factors in human cancer.

In addition, we conducted a Venn analysis on significantly upregulated and downregulated DNA damage response factors in cancers. Our analysis revealed that most of these factors are involved in distinct signaling pathways. However, downregulated and upregulated factors shared BRCA1 pathway in damage response and DNA damage induced 14-3-3δ signaling pathways (Figure 7).

### Some of the inflammatory disorders that we included in our study modulate the expression changes of DDCFs and DDRFs in levels comparable to that of in cancers—a new categorization of high genomic risk inflammations

It has been reported that the incidence rates of patients with myocardial infarction (MI) to develop a malignancy within a few years of post-MI are much high than for individuals without a history of MI, implying that acute coronary syndrome or MI may promote malignancies (van Kruijsdijk et al., 2013; Alameddine et al., 2017; Hasin et al., 2017). We hypothesize that the inflammations and cancers mediated modulation of the expression of DDCFs and DDRFs can be used as an index to determine genomic risks and; this index can be used to classify inflammation and cancers into several distinct groups.

To test this hypothesis, in Table 1 we classified the 27 inflammatory disorders and 12 cancers into three groups based on their levels of modulation on the expression of DDCFs and DDRFs: (1) high genomic risk group with the modulation of >10% DDCF and DDRF genes), (2) medium genomic risk group (with the modulation of 10–5% DDCF and DDRF genes) and (3) low genomic risk group (with the modulation of >5% DDCF and DDRF genes). We found that: (1) high genomic risk group included 5 out of 27 inflammatory disorders (18.8%) and 9 out of 12 cancers (75%); (2) medium genomic risk group included 7 out of 27 inflammatory pathologies (25.9%) and 1 out of 12 cancers (8.3%); and (3) low genomic risk group included 15 out of 27 inflammatory disorders (55.6%) and 2 out of 12 cancers (16.7%). The high genomic risk group contained acute coronary syndrome/coronary artery disease, type 2 diabetes and hyperglycemia, and Hutchinson-Gilford progeria syndrome. The medium genomic risk group included Crohn's colitis, ulcerative colitis, atrophic gastritis, proliferative diabetic retinopathy, old sepsis-induced multiple organ failure, rheumatoid arthritis, osteoarthritis. Our data have demonstrated for the first time that some inflammatory disorders (12 out of 27 inflammations) modulate the expression changes of DDCFs and DDRFs in the levels equivalent to that of cancers in the high genomic risk group and medium genomic risk group.

**Table 1:** Inflammatory diseases and cancers can be classified in to diseases with high, medium, and low genomic risks.

**Disease**	**Type**	**Number (Up and down-regulated Gene)**	(Up+Down)% ^*^	**PMID**	
Coronary artery disease		18	26%	28344133	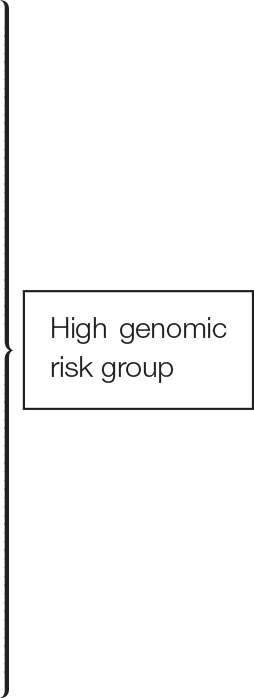
Acute coronary syndromes		7	10%		
Hutchinson-Gilford progeria syndrome	Inflammation	13	19%		
High glucose (25 mM)	(5/27, 18.8%)	9	13%	20947488	
Type 2 diabetes		7	10%		
Ovarian carcinomas		16	24%		
Esophageal squamous cell carcinoma		12	18%		
Breast cancer		12	18%		
Non-small cell lung cancers		12	18%		
Burkitt lymphoma	Cancer	12	18%		
Adult male germ cell tumors	(9/12, 75.0%)	10	15%		
Clear cell renal cell carcinomas		9	13%		
Colorectal carcinoma		7	10%		
Gastric cancer		7	10%		
Crohn's colitis		6	9%	28991855	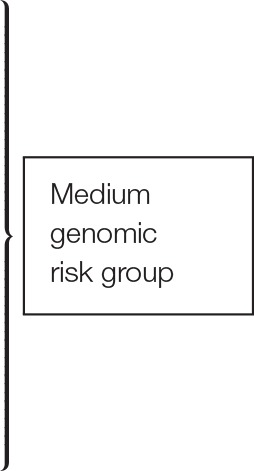
Ulcerative colitis		4	6%	27152873	
Atrophic gastritis		4	6%	26958813	
Proliferative diabetic retinopathy	Inflammation (7/27, 25.9%)	6	9%		
Old sepsis induced multiple organ failure		6	9%		
Rheumatoid arthritis		4	6%	18433475	
Osteoarthritis		4	6%		
Intrahepatic cancer	Cancer (1/12, 8.3%)	6	9%		
Aortic occlusive disease		3	4%		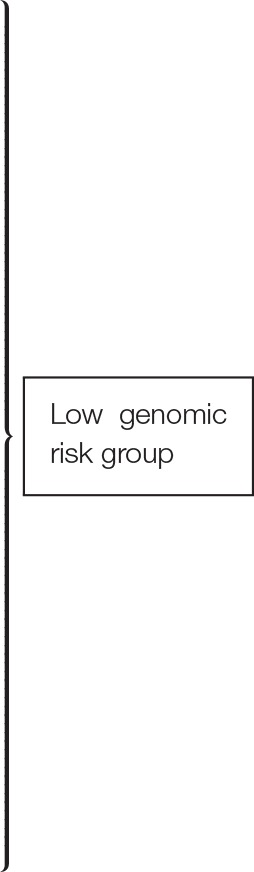
Atheroscleros		3	4%		
Oxidized LDL		3	4%		
Crohn's ileitis		3	4%		
HP-Infection with corpus-predominant atrophic gastritis		2	3%		
Alzheimer's disease		3	4%		
Old (macular retina)	Inflammation	2	3%		
Homozygous familial hypercholesterolemia	(15/27, 55.6%)	2	3%		
Psoriasis		2	3%		
Asthma patient		1	1%		
Systemic lupus erythematosus		0	0%		
Diabetic Kidney Disease		0	0%		
Type 1 diabetes		0	0%		
Metabolic syndrome		0	0%		
Homocysteine (100 μmol/L)		0	0%		
Pancreatic tumors	Cancer	0	0%		
Prostate cancer	(2/12, 16.7%)	0	0%		

*This classification is based on the modulation of DDCFs and DDRFs in the diseases condition*.

* *(Up + Down)% = number of Up and Down-regulated gene/68 in disease*.

*High genomic risk group: (Up + Down) % ≥ 10%*.

*Medium genomic risk group: 5% < (Up + Down) % < 10%*.

*Low genomic risk group: (Up + Down)% ≤ 5%*.

### The expression of oxygen-sensing genes PHD2, HIF1B, and HIF1A, VEGF pathway genes PGF and VEGFB, and stem cell master gene SOX2, and KIT have a positive correlation with DNA damage response factors gene expression

**Figure 8:**
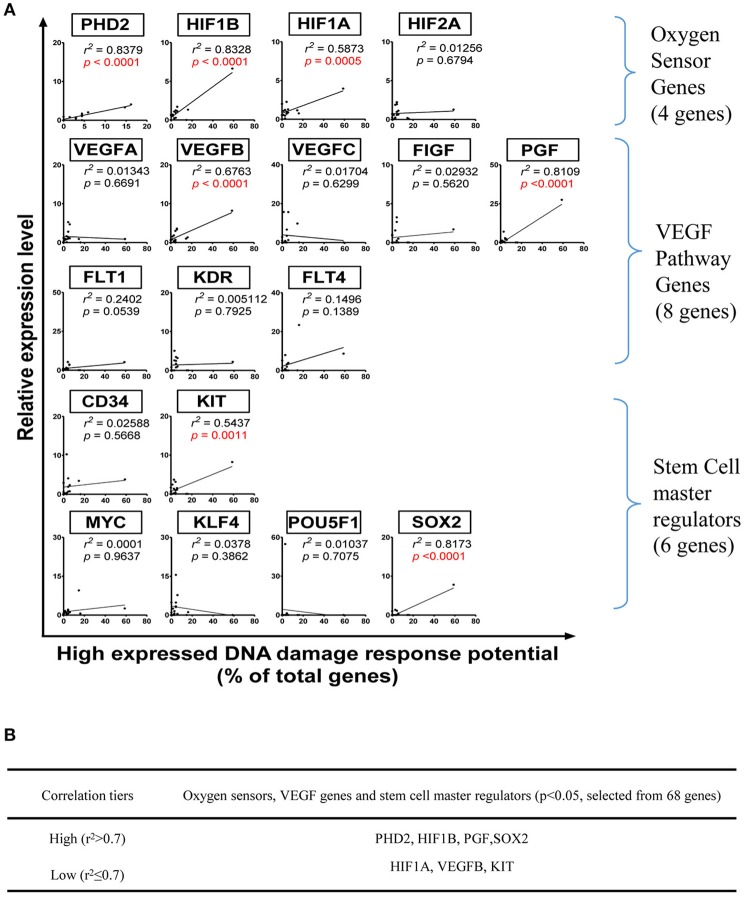
Oxygen sensors, VEGF pathway regulators and stem cell master regulators regulate DNA damage response gene expression in human tissues. (Tissues: adipose tissue, blood, bone marrow, brain, embryonic tissue, heart, kidney, liver, lung, lymph node, muscle, pancreas, skin, spleen, thymus, vascular). **(A)** Correlation between mRNA relative expression levels and specific genes that act as Oxygen Sensor, VEGF pathway genes and stem cell master regulators. **(B)** Eleven genes with *p* < 0.05 were categorized into two tiers based on *r*^2^value. PHD2, Prolyl hydroxylase domain-containing protein 2; HIF1B, Hypoxia-inducible factor-1 beta; HIF1/2A, Hypoxia-inducible factor 1/2-alpha; VEGFA/B/C, Vascular Endothelial Growth Factor A/B/C; FIGF, C-fos-induced growth factor; FLT1/4, Fms related tyrosine kinase ¼; KDR, Kinase insert domain receptor; MYC, MYC proto-oncogene; KIT, KIT proto-oncogene receptor tyrosine kinase; KLF4, Kruppel like factor 4; POU5F1, POU class 5 homeobox 1; SOX2, SRY-box 2.

To determine the relevant mechanisms underlying the regulation of DDCFs and DDRFs expression in human tissues, we examined a hypothesis that tissue physiological functional status such as hypoxia, angiogenesis and tissue regeneration pathways may regulate the expression of DDCFs and DDRFs in human tissues. Our data revealed that DDCFs and DDRFs share some key pathways (data not shown); therefore, each mechanism may modulate a few genes in specific experimental settings. In order to determine the shared pathways and mechanisms underlying DDCFs and DDRFs expression, we grouped them together as DNA damage response factors. To test this hypothesis, we performed correlation analysis for the expression of DDCFs and DDRFs with 4 oxygen sensor genes, 8 vascular endothelial growth factor (VEGF) pathway genes, 6 stem cell regulator genes and sex determining region Y-box 2 (Sox2) (Fu et al., 2017). As shown in Figure 8, among 17 genes examined, the correlation of seven genes achieved statistical significance (*p* < 0.05). The highly expressed DNA damage response potentials were highly correlated with oxygen-sensing genes PHD2, HIF1B, PGF, and stem cell master regulator gene SOX2 (high correlation *r*^2^ > 0.7). Low level correlation was observed between HIF1A, VEGFB, KIT, and highly expressed DNA damage response potentials (*r*^2^ ≤ 0.7). These results suggested that the expression of oxygen-sensing genes, PHD2, HIF1B and HIF1A, VEGF pathway genes PGF and VEGFB, and stem cell master gene SOX2, and KIT have a positive correlation with DNA damage response gene expression; and these genes may be either upstream regulators or downstream targets of DNA damage response gene signaling pathways.

### The expression of 7 and 22% of DNA damage response factors are under regulation by either hypomethylation or hypermethylation respectively, whereas the expression of the rest of the 70% of DNA damage response factors are not under regulation by Methylation

Methylation and acetylation of DNA and histone proteins are the chemical basis for epigenetics. Methylation and acetylation are sensitive to cellular metabolic status. Modification rates depend on the availability of one-carbon and two-carbon substrates (S-adenosylmethionine, acetyl-CoA). In addition, they are sensitive to demodification enzyme cofactors [α-ketoglutarate, NAD (+)] and structural analog metabolites that function as epigenetic enzyme inhibitors (e.g., S-adenosylhomocysteine, 2-hydroxyglutarate). The combined impact of nutrient abundance and metabolic enzyme expression impacts epigenetics in mammals sufficiently to drive important biological outcomes (Su et al., 2016). The free radicals produced by mitochondrial metabolism cause DNA damage, suggesting that metabolism-controlled DNA damage and epigenetic regulation are connected (Guillaumet-Adkins et al., 2017).

**Figure 9:**
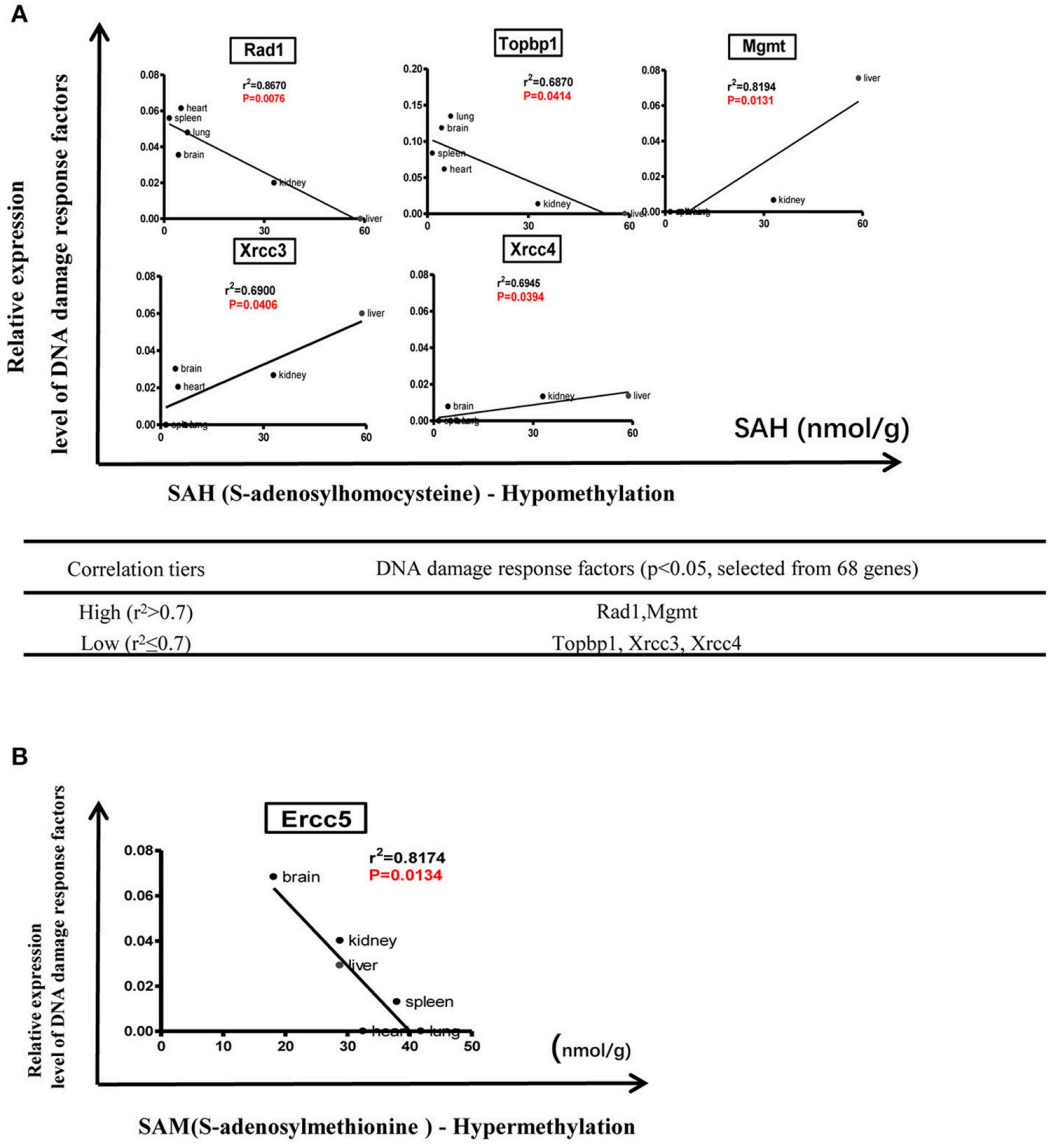
Tissue methylation status regulated by SAM levels modulate DNA damage response. **(A)** Correlation between DNA damage response factors and hypomethylation status. **(B)** Correlation between DNA damage response factors and hypermethylation status.

**Figure 10:**
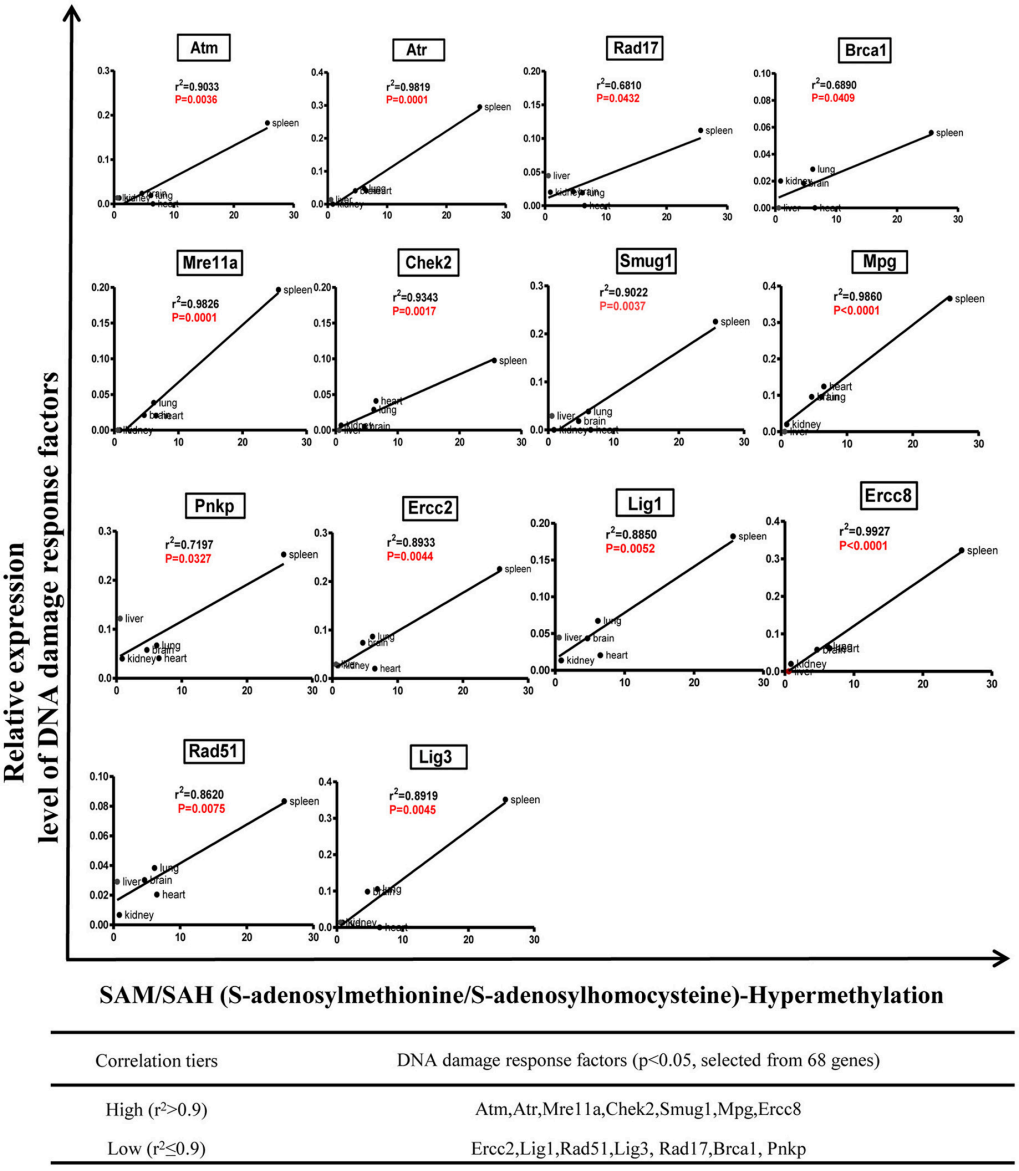
Tissue SAM/SAH ratio may regulate DNA damage response factors expression in mouse tissues.

We hypothesized that the expression of certain DDCFs and DDRFs are under epigenetic regulation such as methylation as we reported for enzyme expression of homocysteine-methionine metabolism pathways (Jamaluddin et al., 2007; Chen et al., 2010). Thus, we analyzed the correlation between mouse co-signaling receptor expression and mouse tissue methylation indices determined by the ratios between S-adenosyl methionine (SAM—the universal methyl donor)/S-adenosyl homocysteine (SAH—a methyltransferase inhibitor) in mouse tissues (Chen et al., 2010). As shown in Figure 9, we found that relative expression levels of five DNA damage response factors in mouse tissues including Rad1 (*r*^2^ = 0.8670, *P* = 0.0076), Topbp1 (*r*^2^ = 0.6870, *p* = 0.0414), Mgmt (*r*^2^ = 0.8194, *P* = 0.0131), Xrcc3 (*r*^2^ = 0.6900, *p* = 0.0406), Xrcc4 (*r*^2^ = 0.6945, *p* = 0.0394) are correlated with tissue SAH levels (hypo-methylation index). In addition, we found that relative expression levels of one DNA damage response factor Ercc5 in mouse tissues (*r*^2^ = 0.8174, *p* = 0.0134) are correlated with tissue SAM levels (hyper-methylation index) (Figure 9). Moreover, as shown in Figure 10, we found that relative expression levels of 14 DNA damage response factors in mouse tissues including Atm, Atr, Mre11a, Chek2, Smug1, Mpg, Ercc8, Ercc2, Lig1, Rad51, Lig3, Rad17, Brca1, and Pnkp are correlated with tissue SAM/SAH ratios (hyper-methylation index). These results showed that based on the methylation regulation modes, 68 DNA damage response factors can be classified into three groups: (1) hypomethylation-regulated DNA damage response factors (5 out of 68, 7.4%); (2) hypermethylation-regulated DNA damage response factors (15 out of 68, 22.1%); and (3) non-methylation-regulated DNA damage response factors (48 out of 68, 70.6%).

Finally, we examined a hypothesis that top 10 signaling pathways in regulating these three groups of genes are different. To test this hypothesis, we performed the Ingenuity pathway analysis for the top 10 pathways for each group of genes followed by the Venn diagram analysis. The results shown in Supplementary Figures 2, 3 indicate that *first*, hypermethylation may regulate DNA damage response factor expression via two specific pathways including cell cycle: G2/M DNA damage checkpoint regulation and GADD45 signaling; *second*, hypomethylation may regulate DNA damage response factor expression via four specific pathways including methionine degradation I (to homocysteine), cysteine biosynthesis III (mammal), superpathway of methionine degradation, and p53 signaling; and *third*, the majority DNA damage response factors whose expression are not under methylation regulation also have three specific pathways including nucleotide excision repair pathway, mismatch repair in eukaryotes, and type III histone/protein deacetylase sirtuin signaling pathway as we reported (Yin et al., 2015).

### Proinflammatory cytokines may induce DNA damage responses by significantly increasing the expression of DNA damage checkpoint factors and decreasing the expression of DNA repair factors

We then examined a hypothesis that various inflammatory cytokine pathways may modulate the expression of DDCFs and DDRFs. Therefore, we examined DDCFs and DDRFs expression in various cells stimulated by prototypic pro-inflammatory cytokines including tumor necrosis factor-α (TNF-α) (Yang et al., 2008), interleukin-17A (IL-17A) (Mai et al., 2016), interferon-γ (IFN-γ), IL-1β (Yin et al., 2013), IL-6, IL-33, alarmin S100A8 (myeloid-related protein 8, MRP8 Rammes et al., 1997; Averill et al., 2012; Holzinger and Roth, 2016), as well as endotoxin lipopolysaccharide (LPS) (Sha et al., 2015).

**Figure 11:**
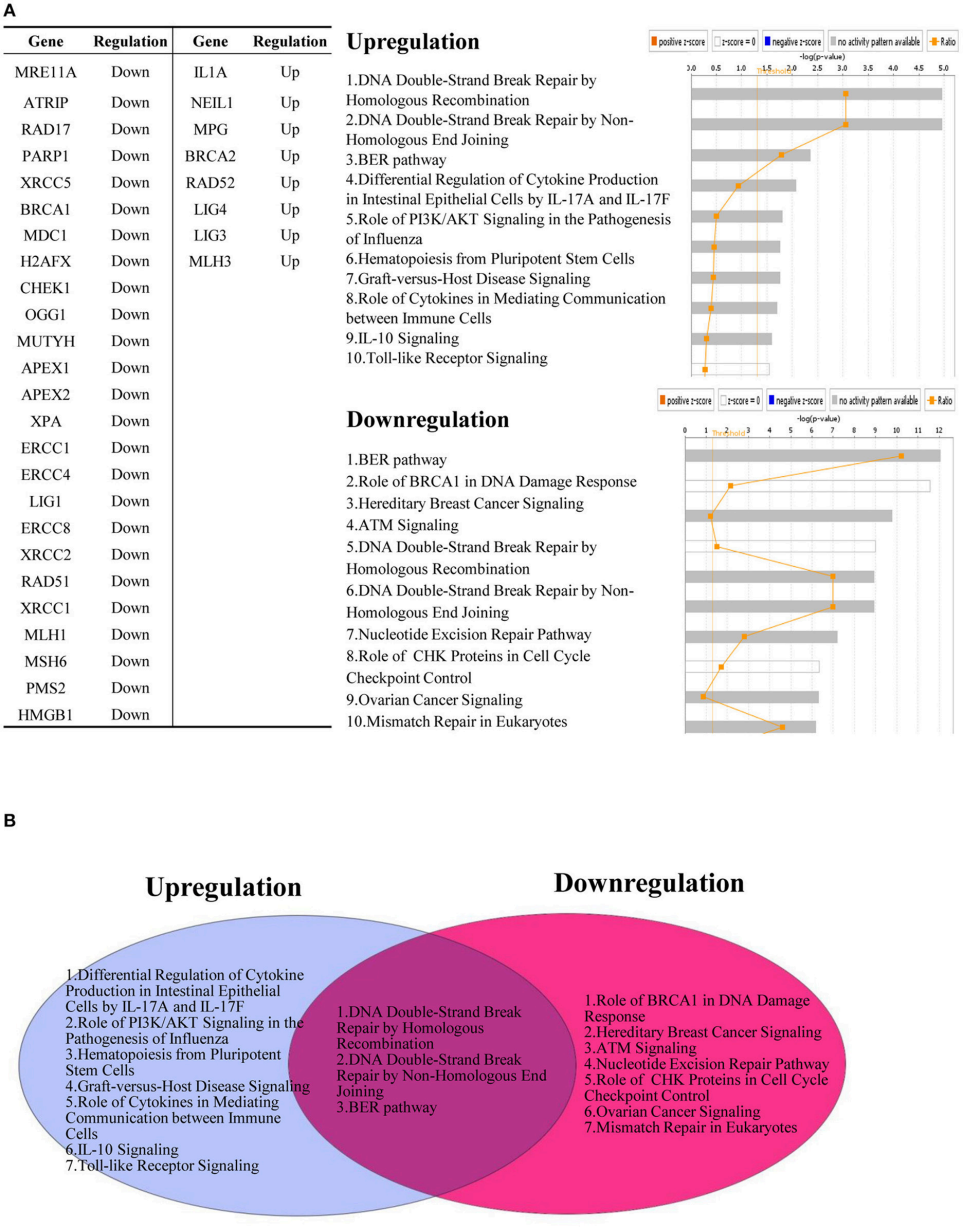
Pro-inflammatory cytokines regulate the expression of DNA damage response factors. **(A)** Downregulated DNA damage response factors (25/68) and upregulated DNA damage response factors (8/68) by cytokine treatment are involved in different signaling mechanisms. **(B)** Three pathways are shared between upregulated and downregulated DNA damage response factors after treatment with pro-inflammatory cytokines.

As shown in Supplementary Tables 19, 20, pro-inflammatory cytokines can regulate the expression of DDCFs and DDRFs. Interestingly, pro-inflammatory stimuli such as LPS, MRP8, TNF-α and IL-1β exert a profound effect on interleukin-1α in human blood cells. Compared to DDCFs, DDRFs tend to be downregulated more with pro-inflammatory stimuli, suggesting pro-inflammatory cytokines may induce DNA damage responses by significantly decreasing the expression of DDRFs. We also conducted an IPA (Ingenuity pathway analysis) on significantly upregulated and downregulated DNA damage response factors due to response to cytokine treatment. While many of the signaling pathways are specific for cytokine induced and attenuated DNA response factors, three pathways were common for both groups. These pathways are DNA DSB (double strand break) repair by homologous recombination, DNA DSB repair by non-homologous end joining, and BER pathway (Figure 11).

### Oxidative stress sensing and protective genes do not significantly modulate the expression of DDCFs and DDRFs

Inflammatory and epithelial cells release reactive oxygen (ROS) and nitrogen species (RNS), which are capable of causing DNA damage (Kawanishi et al., 2017). We hypothesized that oxidative stress induces DNA damage response by modulating the expression of DDCFs and DDRFs.

Transcription factor Nrf2 (NF-E2-related factor 2) is a master regulator of cellular responses against environmental stresses. Nrf2 induces the expression of detoxification and antioxidant enzymes and suppresses the induction of pro-inflammatory cytokine genes (Suzuki and Yamamoto, 2017). Therefore, we analyzed the expression changes of DDCFs and DDRFs in NRF2 deficient cell/tissue in comparison to that of wild-type controls. The microarray datasets we used for this analysis were conducted on mouse esophageal epithelium and liver. Our analysis revealed that the absence of Nrf2 did not induce any significant changes in the expression of DDCF except interleukin-1alpha (IL1α) (Supplementary Table 21). Further, Nrf2 deficiency downregulated the expression of a few DDRF genes (Supplementary Table 22).

Keap1 (Kelch-like ECH-associated protein 1) is an adaptor subunit of Cullin 3-based E3 ubiquitin ligase. Keap1 regulates the activity of Nrf2 and acts as a sensor for oxidative and electrophilic stresses (Suzuki and Yamamoto, 2017). Therefore, we studied the expression changes of DDCFs and DDRFs in Keap1 deficient cells/tissues microarray in comparison to that of wild-type controls. We could not detect significant gene expression changes for any of the DDCFs we included in this study. The expression of RAD23B and ERCC8 (DDRFs) were significantly downregulated in the absence of Keap1 (Supplementary Tables 21, 22).

In addition, we studied the gene expression changes of DDCFs and DDRFs in Forkhead box protein O1 (FoxO1) deficient hematopoeitc stem cells. FoxOs are transcription factors that orchestrate programs of gene expression known to control a variety of processes in response to cellular stress. The genes regulated by this family of transcription factors are involved in the regulation of cellular energy production, oxidative stress resistance and cell viability and proliferation (Link and Fernandez-Marcos, 2017). FoxO1 deletion induced the gene expression of three DDCFs. Most significantly, FoxO1 deletion induced around 12 fold changes of IL1A gene expression in hematopoietic stem cells induced DDCF IL1A. However, deletion of FoxO1 did not trigger gene expression changes of DDRFs in hematopoietic stem cells (Supplementary Tables 21, 22).

There is likely a dichotomy whereas superoxide dismutase 2 (SOD2) can be considered a protective antioxidant, as well as a pro-oxidant during cancer progression depending on the level of accumulation and detoxification of H_2_O_2_ (Ekoue et al., 2017). Therefore, we studied the expression changes of DDCFs and DDRFs in SOD2 deficient cells/tissues in comparison to that of wild-type controls. SOD2 deficiency did not affect the expression of DNA damage response factors except MSH6 (DDRF) (Supplementary Tables 21, 22).

Continuously elevated ROS levels will result in oxidative stress and development of disease, but likewise, insufficient ROS production will be detrimental to health. Reduced or even complete loss of ROS generation originates mainly from inactivating variants in genes encoding for nicotinamide adenine dinucleotide phosphate (NADPH) oxidase complexes. In particular, Nox2 oxidase function deficiency in phagocytes due to genetic variants (CYBB, CYBA, NCF1, NCF2, and NCF4) has been recognized as a direct cause of chronic granulomatous disease (CGD), an inherited immune disorder. Thus, we studied the expression changes of DDCFs and DDRFs in neutrophil cytosolic factor 1 (also known as p47-phox, NCF1) deficient blood and found that NCF1 absence does not alter the expression of DNA damage response factors relative to controls (Supplementary Tables 21, 22).

Transient increase of stressors during acute bouts of exercise or exercise training stimulate enhancement of cellular stress protection against future insults of oxidative, metabolic and mechanical stressors that could induce injury or disease, which has been termed as exercise preconditioning (EPC). EPC stimulates transcription factors such as Nrf-1 and heat shock factor-1 and up-regulate the gene expression of cytosolic (e.g., glutathione peroxidase and heat shock proteins) and mitochondrial adaptive or stress proteins [e.g., manganese superoxide dismutase, mitochondrial KATP channels and peroxisome proliferator activated receptor γ coactivator-1 (PGC-1)]. Thus, we studied the expression changes of DDCFs and DDRFs in PGC-1 deficient muscles in comparison to that of wild-type controls. We could not detect gene expression changes of any of the DNA damage response factors compared to the controls in this dataset (Supplementary Tables 21, 22).

When summarized the data shown in Supplementary Tables 21, 22, it is obvious that the oxidative stress associated gene deficiencies we studied affect changes in the expression of few DDCFs and DDRFs.

### Intracellular organelle DAMPs significantly modulate the expression of DDCFs and DDRFs

After determining the expression changes of DDCFs and DDRFs under physiological and pathological conditions, we focused on identifying the potential mechanisms that may modulate the expression of these factors under various cellular stresses. Danger associated molecular patterns (DAMP) regulate inflammation and is known to give rise to many sterile inflammatory disorders. Several previous reports including ours demonstrated that DAMP-sensor caspase-1 can cleave nuclear protein-type II histone deacetylase sirtuin-1 and promotes endothelial activation (Yin et al., 2015), suggesting caspase-1 can sense nuclear DAMPs. Additionally, presence of nuclear inflammasomes and extracellular exosome-based inflammasomes was reported, suggesting that DAMPs located in different organelles can trigger inflammation (Wang L. et al., 2016). Further, our newly proposed conditional DAMP (Wang X. et al., 2016) lysophosphatidylcholine (lysoPC) induces mitochondrial ATP generation-uncoupled mitochondrial reactive oxygen species increase and endothelial activation gene upregulation (Li et al., 2016), suggesting that mitochondrial DAMPs can activate nuclear programs for gene transcription.

Signaling between nucleus and other intracellular organelles can be of both directions. For an example, hyperhomocysteinemia induced epigenetic program stress facilitate hypomethylation that leads to mitochondrial dysfunction, which in turn “signals back” to nucleus and causes cell death (Xi et al., 2016). Therefore, we examined a novel hypothesis that intracellular organelle stress-induced DAMPs modulate the expression of DDCFs and DDRFs. Here we studied six intracellular organelle stresses: endoplasmic reticulum (ER), Golgi complex, lysosomes, endosomes, mitochondria and by activation of autophagy.

The ER stress response is triggered by a variety of conditions that disturb folding of proteins in the ER. The unfolded protein response (UPR) aims to clear unfolded proteins and restore ER homeostasis. In case where ER stress cannot be reversed, cellular functions deteriorate, leading to cell death and contribute to many diseases (Sano and Reed, 2013; Frakes and Dillin, 2017). Increased protein load not only causes an ER stress response but also causes Golgi stress response akin to UPR. Recent work has implicated eight basic Leucine Zipper (bZip) transcription factors in the regulation of protein components of the early secretory pathway are necessary to alleviate this stress. These include components of the three canonical branches of the UPR–ATF4, XBP1, and ATF6, as well as the five members of the Creb3 family of transcription factors (Fox and Andrew, 2015). Furthermore, Perk activation is required to activate UPR and subsequent ER stress. Hence, we studied the expression of DNA damage response factors in Perk-/- and Atf4-/- embryonic fibroblasts and Xbp-/- telenchephalon. Of note, in this study Atf4 was considered as a Golgi stress marker. As shown in Table 2, Perk deficiency did not affect the gene expression of DDCFs however, induced the expression of XPC, BRCA2, MLH2, and MLH3. Lig3 expression was attenuated in Perk deficient mouse fibroblasts. In contrast, Xbp deficiency did not affect the expression of DDRFs but induced the expression of BRCA1 in mouse telenchephalon. The Golgi stress marker Atf4 absence suppressed the expression of few DDCFs and induced the expression of two DDRFs.

**Table 2:** The gene expression changes of DNA damage checkpoint factors in response to intracellular organelle stress.

	**Endoplasmic reticulum stress**	**Endoplasmic reticulum stress**	**Golgilatha stress**	**Lysosome stress**	**Lysosome stress**	**Endosome stress**	**Mitochondrial stress**	**Mitochondrial stress**	**Autophagy**	**Autophagy**
GEO ID	GSE49598	GSE11322	GSE75150	GSE39621	GSE56102	GSE67227	GSE40207	GSE60413	GSE67676	GSE13512
Tissue	Mouse embryonic fibroblast	Mouse telencephalon	Mouse embryonic fibroblast	Mouselatha brain	Human embryonal carcinoma NTERA2 D/1 cells	Mouselatha liver	Mouselatha testis	Mouselatha cerebellum	Mouselatha liver	Mouselatha paneth cells
Comparison	Perk-/- vs. wild-type mice	Xbp1-/- vs. wild-type mice	Atf4-/- vs. wild-type mice	Npc1–/– vs. Npc1+/– mice	VAMP7 knockdown siRNA vs. control	Rab5-/- vs. wild-type mice	ClpP-/- vs. wild-type mice	Pink1-/- vs. wild-type mice	Atg7 conditional knock out vs. wild-type mice	Atg1611 mutant vs. wild-type mice
RAD9A										
RAD1										
HUS1										
MRE11A							0.46			
RAD50										
NBN										
RPA1										
RPA2										
RPA3										
ATRIP					3.11					
RAD17										
PARP1							2.03			
PARP2										
XRCC6										
XRCC5										
IL1A										
BRCA1		5.42	0.43	2.41		2.33		2.11		
TOPBP1							2.52			
TP53BP1					0.33					
MDC1										
H2AFX			0.42		6.37		2.6		2.23	
ATM										
ATR					6.3		2.03			0.46
PRKDC										
CHEK1			0.49			2.36				0.44
CHEK2										
Up	0	1/26	0	1/26	3/26	2/26	4/26	1/26	1/26	0
Down	0	0	3/26	0	1/26	0	1/26	0	0	2/26

*- Genes with significant expression changes (P < 0.05) are shown here. Red text stands for upregulation (FC ≥2), green text stands for downregulation (FC ≤ 0.5)*.

*Perk, protein kinase RNA-like endoplasmic reticulum (ER) kinase; Xbp1, X box-binding protein 1; Atf4, activating transcription factor 4; Npc1, Niemann Pick type C 1; Rab5, Ras-related protein Rab-5A; ClpP, ClpP caseinolytic peptidase, ATP-dependent, proteolytic subunit; Pink1, PTEN induced putative kinase 1; Atg7, Autophagy related 7; Atg16l1, Autophagy related 16-like 1; VAMP7, vesicle-associated membrane protein 7*.

Niemann-Pick C1 (NPC) disease, an autosomal recessive lipid trafficking disorder caused by loss-of-function mutations in the NPC1 gene, is characterized by progressive neurodegeneration. Lysosomes are stressed in Npc1-deficient cells, where lysosomal cathepsins are mis-localized within the cytosol. Thus, damage to lysosomal membranes by reactive oxygen species (ROS) leads to the leakage of lysosomal contents that culminates in apoptotic cell death (Chung et al., 2016). Therefore, we chose Npc1 as an indicator for lysosomal stress. Npc1 deficiency induced the expression of BRCA1 (a DDCF) and downregulated the expression of MSH3 (a DDRF) (Tables 2, 3).

**Table 3:** The gene expression changes of DNA damage repair factors in response to intracellular organelle stress.

	**Endoplasmic reticulum stress**	**Endoplasmic reticulum stress**	**Golgi stress**	**Lysosome stress**	**Lysosome stress**	**Endosome stress**	**Mitochondrial stress**	**Mitochondrial stress**	**Autophagy**	**Autophagy**
GEO ID	GSE49598	GSE11322	GSE75150	GSE39621	GSE56102	GSE67227	GSE40207	GSE60413	GSE67676	GSE13512
Tissue	Mouse embryonic fibroblast	Mouse telencephalon	Mouse embryonic fibroblast	Mouse brain	Human embryonal carcinoma NTERA2 D/1 cells	Mouse liver	Mouse testis	Mouse cerebellum	Mouse liver	Mouse paneth cells
Comparison	Perk-/- vs. wild-type mice	Xbp1-/- vs. wild-type mice	Atf4-/- vs. wild-type mice	Npc1-/- vs. Npc1+/- mice	VAMP7 knockdown siRNA vs. control	Rab5-/- vs. wild-type mice	ClpP-/- vs. wild-type mice	Pink1-/- vs. wild-type mice	Atg7 conditional knock out vs. wild-type mice	Atg1611 mutant vs. wild-type mice
MGMT										
ALKBH2										
ALKBH3										
OGG1										3.53
NEIL1										
MUTYH										
UNG							2.18			
SMUG1										
MPG										
APEX1										
APEX2										
POLB										
PNKP										
XPC	2.00		2.21				2.54			
RAD23B	0.43]						0.33			
XPA										
ERCC2										
ERCC3										
ERCC1										
ERCC5										
ERCC4							0.25	0.38		
LIG1			0.50			2.36	2.23		3.79	
ERCC8								0.43		
ERCC6										
XRCC2							0.42			2.87
XRCC3										
BRCA2	2.17									
RAD52										
RAD51						3.75	2.83		2.05	
LIG4										
NHEJ1										
XRCC4										
XRCC1										
LIG3	0.41						0.21			
MLH1										
MLH3	3.18									
MSH2	2.18						3.09			
MSH3			2.01	0.38						
MSH6										
PMS1										
PMS2							2.95			
HMGB1							3.19			
Up	4/42	0	2/42	0	0	2/42	7/42	0	2/42	2/42
Down	2/42	0	1/42	1/42	0	0	4/42	2/42	0	0

*-Genes with significant expression changes (P < 0.05) are shown here. Red text stands for upregulation (FC ≥2), green text stands for downregulation (FC ≤ 0.5)*.

*Perk, protein kinase RNA-like endoplasmic reticulum (ER) kinase; Xbp1, X box-binding protein 1; Atf4, activating transcription factor 4; Npc1, Niemann Pick type C 1; Rab5, Ras-related protein Rab-5A; ClpP, ClpP caseinolytic peptidase, ATP-dependent, proteolytic subunit; Pink1, PTEN induced putative kinase 1; Atg7, Autophagy related 7; Atg16l1, Autophagy related 16-like 1; VAMP7, vesicle-associated membrane protein 7*.

Rab5 is the most extensively analyzed member of the Rab family (a branch of the Ras superfamily of small GTPases). Rab5 is involved in the early endocytic pathway. It regulates the entry of cargo from the plasma membrane to the early endosomes (EE), generation of phosphotidylinositol-3-phosphate (PtdIns(3)P) lipid which is enriched on EE, homotypic fusion and the motility of EE on actin and microtubules tracks. In addition, it also functions in activating signaling pathways from EE (Jovic et al., 2010). Therefore, Rab5 is a potential marker for endosomal stress. When analyzed the gene expression changes of DNA damage response factors in Rab-/- mouse liver, we found that it induces the expression of two DDCFs including BRCA1. Further, Rab5 deficiency upregulated the expression of 2 DDRFs (Tables 2, 3).

Recent work uncovered a PINK1/parkin-dependent vesicle transport pathway wherein oxidized or damaged mitochondrial content are selectively delivered to the late endosome/lysosome for degradation. Syntaxin-17 is found to be a core mitochondrial canonical soluble NSF attachment protein receptor (SNARE) required for the delivery of stress-induced PINK1/parkin-dependent mitochondrial-derived vesicles (MDVs) to the late endosome/lysosome. VAMP7 forms a ternary SNARE complex with syntaxin-17 and SNAP29 to mediate MDV-endolysosome fusion (McLelland et al., 2016). Therefore, we recognized VAMP7 as another marker for lysosomal stress. Unlike Rab5, VAMP7 deficiency did not affect the expression of DDRFs. However, VAMP7-/- induced the expression of ATRIP, H2AFX and ATR (DDCFs) relative to the control (Tables 2, 3).

Mammalian CLPP has an essential role in determining the rate of mitochondrial protein synthesis by regulating the level of mito-ribosome assembly (Szczepanowska et al., 2016). Hence, we identified the CLPP as a mitochondrial stress marker. When analyzed the expression changes of DNA damage response factors in CLPP deficient mouse testis, we observed that it affects the expression of as many as 11 DDRFs. Further, absence of CLPP affected the expression of 5 DDCFS, where for of these genes were upregulated (Tables 2, 3).

In addition, we analyzed the expression changes of DNA response factors in the absence of Pink1. Pink1 (PTEN-induced putative kinase (1) and PARK2 (E3 ubiquitin protein ligase Parkinson's disease protein 2 also known as parkin) are two genes associated with familial Parkinson's disease and have a crucial role in mitophagy, partly by modulating mitochondrial fusion and fission (Andreux et al., 2013). Pink1 deletion induced BRCA1 expression in mouse cerebellum while downregulating the expression of two DDRFs (Tables 2, 3).

Selective survival could be mediated by suppression of reactive oxygen species and oxidative stress (mitophagy) or limiting ER stress-induced apoptosis (reticulophagy). By mitigating such stress, autophagy prevents DNA damage and caspase activation, but can also directly crosstalk with DNA damage machinery (Riffelmacher and Simon, 2017). Atg7 and Atg16L1 are genes that regulate autophagy in response to stress conditions in cells. Therefore, we hypothesized that the autophagy can affect the expression of DDCFs and DDRFs. Atg7 absence in mouse liver induced the expression of H2AFX (a DDCF), LIG1 (a DDRF), and RAD52 (a DDRF). Further, deletion of Atg16l1 significantly downregulated the expression of 2 DDCFs and upregulated the expression of 2 DDRFs.

Further, we conducted an IPA analysis on the induced and attenuated DNA damage response factors in response to intracellular organelle stresses. While some pathways are specific, we observed that 5 signaling pathways are common for both groups (Supplementary Figure 4).

As shown in Tables 2, 3, it can be concluded that intracellular organelle stresses can affect the expression of DNA damage response factors. Out of the organelle stresses we studied, our analysis shows that mitochondrial stress profoundly affect the gene expression of DDCFs and DDRFs. Also, we observed that the expression of DDCF BRCA1 is significantly altered in ER, Golgi, lysosomal, endosomal, and mitochondrial stresses. Therefore, BRCA1 might be an important mediator in receiving the stress signals from intracellular organelles.

## Discussion

It has been reported that chronic inflammation contributes approximately to 25% of human cancers (Kawanishi et al., 2017). Under inflammatory conditions, inflammatory cells release reactive oxygen species (ROS) and reactive nitrogen species (RNS), which cause DNA damage. In addition, DNA damage also induces inflammation and senescence (Kang et al., 2015). However, regardless of the significant progress made in the field, a comprehensive analysis of tissue expression profile of DDCFs and DDRFs under physiological conditions in human and mouse tissues was not conducted before. Further, how the expression of DDCFs and DDRFs are modulated in human pathologies such as cancers and sterile inflammatory disorders and the potential signaling mechanisms that are involved in the modulation are not fully known.

To address these significant issues, we took a panoramic, nonbiased, experimental data mining approach and analyzed many publicly available databases and microarray datasets. We have made several important findings: (1) Among 26 DNA damage checkpoint factors expressed in human 21 tissues, four factors including PARP1, XRCC6, XRCC5, and PRKDC are ubiquitously expressed; and others are differentially expressed in human tissues. (2) Among 42 DNA damage repair factors, six factors including APEX1, XPC, ERCC3, ERCC5, HMGB1, and MLH1 are ubiquitously expressed; and other DDRFs are differentially expressed in human tissues. (3) Human vascular, autoimmune, digestive, aging diseases have the tendency to upregulate than downregulate the expression of DDCFs and DDRFs. Herein, we acknowledge that the datasets that we have used to study the effect of vascular diseases on DDCFs and DDRFs expression were mostly conducted on blood cells. Thus, the expression changes we observed in the blood cells may not be directly correlated to the diseased tissues. (4) Metabolic diseases did not show a distinct pattern in gene modulation of DDCFs and DDRFs, maybe due to tissue specific responses to metabolic changes. (5) Various human cancers upregulate the expression of DDCFs and DDRFs with an exception in pancreatic cancers and prostate cancers, where overall modulation of DNA damage response factors was not observed. In contrast, downregulation of DDCFs and DDRFs were observed in male germ cell tumors. However, the gene expression changes during cancers may be more complicated than what we report here. It is highly likely that different gene expression patterns may emerge during different stages of cancer progression (Zhou et al., 2003; Garnis et al., 2004). Therefore, more studies are required to discern the patterns of DNA damage response factor expression changes during cancer progression. (6) The expression of oxygen-sensing genes, PHD2, HIF1B, and HIF1A, VEGF pathway genes PGF and VEGFB, and stem cell master gene SOX2, and KIT have a positive correlation with DNA damage response factor gene expression, suggesting that tissue oxygen levels, angiogenesis potential and stem cell regeneration are associated with DNA damage response factor gene expression. (7) The expression of 7 and 22% of DNA damage response factors are under regulation by either hypomethylation or hypermethylation, respectively, whereas the expressions of the rest of 70% of DNA damage response factors are not regulated by the tissue methylation status. This suggests that the expression of 1/3 of DNA damage response factors are under regulation by the methylation status (Jamaluddin et al., 2007). (8) Proinflammatory cytokines may induce DNA damage responses by significantly increasing the expression of DDCFs and decreasing the expressions of DDRFs, providing a direct evidence that proinflammatory cytokine pathways regulate genomic stability. (9) Oxidative stress sensing and protective genes do not significantly modulate the expression of DDCFs and DDRFs. (10) Intracellular organelle stresses significantly modulate the expression of DDCFs and DDRFs. The DDCF BRCA1 was modulated in ER, Golgi, lysosomal, endosomal, and mitochondrial stress. Further, mitochondrial stress has a profound effect on gene modulation of DNA damage response factors. This suggest that intracellular organelle stresses can relay the stress signals to the nucleus that may determine the genomic stability of the cells. Therefore, DNA damage response factors serve as converged downstream sensors for various intracellular organelle stresses and global cellular homeostasis. Thus, we propose a new classification of high genomic risk inflammatory disorders that profoundly affect the genome integrity leading to inflammatory cell death, gene mutations and carcinogenesis.

For the analysis, we used an experimental database mining approach that was pioneered and developed in our laboratory throughout the years (Yang et al., 2006; Yin et al., 2009; Li et al., 2012). By analyzing high precision sequencing data from tissue cDNA libraries, we were able to study the expression profiles of DDCFs and DDRFs in various tissues. Although these data are collected from cDNA cloning and DNA sequencing experiments rather than theoretical data derived from computer modeling, the data we reported are more applicable to biological systems. Since the gene expression sequencing tag (EST) data deposited in the NIH-NCBI-UniGene database have been established based on DNA sequencing data, the data obtained by EST database mining are more precise in providing the tissue expression profiles of genes than traditional hybridization- and primer annealing-based approaches like Northern blots and RT-PCRs (Yin et al., 2009). Of note, since the UniGene database does not have many non-tumor cell line-related gene expression data in various gene deficiencies and stimulation conditions, we analyzed micro-array based gene expression data deposited in NIH-GEO Datasets to determine the expression changes of DDCFs and DDRFs under pathological conditions.

Previous reports showed that DDCFs and DDRFs have expression differences in tissue-specific stem cells (Blanpain et al., 2011; Mandal et al., 2011). However, our findings presented in this paper were not reported previously. Therefore, our results significantly improve our understanding on the human and mouse tissues expression profiles of DDCFs and DDRFs. More importantly, we have systemically analyzed the expression changes and underlying signaling pathways of DDCFs and DDRFs in various human inflammations and cancers, which provide novel insights over previously overlooked, long-term genomic stability effects exerted by these disorders.

**Figure 12:**
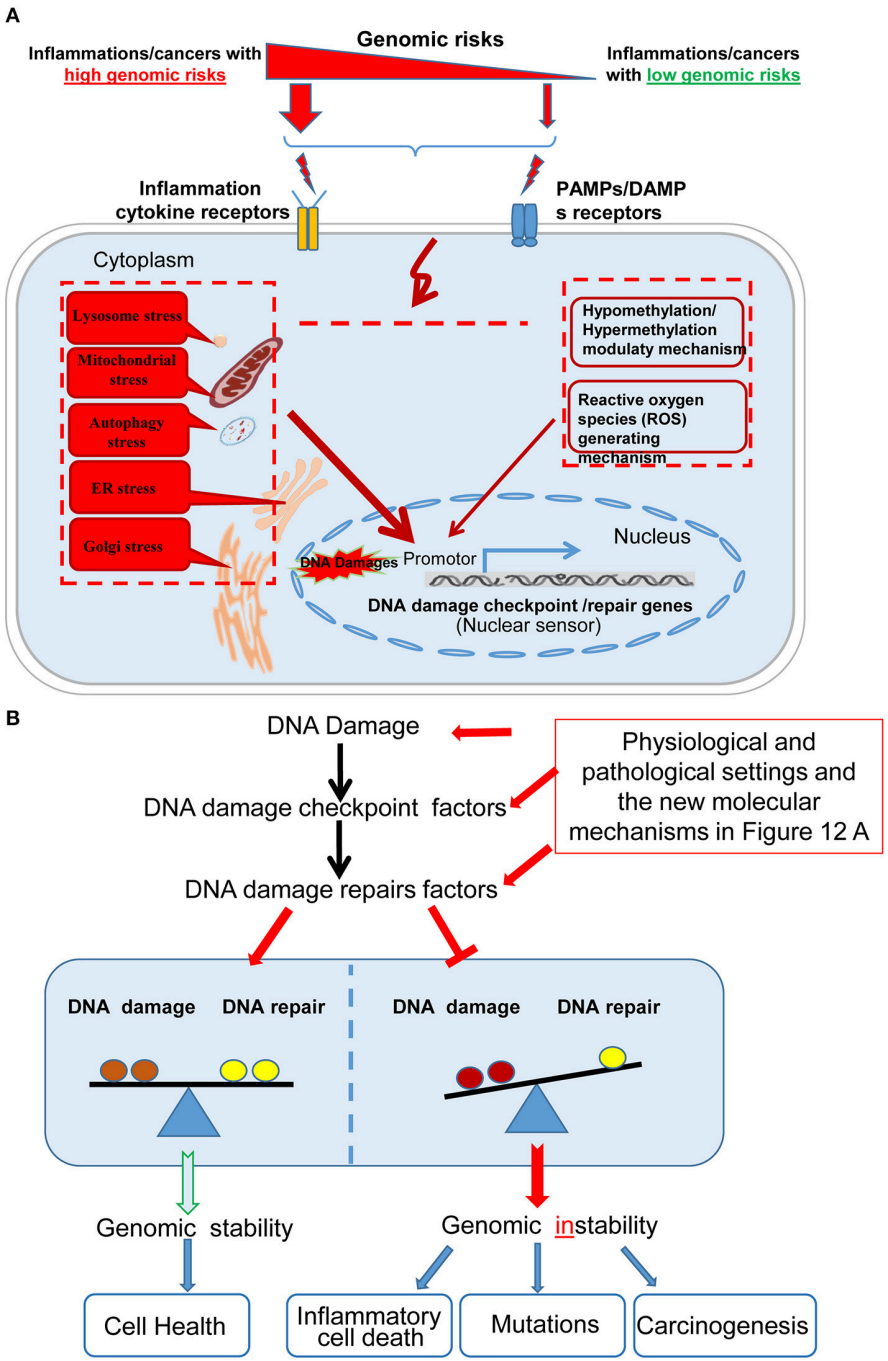
A new working model on cellular sensor cross-talking network. **(A)** Our new model integrates the following new findings: *First*, sterile inflammatory disorders and cancers can be classified into high, medium and low genomic risk groups based on the level of modulation of DNA damage response factors. Diseases-related danger associated molecular patterns (DAMPs)/pathogen associated molecular patterns (PAMPs) bind to DAMP- and PAMP-receptors respectively, leading to subsequent activation of downstream cytokine receptors to generate intracellular organelle stresses. One possible mechanism that intracellular organelle stresses may modulate DNA damage response factor expression is by regulating the methylation status of the cells and via canonical and non-canonical inflammasomes pathways. **(B)** The interplay between DDCFs and DDRFs under physiological and pathological conditions lead to either the maintenance or loss of balance between DNA damage and DNA repair mechanisms. The balance between DNA damage and DNA repair lead to maintenance of genomic stability and cell health. In contrast, increased DNA damage but decreased DNA repair would result in genomic instability and subsequent inflammatory cell death, gene mutations and carcinogenesis.

Based on our findings, we propose a new working model on inflammatory pathologies, cancers, and genomic stability (Figure 12). Our new model integrates the following new findings: *First*, in Figure 12A, the effect of inflammation and cancers on the expression of DDCFs and DDRFs can be used as an index for determining the genomic risk. By using this index, we can classify inflammatory disorders and cancers into three groups categorized as high-, medium-, and low- genomic risks. Moreover, danger associated molecular patterns (DAMPs)/pathogen associated molecular patterns (PAMPs) bind to DAMP-receptors and PAMP-receptors and subsequently to inflammation cytokine receptors and generate intracellular organelle stresses. Also, our data revealed that intracellular organelle stresses can induce DDCFs and DDRFs. This indicates that by direct and indirect mechanisms that are yet to be determined, organelle insults relay stress signals to the nucleus, which may determine the cell fate by modulating the genomic stability via modulation of DDCFs and DDRFs. This also suggest that DDCFs and DDRFs can act as nuclear sensors for intracellular organelle stresses. Though extensive experimental validation is required, our data suggest that epigenetic pathways such as hypo-/hyper-methylation pathways, canonical/non-canonical inflammasome pathways and cytokine signaling pathways are potential molecular mechanisms that may be utilized by organelles to convey the stress signals to the nucleus.

Figure 12B shows that the interplay between DDCFs and DDRFs under physiological and pathological conditions lead to either balance between DNA damage and DNA repair; or loss of the balance by increased DNA damage in the presence of decreased DNA repair. Maintaining the balance between DNA damage and DNA repair is essential to maintain genomic stability and cellular health. In contrast, increased DNA damage but decreased DNA repair will result in genomic instability and subsequently inflammatory cell death, gene mutations and carcinogenesis. This new working model has provided novel insights on DNA damage responses and DNA damage repair under various human diseases and cancers as well as novel therapeutic targets for improvement of genomic stability and cell health. Also our work suggest possible signaling mechanisms that maybe involved in transmitting organelle stress signals to the nucleus which ultimately decide the cell death by modulating DDCFs and DDRFs expression.

As mentioned above, for the first time we propose a novel concept that DDCFs and DDRFs can act as nuclear sensors (Wang L. et al., 2016) for organelle stresses, which is a part of cellular sensor cross-talking network including classical DAMP receptors such as Toll-like receptors (Yang et al., 2008), caspase-1/Nod-like receptors and inflammasomes (Yin et al., 2013), conditional DAMPs receptors and our newly proposed homeostasis-associated molecular pattern receptors (Wang X. et al., 2016; Li et al., 2017a; Wang et al., 2017; Sun et al., 2018), and mitochondrial ROS (Li et al., 2013, 2016, 2017b; Cheng et al., 2017).

## Conclusions

In summary, our study shows that most of the DDCFs and DDRFs are differentially modulated in tissues. Further, various sterile inflammatory disorders and cancers alter the gene expression of DDCFs and DDRFs. Our study also shows that DAMP mediated organelle stresses may converge to the nucleus, where the cell fate will be determined by maintaining of genomic stability. DDCFs and DDRFs may act as nuclear danger sensors for detecting intracellular organelle stresses. Therefore, the DDCFs and DDRFs that are modulated in human inflammation and cancers may serve as novel biomarkers for diagnosis and prognosis, and novel therapeutic targets for treatment of these diseases.

## Author contributions

HZ carried out the data gathering, data analysis, figures/tables preparations, and manuscript writing. Other authors provided material input and helped revising the manuscript. XY supervised the experimental design, data analysis. All authors read and approved the final manuscript.

### Conflict of interest statement

The authors declare that the research was conducted in the absence of any commercial or financial relationships that could be construed as a potential conflict of interest.

## Footnotes

^1^NIH-NCBI GEO DataSets database.
