# Supplementary material for: DNA Checkpoint and Repair Factors Are Nuclear Sensors for Intracellular Organelle Stresses—Inflammations and Cancers Can Have High Genomic Risks
Source: Front Physiol. 2018 May 11;9:516. doi: 10.3389/fphys.2018.00516 (PMC5958474; doi:10.3389/fphys.2018.00516)
Supplement: Supplementary file 1 [file Presentation_1.PDF]

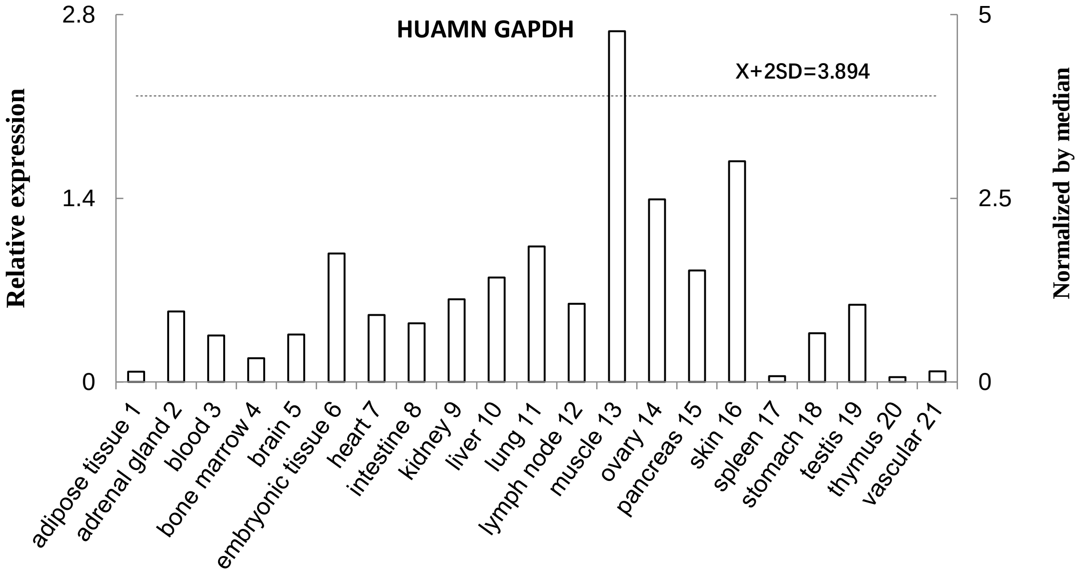

Supplementary Figure 1. The human GAPDH gene expression profiles are presented based on the expression sequence tag (EST) data in the NIH/UniGene database after normalized with the β-actin and three other house keeping gene data. The method of analyzing tissue EST profile: Data presentation format as presented in X-,Y axis and tissue order is applied to all the genes examined. As an example, the gene expression profile of human house-keeping gene Glyceraldehyde-3-phosphate dehydrogenase (GAPDH, Hs.544577) in 21 human tissues are shown on the X-axis. The gene expression data are normalized by the β-actin (Hs. 520640) expression data from the same tissue, which are presented on the left Y-axis. The expression ratios among tissues are generated by normalizing the arbitrary units of the gene in the tissues with the median level of the arbitrary units of the gene in all the tissues, which are presented on the right Y-axis. In order to define confidence intervals for statistically higher expression levels of given genes, we calculate the confidence intervals of tissue expression [the mean X + 2x standard deviations (SD) = 3.894] for three house-keeping genes including GAPDH, ARHGDIA (Hs.159161), and ribosomal protein S27a (RPS27A Hs.311640). The expression variations of given genes in tissues, when they are larger than 3.894-fold, are defined as the high expression levels with statistical significance (the right Y-axis). The same strategy applies to mouse genes among 20 tissues. Three mouse house keeping genes include Lactate dehydrogenase A (Ldha, Mm. 29324), non-POU-domain-containing octamer binding protein (Nono, Mm. 280069), and ribosomal protein L32 (Rpl32, Mm. 104368). The mean X + 2xSD = 4.162 is determined as the confidence interval for mouse gene expression.

Gene	Methylation	Gene	Methylation	Gene	Methylation
RAD9A	No change	APEX1	No change	ATM	Hypermethylation
HUS1	No change	APEX2	No change	ATR	Hypermethylation
RAD50	No change	POLB	No change	MRE11 A	Hypermethylation
NBN	No change	XPC	No change	CHEK2	Hypermethylation
RPA1	No change	RAD23B	No change	SMUG1	Hypermethylation
RPA2	No change	XPA	No change	MPG	Hypermethylation
RPA3	No change	ERCC3	No change	ERCC8	Hypermethylation
ATRIP	No change	ERCC1	No change	ERCC2	Hypermethylation
PARP1	No change	ERCC4	No change	LIG1	Hypermethylation
PARP2	No change	ERCC6	No change	RAD51	Hypermethylation
XRCC6	No change	XRCC2	No change	LIG3	Hypermethylation
XRCC5	No change	BRCA2	No change	RAD17	Hypermethylation
IL1A	No change	RAD52	No change	BRCA1	Hypermethylation
TP53BP1	No change	LIG4	No change	PNKP	Hypermethylation
MDC1	No change	NHEJ1	No change	ERCC5	Hypermethylation
H2AFX	No change	XRCC1	No change	RAD1	Hypomethylation
PRKDC	No change	MLH1	No change	MGMT	Hypomethylation
CHEK1	No change	MLH3	No change	TOPBP1	Hypomethylation
ALKBH2	No change	MSH2	No change	XRCC3	Hypomethylation
ALKBH3	No change	MSH3	No change	XRCC4	Hypomethylation
OGG1	No change	MSH6	No change		
NEIL1	No change	PMS1	No change		
MUTYH	No change	PMS2	No change		
UNG	No change	HMGB1	No change		

Hypermethylation

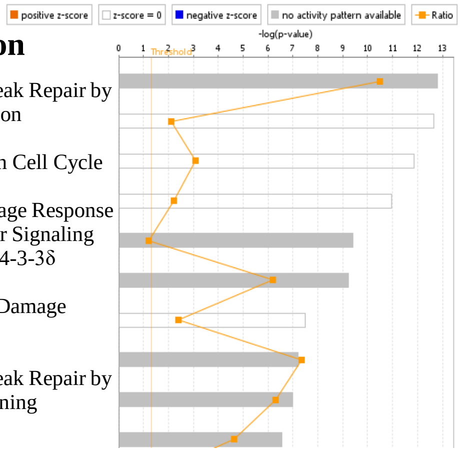

1.DNA Double-Strand Break Repair by Homologous Recombination
2.ATM Signaling
3.Role of CHK Proteins in Cell Cycle Checkpoint Control
4.Role of BRCA1 in Damage Response
5.Hereditary Breast Cancer Signaling
6.DNA damage-induced 14-3-3δ Signaling
7.Cell Cycle:G2/M DNA Damage Checkpoint Regulation
8.BER pathway
9.DNA Double-Strand Break Repair by Non-Homologous End Joining
10.GADD45 Signaling

Hypomethylation

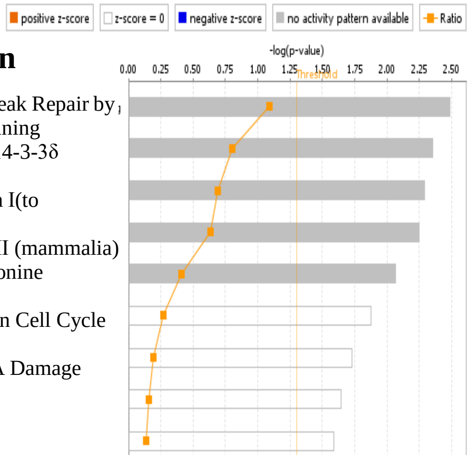

1.DNA Double-Strand Break Repair by Non-Homologous End Joining
2.DNA damage-induced 14-3-3δ Signaling
3.Methionine Degradation I(to Homocysteine)
4.Cysteine Biosynthesis III (mammalia)
5.Superpathway of Methionine Degradation
6.Role of CHK Proteins in Cell Cycle Checkpoint Control
7.Role of BRCA1 in DNA Damage Response
8.ATM Signaling
9.P53 signaling

No change

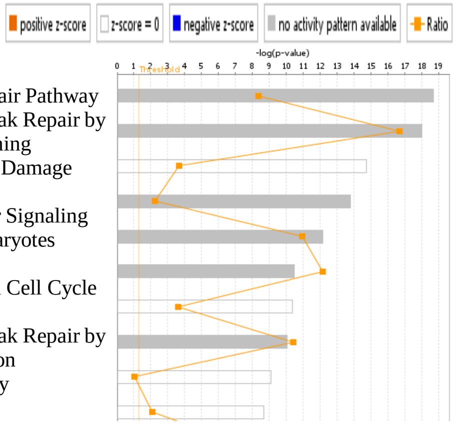

1.Nucleotide Excision Repair Pathway
2.DNA Double-Strand Break Repair by Non-Homologous End Joining
3.Role of BRCA1 in DNA Damage Response
4.Hereditary Breast Cancer Signaling
5.Mismatch Repair in Eukaryotes
6.BER pathway
7.Role of CHK Proteins in Cell Cycle Checkpoint Control
8.DNA Double-Strand Break Repair by Homologous Recombination
9.Sirtuin Signaling Pathway
10.ATM Signaling

Supplementary figure 2. Tissue SAM level and SAM/SAH ratio may regulate 15 DNA damage response factors expression, and SAH mediated hypomethylation levels may regulate expression of 5 factors in mouse tissues. Expression of 48 factors are not regulated by tissue methylation status.

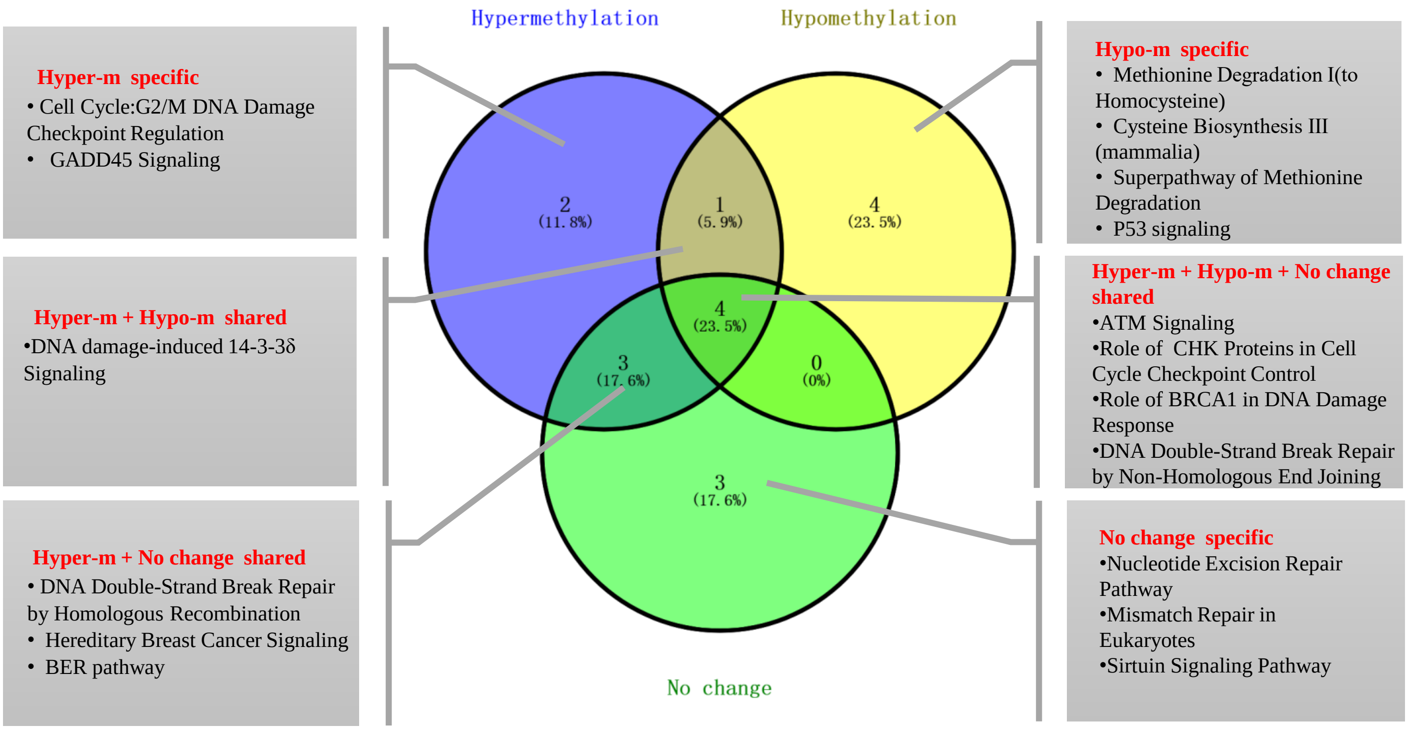

Supplementary figure 3. DNA damage response factors that are regulated by SAM mediated hypermethylation, SAM/SAH ratio and SAH mediated hypomethylation are involved in specific signaling pathways.

Gene	Regulation
ATRIP	Up
PARP1	Up
TOPBP1	Up
OGG1	Up
UNG	Up
XPC	Up
BRCA2	Up
RAD51	Up
MLH3	Up
MSH2	Up
PMS2	Up
HMGB1	Up
MRE11A	Down
TP53BP1	Down
RAD23B	Down
ERCC4	Down
ERCC8	Down
LIG3	Down

Upregulation

1.Role of BRCA1 in DNA Damage Response
2.Hereditary Breast Cancer Signaling
3.Ovarian Cancer Signaling
4.BER pathway
5.DNA Double-Strand Break Repair by Homologous Recombination
6.Mismatch Repair in Eukaryotes
7.Sirtuin Signaling Pathway
8ATM Signaling
9.Pancreatic Adenocarcinoma Signaling
10.DNA Double-Strand Break Repair by Non-Homologous End Joining

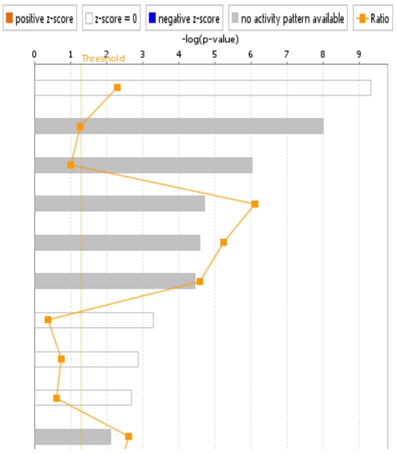

Downregulation

1.Nucleotide Excision Repair Pathway
2.DNA Double-Strand Break Repair by Homologous Non-Recombination
3.ATM Signaling
4.BER pathway
5.DNA Double-Strand Break Repair by Homologous Recombination
6.Telomere Extension by Telomerase
7.Role of CHK Proteins in Cell Cycle Checkpoint Control
8.Role of BRCA1 in DNA Damage Response
9.Hereditary Breast Cancer Signaling

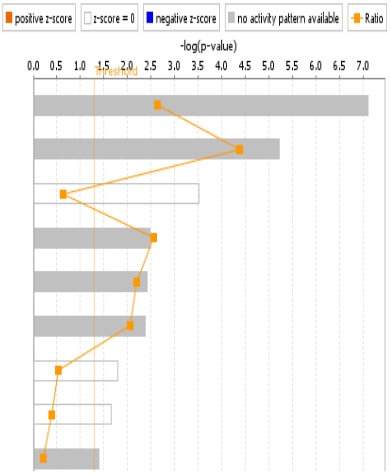

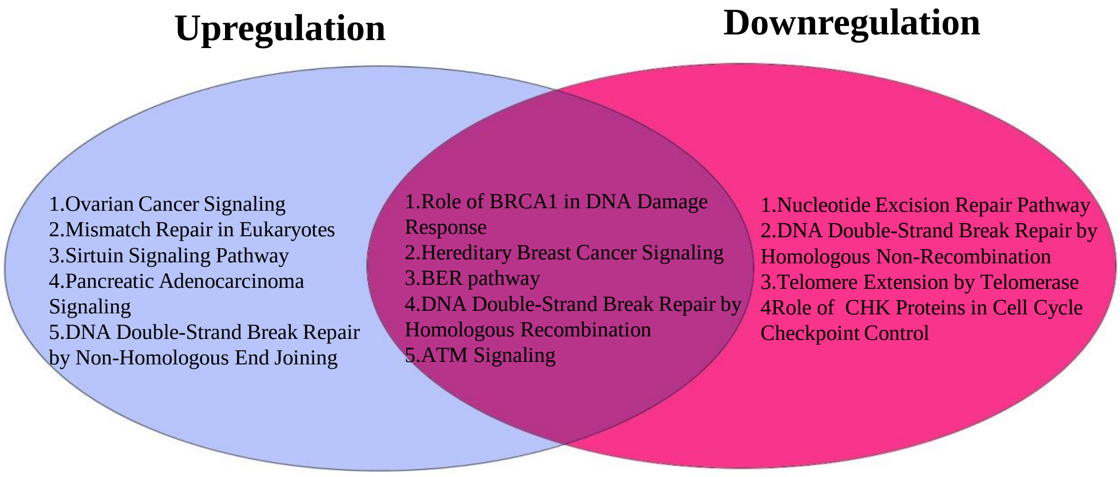

Supplementary figure 4. The deficiencies of intracellular organelle stress markers (stress responses in the ER, Golgi, lysosomes, endosomes, mitochondria and autophagy) modulate DNA damage response factors gene expression. A) 17.6% (12/68) and 8.8% (6/68) DNA response factors were upregulated and downregulated respectively in the absence of intracellular organelle stress markers. **B)** The upregulated and downregulated DNA response factors due to deletion of intracellular organelle stress markers share five signaling pathways.

Supplementary figure 5. mRNA distribution profiles of DNA damage checkpoint factors and DNA damage repair factors are differently expressed in human tissues.

A. mRNA distribution profiles of DNA damage checkpoint factors in human tissues.

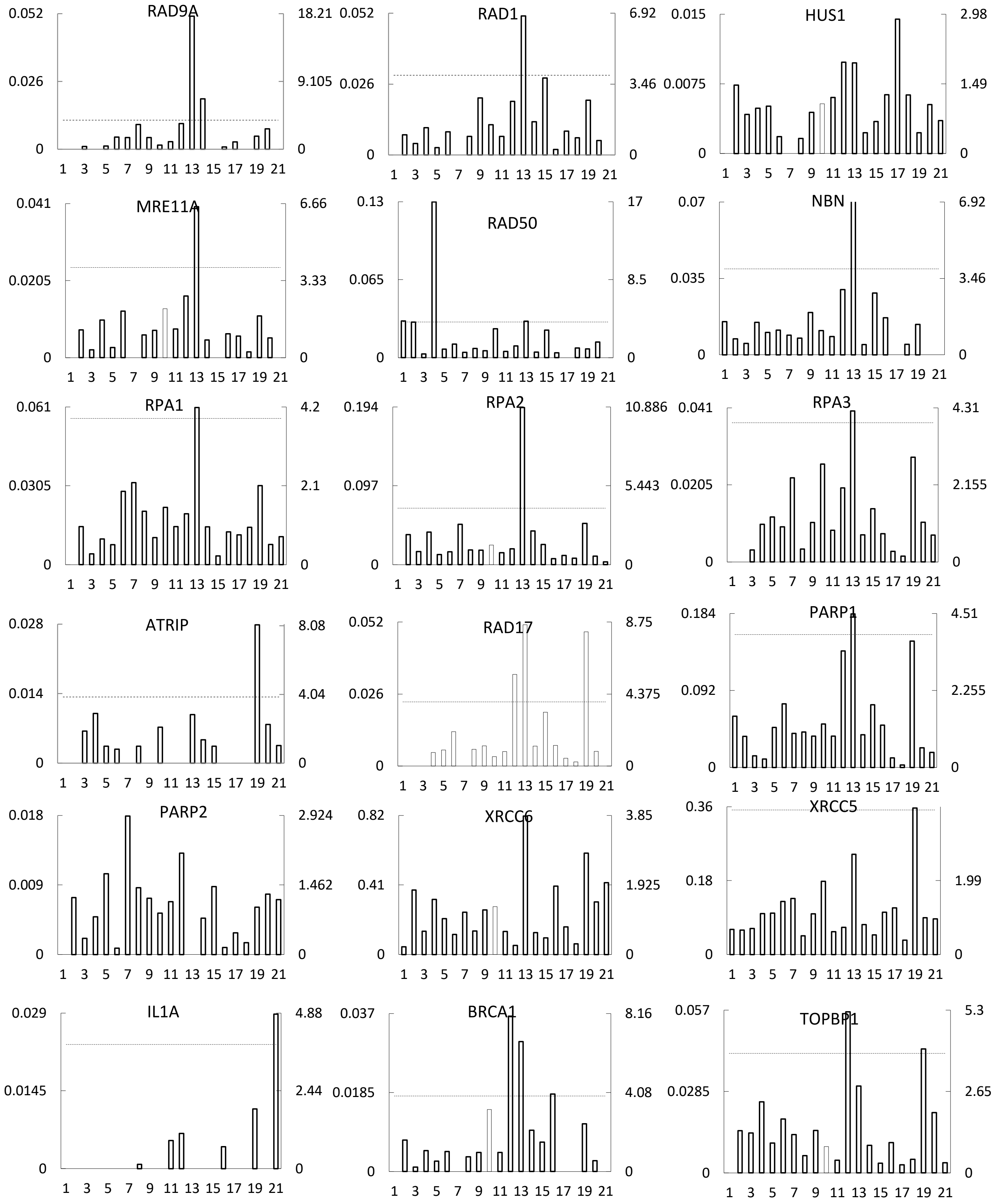

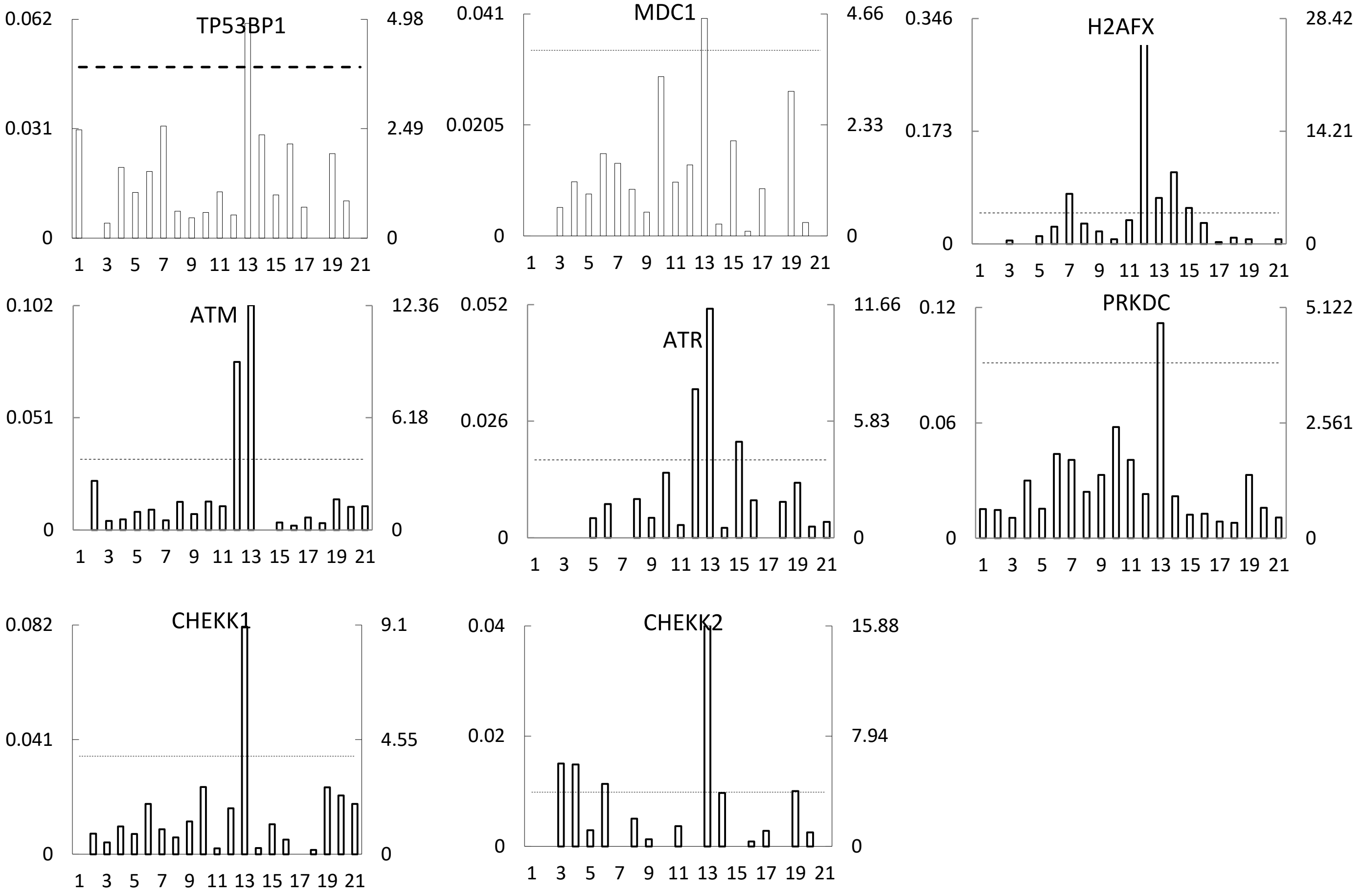

B. mRNA distribution profiles of DNA damage repair factors in human tissues

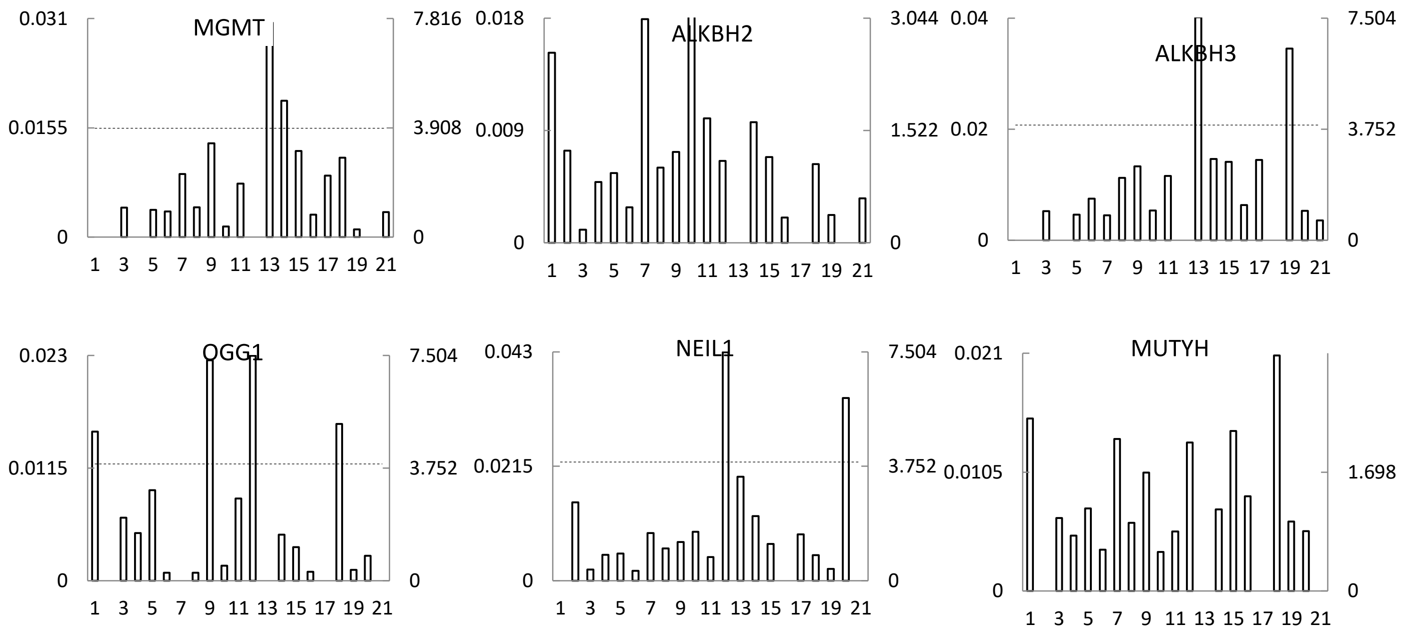

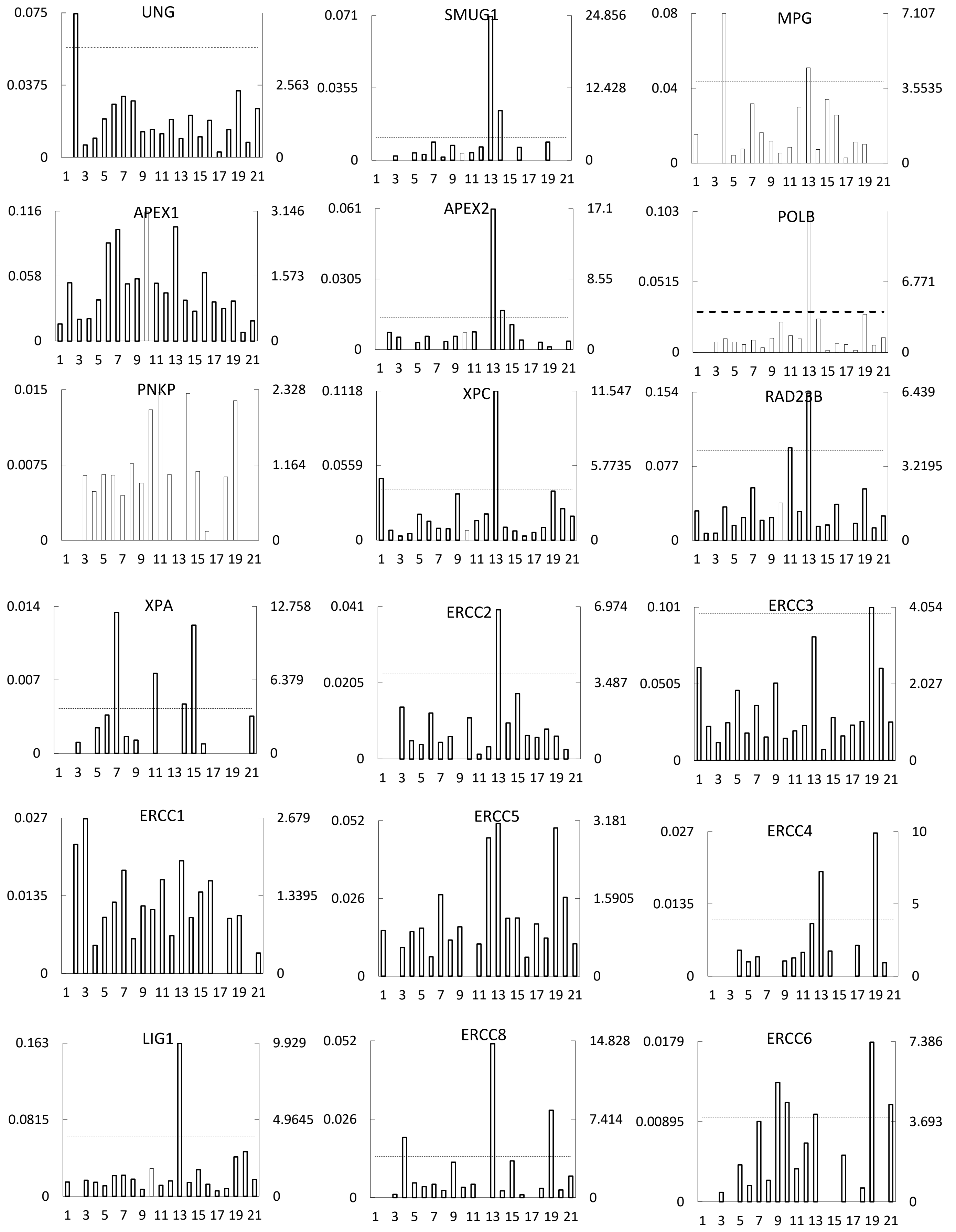
UNG
0.075
0.0375
0
2.563
0
1 3 5 7 9 11 13 15 17 19 21
SMUG1
0.071
0.0355
0
24.856
12.428
0
1 3 5 7 9 11 13 15 17 19 21
MPG
0.08
0.04
0
7.107
3.5535
0
1 3 5 7 9 11 13 15 17 19 21
APEX1
0.116
0.058
0
3.146
1.573
0
1 3 5 7 9 11 13 15 17 19 21
APEX2
0.061
0.0305
0
17.1
8.55
0
1 3 5 7 9 11 13 15 17 19 21
POLB
0.103
0.0515
0
6.771
0
1 3 5 7 9 11 13 15 17 19 21
PNKP
0.015
0.0075
0
2.328
1.164
0
1 3 5 7 9 11 13 15 17 19 21
XPC
0.1118
0.0559
0
11.547
5.7735
0
1 3 5 7 9 11 13 15 17 19 21
RAD23B
0.154
0.077
0
6.439
3.2195
0
1 3 5 7 9 11 13 15 17 19 21
XPA
0.014
0.007
0
12.758
6.379
0
1 3 5 7 9 11 13 15 17 19 21
ERCC2
0.041
0.0205
0
6.974
3.487
0
1 3 5 7 9 11 13 15 17 19 21
ERCC3
0.101
0.0505
0
4.054
2.027
0
1 3 5 7 9 11 13 15 17 19 21
ERCC1
0.027
0.0135
0
2.679
1.3395
0
1 3 5 7 9 11 13 15 17 19 21
ERCC5
0.052
0.026
0
3.181
1.5905
0
1 3 5 7 9 11 13 15 17 19 21
ERCC4
0.027
0.0135
0
10
5
0
1 3 5 7 9 11 13 15 17 19 21
LIG1
0.163
0.0815
0
9.929
4.9645
0
1 3 5 7 9 11 13 15 17 19 21
ERCC8
0.052
0.026
0
14.828
7.414
0
1 3 5 7 9 11 13 15 17 19 21
ERCC6
0.0179
0.00895
0
7.386
3.693
0
1 3 5 7 9 11 13 15 17 19 21

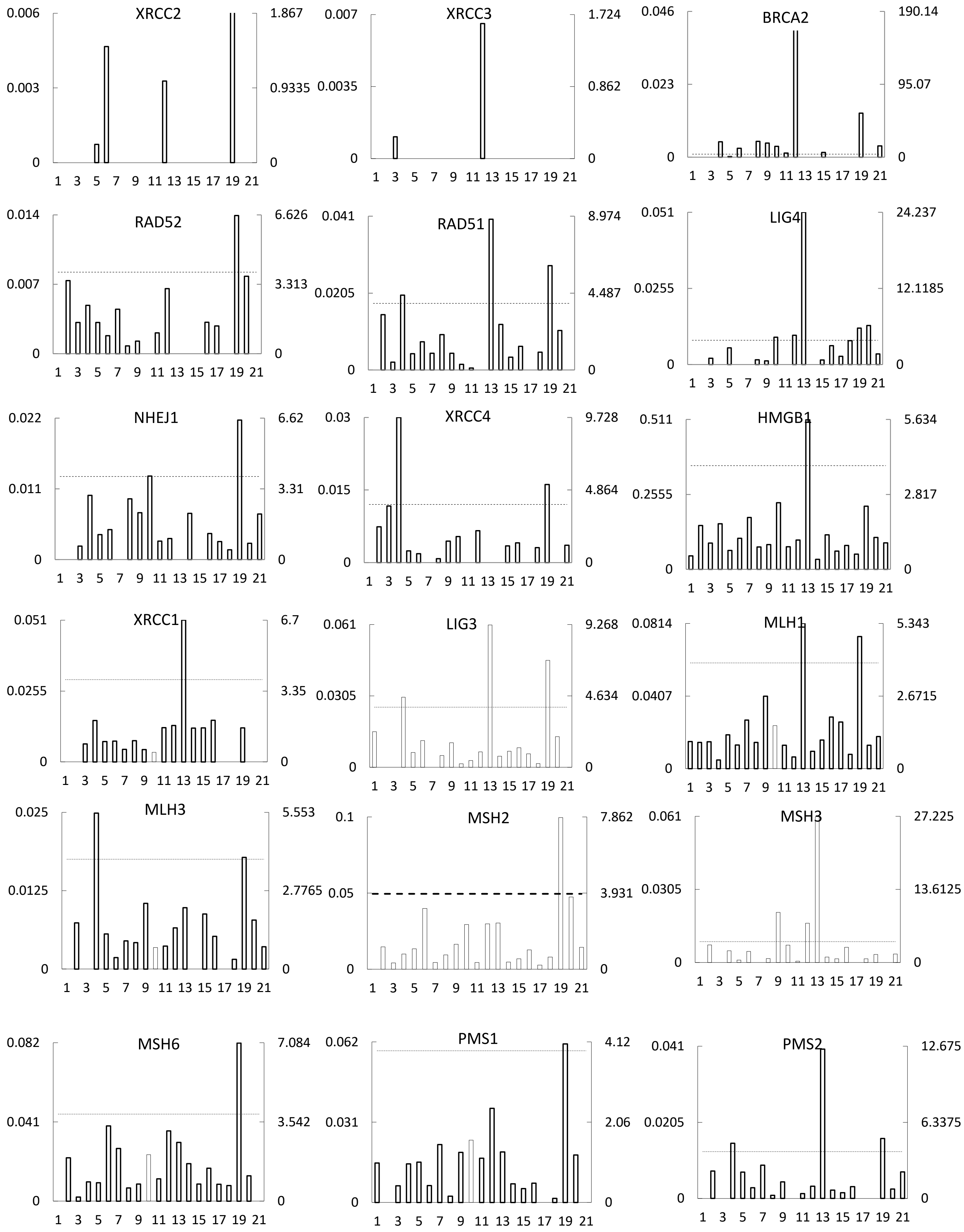
XRCC2
0.006
0.003
0
1.867
0.9335
0
1 3 5 7 9 11 13 15 17 19 21
XRCC3
0.007
0.0035
0
1.724
0.862
0
1 3 5 7 9 11 13 15 17 19 21
BRCA2
0.046
0.023
0
190.14
95.07
0
1 3 5 7 9 11 13 15 17 19 21
RAD52
0.014
0.007
0
6.626
3.313
0
1 3 5 7 9 11 13 15 17 19 21
RAD51
0.041
0.0205
0
8.974
4.487
0
1 3 5 7 9 11 13 15 17 19 21
LIG4
0.051
0.0255
0
24.237
12.1185
0
1 3 5 7 9 11 13 15 17 19 21
NHEJ1
0.022
0.011
0
6.62
3.31
0
1 3 5 7 9 11 13 15 17 19 21
XRCC4
0.03
0.015
0
9.728
4.864
0
1 3 5 7 9 11 13 15 17 19 21
HMGB1
0.511
0.2555
0
5.634
2.817
0
1 3 5 7 9 11 13 15 17 19 21
XRCC1
0.051
0.0255
0
6.7
3.35
0
1 3 5 7 9 11 13 15 17 19 21
LIG3
0.061
0.0305
0
9.268
4.634
0
1 3 5 7 9 11 13 15 17 19 21
MLH1
0.0814
0.0407
0
5.343
2.6715
0
1 3 5 7 9 11 13 15 17 19 21
MLH3
0.025
0.0125
0
5.553
2.7765
0
1 3 5 7 9 11 13 15 17 19 21
MSH2
0.1
0.05
0
7.862
3.931
0
1 3 5 7 9 11 13 15 17 19 21
MSH3
0.061
0.0305
0
27.225
13.6125
0
1 3 5 7 9 11 13 15 17 19 21
MSH6
0.082
0.041
0
7.084
3.542
0
1 3 5 7 9 11 13 15 17 19 21
PMS1
0.062
0.031
0
4.12
2.06
0
1 3 5 7 9 11 13 15 17 19 21
PMS2
0.041
0.0205
0
12.675
6.3375
0
1 3 5 7 9 11 13 15 17 19 21

Supplementary table 1. The 26 human DNA Damage checkpoint factors including sensors, mediators and transducers are focused in this study.*

		Gene name (Full name)	Gene Symbol	Function	UniGene▲ ID Human (Hs.)	UniGene▲ ID Mouse (Mm.)	PMID
1	RAD9	chromatin-binding protein RAD9	RAD9A		655354	277629	
2	RAD1	RAD1 Checkpoint DNA Exonuclease	RAD1		38114	38376	
3	HUS1	HUS1 checkpoint homolog	HUS1		152983	42201	
4	MRE11	meiotic recombination 11 homolog A	MRE11A		192649	149071	
5	RAD50	RAD50 homolog	RAD50		633509	4888	
6	NBS1	Nibrin	NBN		492208	20866	
7	RPA1	Replication protein A1	RPA1		461925	180734	
8	RPA2	Replication protein A2	RPA2		79411	2870	21211780 16498454 16314342
9	RPA3	Replication protein A3	RPA3	Sensors	487540	29073	
10	ATRIP	ATR interacting protein	ATRIP		694840	100622	
11	RAD17	RAD17 checkpoint clamp loader component	RAD17		16184	248489	
12	PARP1	Poly(rC)-binding protein 1	PARP1		177766	277779	
13	PARP2	Poly(rC)-binding protein 2	PARP2		409412	281482	
14	Ku70	X-ray repair cross complementing 6	XRCC6		292493	288809	
15	Ku80	X-Ray Repair Cross Complementing 5	XRCC5		388739	246952	
16	IL-1a	Interleukin 1, alpha	IL1A		1722	15534	27890472 26439902
17	BRCA1	Breast cancer 1	BRCA1		194143	244975	
18	TopBP1	topoisomerase II binding protein 1	TOPBP1		593379	259893	
19	53BP1	P53 binding protein 1	TP53BP1	Mediators	440968	481841	
20	MDC1	mediator of DNA damage checkpoint protein 1	MDC1		653495	218511	
21	H2A.X	H2A histone family, member X	H2AFX		477879	245931	21211780 16498454 16314342
22	ATM	ataxia telangiectasia mutated	ATM		367437	5088	
23	ATR	Ataxia telangiectasia and Rad3 related	ATR		271791	212462	
24	DNA-PK	DNA-activated, catalytic polypeptide	PRKDC	Transducers	491682	71	
25	CHK1	Checkpoint kinase 1	CHEK1		24529	16753	
26	CHK2	Checkpoint kinase 2	CHEK2		291363	279308	

* This list represents the detailed information shown in Figure 2 note, the effectors in Figure 2 were not included in the analysis as they have downstream functions that are outside the scope of DNA damage checkpoints.

▲ UniGene website: www.ncbi.nlm.nih.gov/unigene

Supplementary table 2. Among 26 DNA damage checkpoint factors, four factors including PARP1, XRCC6, XRCC5 and PRKDC are ubiquitously expressed, and others are differentially expressed in human tissues.

Human Tissues (Number: 21)

Gene	Adipose tissue	Adrenal gland	Blood	Bone marrow	Brain	Embryonic tissue	Heart	Intestine	Kidney	Liver	Lung	Lymph node	Muscle	Ovary	Pancreas	Skin	Spleen	Stomach	Testis	Thymus	Vascular	H(%)	L(%)	H+L(%)
**RAD9A**			+		+	+	+	+	+	+	+	+	++	++		+	+		+	+		**9.5**	**61.9**	**71.4**
**RAD1**		+	+	+	+	+		+	+	+	+	+	++	+	+	+	+	+	+	+		**4.8**	**81.0**	**85.7**
**HUS1**		+	+	+	+	+		+	+	+	+	+	+	+	+	+	+	+	+	+	+	**0.0**	**90.5**	**90.5**
**MRE11A**		+	+	+	+	+		+	+	+	+	+	++	+		+	+	+	+	+		**4.8**	**76.2**	**81.0**
**RAD50**	++	+	+	++	+	+	+	+	+	+	+	+	++	+	+	+		+	+	+		**14.3**	**76.2**	**90.5**
**NBN**	+	+	+	+	+	+	+	+	+	+	+	+	++	+	+	+		+	+			**4.8**	**81.0**	**85.7**
**RPA1**		+	+	+	+	+	+	+	+	+	+	+	++	+	+	+	+	+	+	+	+	**4.8**	**90.5**	**95.2**
**RPA2**		+	+	+	+	+	+	+	+	+	+	+	++	+	+	+	+	+	+	+	+	**4.8**	**90.5**	**95.2**
**RPA3**			+	+	+	+	+	+	+	+	+	+	++	+	+	+	+	+	+	+	+	**4.8**	**85.7**	**90.5**
**ATRIP**			+	+	+	+		+		+			+	+	+				++	+	+	**4.8**	**52.4**	**57.1**
**RAD17**				+	+	+		+	+	+	+	++	++	+	+	+	+	+	++	+		**14.3**	**61.9**	**76.2**
**PARP1**	+	+	+	+	+	+	+	+	+	+	+	+	++	+	+	+	+	+	+	+	+	**4.8**	**95.2**	**100.0**
**PARP2**		+	+	+	+	+	+	+	+	+	+	+		+	+	+	+	+	+	+	+	**0.0**	**90.5**	**90.5**
**XRCC6**	+	+	+	+	+	+	+	+	+	+	+	+	+	+	+	+	+	+	+	+	+	**0.0**	**100.0**	**100.0**
**XRCC5**	+	+	+	+	+	+	+	+	+	+	+	+	+	+	+	+	+	+	++	+	+	**4.8**	**95.2**	**100.0**
**IL1A**								+			+	+				+			+		++	**4.8**	**23.8**	**28.6**
**BRCA1**		+	+	+	+	+		+	+	+	+	++	++	+	+	++			+	+		**14.3**	**61.9**	**76.2**
**TOPBP1**		+	+	+	+	+	+	+	+	+	+	++	+	+	+	+	+	+	++	+	+	**9.5**	**85.7**	**95.2**
**TP53BP1**	+		+	+	+	+	+	+	+	+	+	+	++	+	+	+	+		+	+		**4.8**	**81.0**	**85.7**
**MDC1**			+	+	+	+	+	+	+	+	+	+	++	+	+	+	+		+	+		**4.8**	**76.2**	**81.0**
**H2AFX**			+		+	+	++	+	+	+	+	++	++	++	++	+	+	+	+		+	**23.8**	**57.1**	**81.0**
**ATM**		+	+	+	+	+	+	+	+	+	+	++	++		+	+	+	+	+	+	+	**9.5**	**81.0**	**90.5**
**ATR**					+	+		+	+	+	+	++	++	+	++	+		+	+	+	+	**14.3**	**57.1**	**71.4**
**PRKDC**	+	+	+	+	+	+	+	+	+	+	+	+	++	+	+	+	+	+	+	+	+	**4.8**	**95.2**	**100.0**
**CHEK1**		+	+	+	+	+	+	+	+	+	+	+	++	+	+	+		+	+	+	+	**4.8**	**85.7**	**90.5**
**CHEK2**			++	++	+	++		+	+	+	+		++	+		+	+		++	+		**23.8**	**42.9**	**66.7**
**H(%)***	**3.8**	**0.0**	**3.8**	**7.7**	**0.0**	**3.8**	**3.8**	**0.0**	**0.0**	**0.0**	**0.0**	**23.1**	**73.1**	**7.7**	**7.7**	**3.8**	**0.0**	**0.0**	**19.2**	**0.0**	**3.8**			
**L(%)***	**23.1**	**61.5**	**84.6**	**76.9**	**96.2**	**92.3**	**61.5**	**100.0**	**92.3**	**96.2**	**96.2**	**69.2**	**19.2**	**84.6**	**76.9**	**92.3**	**73.1**	**73.1**	**80.8**	**88.5**	**57.7**			
**H+L(%)***	**26.9**	**61.5**	**88.5**	**84.6**	**96.2**	**96.2**	**65.4**	**100.0**	**92.3**	**96.2**	**96.2**	**92.3**	**92.3**	**92.3**	**84.6**	**96.2**	**73.1**	**73.1**	**100.0**	**88.5**	**61.5**			

++: High expression, +Low expression and empty box: No expression, H: High expression rate; L: Low expression rate. Three levels of gene expression can be identified.

Supplementary table 3. Among 26 DNA damage checkpoint factors, two factors including Parp1 and Rpa1are broadly expressed▲, and others are differentially expressed in mouse tissues.

	Mouse Tissues (Number: 20)																						
**Gene**	**Adipose tissue**	**Adrenal gland**	**Blood**	**Bone marrow**	**Brain**	**Embryonic tissue**	**Heart**	**Intestine**	**Kidney**	**Liver**	**Lung**	**Lymph node**	**Muscle**	**Ovary**	**Pancreas**	**Skin**	**Spleen**	**Stomach**	**Testis**	**Thymus**	**H(%)**	**L(%)**	**H+L(%)**
**Rad9a**				+	+	+		+	+	+	++	++		+	++	+	+	+	+	+	**15.0**	**60.0**	**75.0**
**Rad1**				+	+	+	+		+		+		++	+	++		+		+	+	**10.0**	**50.0**	**60.0**
**Hus1**				+	+	+	+		+	+	+			++		+	+		+	+	**5.0**	**55.0**	**60.0**
**Mre11a**			++	+	+	+	+	+			+			+	+	+	++		+	++	**15.0**	**50.0**	**65.0**
**Rad50**			++	+	+	+	+				+	++		++	++	+		+	+	++	**25.0**	**40.0**	**65.0**
**Nbn**				+	+	+		+	+	+	+		++	+	+	+	+		++	+	**10.0**	**60.0**	**70.0**
**Rpa1**			+	+	+	+	+	+	+	+	+		+	++	+	+	+	+	++	++	**15.0**	**70.0**	**85.0**
**Rpa2**				+	+	+	+		+	+	+	++	+	+	+	+			+	+	**5.0**	**65.0**	**70.0**
**Rpa3**				+	+	+	+	++			++	++		++			+		++	++	**30.0**	**25.0**	**55.0**
**Atrip**				+	++	+			+	+	+		++	+	++	++	+			+	**20.0**	**40.0**	**60.0**
**Rad17**			++	+	+	+			+	+	+			++	++	+	++	++	+	+	**25.0**	**45.0**	**70.0**
**Parp1**		++	+	+	+	+	+	+	+	+	+	++		+	+	+	+	+	+	+	**10.0**	**80.0**	**90.0**
**Parp2**			+	+	+	+	+	+	+			++		+	++	+	+	+		++	**15.0**	**55.0**	**70.0**
**Xrcc6**			+	+	+	+		+	+	+	+	++	+	+	++	+	+		+	++	**15.0**	**65.0**	**80.0**
**Xrcc5**			+	+	+	+	+	+	+	+	+	++		+		+	+		+	++	**10.0**	**65.0**	**75.0**
**Il1a**				+												+	++				**5.0**	**10.0**	**15.0**
**Brca1**					+	++			+		+	++		++	++	++	++			++	**35.0**	**15.0**	**50.0**
**Topbp1**				+	+	+	+		+		+	++		+	+	+	+		+	+	**5.0**	**60.0**	**65.0**
**Trp53bp1**				+	++	+	+	+	+		+	++		++	++	+	+		++	+	**25.0**	**45.0**	**70.0**
**Mdc1**				+	+	+	+	+	+	+	+	++		+	++	+	+		+	+	**10.0**	**65.0**	**75.0**
**H2afx**					+	+	+	+		+	+			++	++		+	++	+	++	**20.0**	**40.0**	**60.0**
**Atm**				+	+	+		+	+	+	+	++	++	+	++		++		+	+	**20.0**	**50.0**	**70.0**
**Atr**				+	+	+	+			+	+			++	++	++	++		++	++	**30.0**	**30.0**	**60.0**
**Prkdc**				+	+	+	+					++				+				+	**5.0**	**30.0**	**35.0**
**Chek1**			+	+	+	+		+	+	+	+	++		++		+			++	+	**15.0**	**50.0**	**65.0**
**Chek2**				+	+	++	++		+		++		++	++		++	++			++	**40.0**	**15.0**	**55.0**
**H(%)***	**0.0**	**3.8**	**11.5**	**0.0**	**7.7**	**7.7**	**3.8**	**3.8**	**0.0**	**0.0**	**11.5**	**57.7**	**19.2**	**42.3**	**50.0**	**15.4**	**26.9**	**7.7**	**23.1**	**42.3**			
**L(%)***	**0.0**	**0.0**	**23.1**	**92.3**	**88.5**	**88.5**	**61.5**	**50.0**	**73.1**	**57.7**	**76.9**	**0.0**	**11.5**	**50.0**	**23.1**	**69.2**	**57.7**	**19.2**	**53.8**	**53.8**			
**H+L(%)***	**0.0**	**3.8**	**34.6**	**92.3**	**96.2**	**96.2**	**65.4**	**53.8**	**73.1**	**57.7**	**88.5**	**57.7**	**30.8**	**92.3**	**73.1**	**84.6**	**84.6**	**26.9**	**76.9**	**96.2**			

++:High expression, +Low expression and empty box: No expression , H: High expression rate; L: Low expression rate. Three levels of gene expression can be identified.

▲: The Gene is expressed in tissues [H+L(%)>85%]

H+L(%) *⩾75%: Tissues express most of checkpoint factors respectively.

Supplementary table 4. 42 human DNA Damage repair factors classified in five subgroups are focused in this study. *

		Gene name (Full name)	Gene Symbol	Function	UniGene ID Human (Hs.)	UniGene ID Mouse (Mm.)	PMID
1	MGMT	O6-methylguanine-DNA methyltransferase	MGMT	Direct reversal （DR）	501522	440219	21211780
2	ALKBH2	AlkB, alkylation repair homolog 2	ALKBH2		374458	332593	16498454
3	ALKBH3	AlkB, alkylation repair homolog 3	ALKBH3		720708	272498	16314342
4	OGG1	8-oxoguanine DNA glycosylase	OGG1	Base excision repair (BER)	380271	43612	27534801/ 28347738
5	NEIL1	Nei endonuclease VIII-like 1	NEIL1		512732	35749	23684800
6	MUTYH	mutY homolog	MUTYH		271353	180333	23684800
7	UNG	Uracil-DNA glycosylase	UNG		191334	1393	23684800
8	SMUG1	Single-strand-selective monofunctional uracil-DNA glycosylase 1	SMUG1		632721	254820	23684800
9	MPG	N-methylpurine-DNA glycosylase	MPG		459596	26316	23684800
10	APEX1	APEX nuclease (multifunctional DNA repair enzyme) 1	APEX1		73722	203	27534801/28347738
11	APEX2	APEX nuclease (apurinic/apyrimidinic endonuclease) 2	APEX2		659558	440275	27534801/28347738
12	POLβ	Polymerase (DNA directed), beta	POLB		654484	123211	23684800
13	PNKP	Polynucleotide kinase 3'-phosphatase	PNKP		78016	238254	23684800
14	XPC	Xeroderma pigmentosum, complementation group C	XPC	Nucleotide excision repair(NER)	475538	2806	23684800
15	RAD23B	RAD23 homolog B	RAD23B		521640	196846	23684800
16	XPA	Xeroderma pigmentosum, complementation group A	XPA		654364	247036	23684800
17	XPD	Excision repair cross-complementing rodent repair deficiency, complementation group 2	ERCC2		487294	36524	23684800
18	XPB	Excision repair cross-complementing rodent repair deficiency, complementation group 3	ERCC3		469872	282335	23684800
19	ERCC1	Excision repair cross-complementing rodent repair deficiency, complementation group 1	ERCC1		435981	280913	23684800
20	XPG	Excision repair cross-complementing rodent repair deficiency, complementation group 5	ERCC5		258429	2213	23684800
21	XPF	Excision repair cross-complementing rodent repair deficiency, complementation group 4	ERCC4		567265	287837	23684800
22	LIG1	Ligase I, DNA, ATP-dependent	LIG1		1770	288179	23684800
23	CSA	Excision repair cross-complementing rodent repair deficiency, complementation group 8	ERCC8		435237	212208	23684800
24	CSB	Excision repair cross-complementing rodent repair deficiency, complementation group 6	ERCC6		49063	318310	23684800
25	XRCC2	X-ray repair complementing defective repair in Chinese hamster cells 2	XRCC2	**DSBR** -Homologous recombination repair (HRR)	647093	143767	23684800 28143930 27534801
26	XRCC3	X-ray repair complementing defective repair in Chinese hamster cells 3	XRCC3		592325	19082	
27	BRCA2	Breast cancer 2	BRCA2		34012	236256	
28	RAD52	RAD52 homolog	RAD52		410355	149	
29	RAD51	RAD51 homolog	RAD51		631709	330492	
30	LIG4	Ligase IV, DNA, ATP-dependent	LIG4	**DSBR** - Nonhomologous end joining (NHEJ)	166091	80584	28143930
31	XLF	Nonhomologous end-joining factor 1	NHEJ1		225988	442409	16439205
32	XRCC4	X-ray repair complementing defective repair in Chinese hamster cells 4	XRCC4		567359	37531	16439205
33	XRCC1	X-ray repair complementing defective repair in Chinese hamster cells 1	XRCC1	**DSBR** - Microhomologymediated end-joining (alt-NHEJ)	98493	4347	23684800 /28143930
34	LIG3	Ligase III, DNA, ATP-dependent	LIG3		100299	277136	23684800
35	MLH1	MutL homolog 1	MLH1	Mismatch repair (MMR)	195364	486383	27534801 28187216 28067827
36	MLH3	MutL homolog 3	MLH3		436650	311981	
37	MSH2	MutS homolog 2	MSH2		597656	4619	
38	MSH3	MutS homolog 3	MSH3		280987	343101	
39	MSH6	MutS homolog 6	MSH6		445052	18210	
40	PMS1	Postmeiotic segregation increased 1	PMS1		111749	60499	
41	PMS2	Postmeiotic segregation increased 2	PMS2		632637	2950	
42	HMGB1	High mobility group box 1	HMGB1	NER,BER,MMR	434102	207047	25449753/ 26916160

* This list represents the major genes in DNA repair pathway, shown in Figure3.

Supplementary table 5. Among 42 DNA damage repair factors, six factors including APEX1, XPC, ERCC3, ERCC5, HMGB1 and MLH1 are ubiquitously expressed, and others are differentially expressed in human tissues.

	Human Tissues (Number: 21)																							
**Gene**	**Adipose tissue**	**Adrenal gland**	**Blood**	**Bone marrow**	**Brain**	**Embryonic tissue**	**Heart**	**Intestine**	**Kidney**	**Liver**	**Lung**	**Lymph node**	**Muscle**	**Ovary**	**Pancreas**	**Skin**	**Spleen**	**Stomach**	**Testis**	**Thymus**	**Vascular**	**H(%)**	**L(%)**	**H+L(%)**
**MGMT**			+		+	+	+	+	+	+	+		++	++	+	+	+	+	+		+	**9.5**	**66.7**	**76.2**
**ALKBH2**	+	+	+	+	+	+	+	+	+	+	+	+		+	+	+		+	+		+	**0.0**	**85.7**	**85.7**
**ALKBH3**			+		+	+	+	+	+	+	+		++	+	+	+	+		++	+	+	**9.5**	**66.7**	**76.2**
**OGG1**	++		+	+	+	+		+	++	+	+	++		+	+	+		++	+	+		**19.0**	**57.1**	**76.2**
**NEIL1**		+	+	+	+	+	+	+	+	+	+	++	+	+	+		+	+	+	++		**9.5**	**76.2**	**85.7**
**MUTYH**	+		+	+	+	+	+	+	+	+	+	+		+	+	+		+	+	+		**0.0**	**81.0**	**81.0**
**UNG**		++	+	+	+	+	+	+	+	+	+	+	+	+	+	+	+	+	+	+	+	**4.8**	**90.5**	**95.2**
**SMUG1**			+		+	+	+	+	+	+	+	+	++	++		+			+			**9.5**	**52.4**	**61.9**
**MPG**	+			++	+	+	+	+	+	+	+	+	++	+	+	+	+	+	+			**9.5**	**71.4**	**81.0**
**APEX1**	+	+	+	+	+	+	+	+	+	+	+	+	+	+	+	+	+	+	+	+	+	**0.0**	**100.0**	**100.0**
**APEX2**		+	+		+	+		+	+	+	+		++	++	+	+		+	+		+	**9.5**	**61.9**	**71.4**
**POLB**			+	+	+	+	+	+	+	+	+	+	++	+	+	+	+	+	+	+	+	**4.8**	**85.7**	**90.5**
**PNKP**			+	+	+	+	+	+	+	+	+	+		+	+	+		+	+			**0.0**	**71.4**	**71.4**
**XPC**	++	+	+	+	+	+	+	+	+	+	+	+	++	+	+	+	+	+	+	+	+	**9.5**	**90.5**	**100.0**
**RAD23B**	+	+	+	+	+	+	+	+	+	+	++	+	++	+	+	+		+	+	+	+	**9.5**	**85.7**	**95.2**
**XPA**			+		+	+	++	+	+	+	++			++	++	+					+	**19.0**	**38.1**	**57.1**
**ERCC2**			+	+	+	+	+	+		+	+	+	++	+	+	+	+	+	+	+		**4.8**	**76.2**	**81.0**
**ERCC3**	+	+	+	+	+	+	+	+	+	+	+	+	+	+	+	+	+	+	++	+	+	**4.8**	**95.2**	**100.0**
**ERCC1**		+	+	+	+	+	+	+	+	+	+	+	+	+	+	+		+	+		+	**0.0**	**85.7**	**85.7**
**ERCC5**	+		+	+	+	+	+	+	+	+	+	+	+	+	+	+	+	+	+	+	+	**4.8**	**95.2**	**100.0**
**ERCC4**				+	+	+			+	+	+	+	++	+			+		++	+		**9.5**	**47.6**	**57.1**
**LIG1**	+		+	+	+	+	+	+	+	+	+	+	++	+	+	+	+	+	+	+	+	**4.8**	**90.5**	**95.2**
**ERCC8**			+	++	+	+	+	+	+	+	+		++	+	+	+		+	++	+	+	**14.3**	**66.7**	**81.0**
**ERCC6**			+		+	+	+	+	++	++	+	+	++			+		+	++		++	**23.8**	**42.9**	**66.7**
**XRCC2**					+	+				+		+							+			**0.0**	**23.8**	**23.8**
**XRCC3**			+									+										**0.0**	**9.5**	**9.5**
**BRCA2**				++	+	++		++	++	++	++	++			++				++		++	**47.6**	**4.8**	**52.4**
**RAD52**		+	+	+	+	+	+	+	+		+	+				+	+		++	+		**4.8**	**61.9**	**66.7**
**RAD51**		+	+	++	+	+	+	+	+	+	+		++	+	+	+		+	++	+		**14.3**	**66.7**	**81.0**
**LIG4**			+		+			+	+	++		++	++		+	+	+	+	++	++	+	**23.8**	**42.9**	**66.7**
**NHEJ1**			+	+	+	+		+	+	++	+	+		+		+	+	+	++	+	+	**9.5**	**66.7**	**76.2**
**XRCC4**		+	+	++	+	+		+	+	+		+			+	+		+	++		+	**9.5**	**57.1**	**66.7**
**XRCC1**			+	+	+	+	+	+	+	+	+	+	++	+	+	+			+			**4.8**	**66.7**	**71.4**
**LIG3**	+			++	+	+		+	+	+	+	+	++	+	+	+	+	+	++	+		**14.3**	**66.7**	**81.0**
**MLH1**	+	+	+	+	+	+	+	+	+	+	+	+	++	+	+	+	+	+	++	+	+	**9.5**	**90.5**	**100.0**
**MLH3**		+		++	+	+	+	+	+	+	+	+	+		+	+		+	++	+	+	**9.5**	**71.4**	**81.0**
**MSH2**		+	+	+	+	+	+	+	+	+	+	+	+	+	+	+	+	+	++	+	+	**4.8**	**90.5**	**95.2**
**MSH3**		+		+	+	+		+	++	+	+	++	++	+	+	+		+	+		+	**14.3**	**61.9**	**76.2**
**MSH6**		+	+	+	+	+	+	+	+	+	+	+	+	+	+	+	+	+	++	+		**4.8**	**85.7**	**90.5**
**PMS1**	+		+	+	+	+	+	+	+	+	+	+	+	+	+	+		+	++	+		**4.8**	**81.0**	**85.7**
**PMS2**		+		++	+	+	+	+	+	+	+	+	++	+	+	+			++	+	+	**14.3**	**66.7**	**81.0**
**HMGB1**	+	+	+	+	+	+	+	+	+	+	+	+	++	+	+	+	+	+	+	+	+	**4.8**	**95.2**	**100.0**
**H(%)***	**4.8**	**2.4**	**0.0**	**19.0**	**0.0**	**2.4**	**2.4**	**2.4**	**9.5**	**7.1**	**7.1**	**11.9**	**50.0**	**9.5**	**4.8**	**0.0**	**0.0**	**2.4**	**42.9**	**4.8**	**4.8**			
**L(%)***	**28.6**	**42.9**	**81.0**	**59.5**	**97.6**	**92.9**	**71.4**	**90.5**	**83.3**	**88.1**	**83.3**	**73.8**	**23.8**	**71.4**	**78.6**	**88.1**	**50.0**	**73.8**	**52.4**	**59.5**	**57.1**			
**H+L(%)***	**33.3**	**45.2**	**81.0**	**78.6**	**97.6**	**95.2**	**73.8**	**92.9**	**92.9**	**95.2**	**90.5**	**85.7**	**73.8**	**81.0**	**83.3**	**88.1**	**50.0**	**76.2**	**95.2**	**64.3**	**61.9**			

++: High expression, +Low expression and empty box: No expression, H: High expression rate; L: Low expression rate. Three levels of gene expression can be identified.

Supplementary table 6. Among 42 DNA damage repair factors, five factors including Rad23b, Hmgb1, Xrcc1, Lig3 and Mlh1are ubiquitously expressed▲.

Mouse Tissues (Number: 20)

Gene	Adipose tissue	Adrenal gland	Blood	Bone marrow	Brain	embryonic tissue	Heart	Intestine	kidney	liver	Lung	lymph node	Muscle	ovary	Pancreas	Skin	Spleen	Stomach	Testis	Thymus	H(%)	L(%)	H+L(%)
Mgmt						+			+	++									+		5.0	15.0	20.0
Alkbh2					+	+				+	+			+		++					5.0	25.0	30.0
Alkbh3			++	+	+	+	+		+	+	+		+		++	+		+	++	+	15.0	55.0	70.0
Ogg1				+	+	+	++	+	+		+			++	++	+		++	+	+	20.0	45.0	65.0
Neil1					+	+		+			+		++	+		+				+	5.0	35.0	40.0
Mutyh				+	+	+	+	+		+				+			+			+	0.0	45.0	45.0
Ung				+	+	+	++	++	+	+	+		++	++			+		++	++	30.0	35.0	65.0
Smug1					+	+				+	+			+		+	++			+	5.0	35.0	40.0
Mpg					+	+	+	+	+		+	++	+	+	+	+	+	+	+	+	5.0	70.0	75.0
Apex1				+	+	+	+	+	+	+	+		+	+	+	+	+		+	+	0.0	75.0	75.0
Apex2					+	+		+	+		+			+	+		+			+	0.0	45.0	45.0
Polb				+	++	+		++	+			++	++	+	++	++			++		35.0	20.0	55.0
Pnkp				+	+	+	+	+	+	+	+		+		+	+	++	+	+	++	10.0	65.0	75.0
Xpc			++	+	+	+			+	+	+	++	+	+	+	+			+	+	10.0	65.0	75.0
Rad23b		++	+	+	+	+	+	+	+	+	+	++		+	+	+	+	+	+	+	10.0	80.0	90.0
Xpa					+	+			+		+				+	+			+	+	0.0	40.0	40.0
Ercc2					+	+	+	+	+	+	+		++	+	+	+	++		+	+	10.0	60.0	70.0
Ercc3				+	+	+	+	+	+	+	+			+	+	++	+		+	+	5.0	65.0	70.0
Ercc1				+	++	+	++		+	+	++		++		+			+	+	++	25.0	35.0	60.0
Ercc5				+	++	+			+	+			++	++	++	+	+		+	+	20.0	40.0	60.0
Ercc4			++	+	+	+	++	+	+		+		++		++	+				+	20.0	40.0	60.0
Lig1			+	+	+	+	+	+	+	+	+		+		+	+	+		+	++	5.0	70.0	75.0
Ercc8				+	+	+	+	+	+		+	++	++			+	++		+	+	15.0	50.0	65.0
Ercc6				+	+	+	+		+	+			+		+	+	+		+	+	0.0	60.0	60.0
Xrcc2				+	++	++			++					++	++	++	++		++	++	45.0	5.0	50.0
Xrcc3					+	+	+	+	+	++			++		+	+			+	+	10.0	45.0	55.0
Brca2			++	+	+	++			+		+		++	++	++	+			++	+	30.0	30.0	60.0
Rad52			++	+	+	+	+	+		+	+			+		+	+	++	+	++	15.0	55.0	70.0
Rad51				+	+	+	+	+	+	+	+	++		++	++	+	+		+	+	15.0	60.0	75.0
Lig4				+	+	+						++				+	+	+	+	+	5.0	40.0	45.0
Nhej1					+	+	++				+				+				+	+	5.0	30.0	35.0
Xrcc4					+	+			+	+			++	++					+	+	10.0	30.0	40.0
Xrcc1				+	+	+	+	+	+	+	+	++	+	+	+	+	+	+	+	+	5.0	80.0	85.0
Lig3	+		+	+	+	+		+	+	+	+		+	+	+	+	++	+	+	+	5.0	80.0	85.0
Mlh1		++	+	+	+	+	+		+	+	+	++	+	+	+	+	+		+	+	10.0	75.0	85.0
Mlh3			++	+	++	++	++		++					++			++		++	++	45.0	5.0	50.0
Msh2			+	+	+	+		+	+	+	+	++	+	+	+	+	+		+	+	5.0	75.0	80.0
Msh3				+	++	++					+	++		++	++	++	++		++	++	45.0	10.0	55.0
Msh6			+		+	++			+	+	+			++	++	+	+		+	++	20.0	40.0	60.0
Pms1					++	++	++		+	++				++	++	++			++	+	40.0	10.0	50.0
Pms2				+	+	+		+	+		+	++		+	+	+	+			+	5.0	55.0	60.0
Hmgb1				+	+	+	+	+	+	+	+	++	+	+	+	+	+	+	+	+	5.0	80.0	85.0
H(%)*	0.0	4.8	14.3	0.0	16.7	14.3	16.7	4.8	4.8	7.1	2.4	31.0	26.2	26.2	26.2	14.3	19.0	4.8	19.0	21.4			
L(%)*	2.4	0.0	14.3	69.0	81.0	85.7	40.5	50.0	76.2	57.1	71.4	0.0	28.6	45.2	47.6	66.7	45.2	21.4	64.3	71.4			
H+L(%)*	2.4	4.8	28.6	69.0	97.6	100.0	57.1	54.8	81.0	64.3	73.8	31.0	54.8	71.4	73.8	81.0	64.3	26.2	83.3	92.9			

++:High expression, +Low expression and empty box: No expression. H: High expression rate; L: Low expression rate. Three levels of gene expression can be identified,

▲: The Gene is expressed in tissues [H+L(%)>85%],

H+L(%) *⩾75%: Tissues express most of repair factors respectively.

Supplementary table 7. The gene expression changes of DNA damage checkpoint factors are focused in vascular diseases. Human coronary artery disease induce upregulation of some DNA damage checkpoint factors.

	Vascular diseases			
**GEO ID**	GSE57691	GSE9874	GSE23561	GSE19339
**Tissue**	Human aortic	Human monocyte-derived macrophages (Peripheral blood)	Human peripheral blood	Human leukocytes
**Comparison**	Aortic occlusive disease vs. healthy controls	Atherosclerosis vs. healthy controls	Coronary artery disease vs. healthy controls	Acute coronary syndrome patients vs. healthy controls
RAD9A				
RAD1			2.75	
HUS1				
MRE11A			2.19	
RAD50				0.28
NBN			2.19	0.40
RPA1				
RPA2				
RPA3				
ATRIP				
RAD17			2.06	
PARP1				
PARP2				
XRCC6				
XRCC5				0.50
IL1A				2.63
BRCA1				
TOPBP1				
TP53BP1				
MDC1				
H2AFX				
ATM				
ATR				
PRKDC		0.49		
CHEK1			2.16	
CHEK2			2.28	
**Up**	0	0	6/26	1/26
**Down**	0	1/26	0	3/26

- Genes with significant expression changes ($P < 0.05$) are shown here. Red text stands for upregulation ($FC \geq 2$), green text stands for downregulation ($FC \leq 0.5$).

Supplementary table 8. The gene expression changes of DNA damage repair factors are focused in vascular diseases. 1) Human coronary artery disease and atherosclerosis induced the upregulation of some DNA damage repair factors; 2) Human leukocytes from acute coronary syndrome patients induce downregulation of few DNA damage repair factors.

	Vascular diseases			
**GEO ID**	GSE57691	GSE9874	GSE23561	GSE19339
**Tissue**	Human aortic	Human monocyte-derived macrophages (Peripheral blood)	Human peripheral blood	Human leukocytes
**Comparison**	Aortic occlusive disease vs. Healthy controls	Atherosclerosis vs. Healthy controls	Coronary artery disease vs. healthy controls	Acute coronary syndrome patients vs. Healthy controls
MGMT				
ALKBH2				
ALKBH3				
OGG1				
NEIL1				
MUTYH				
UNG				
SMUG1				
MPG			2.02	
APEX1				
APEX2				
POLB				
PNKP				
XPC				
RAD23B				
XPA			2.4	
ERCC2				
ERCC3				
ERCC1			2.02	0.43
ERCC5				
ERCC4				
LIG1				
ERCC8				
ERCC6			2.15	
XRCC2			2.34	
XRCC3				
BRCA2		2.1	2.03	
RAD52		2.89	2.32	0.35
RAD51			2.33	
LIG4			2.03	
NHEJ1				
XRCC4				
XRCC1				
LIG3				0.29
MLH1				
MLH3			2.5	
MSH2				
MSH3	2.55		2.3	
MSH6	0.44			
PMS1				
PMS2			2.02	
HMGB1	0.46			
**Up**	1/42	2/42	12/42	0
**Down**	2/42	0	0	3/42

- Genes with significant expression changes ($P < 0.05$) are shown here. Red text stands for upregulation ($FC \geq 2$), green text stands for downregulation ($FC \leq 0.5$).

Supplementary table 9. The gene expression changes of DNA damage checkpoint factors are focused in autoimmune diseases. Human rheumatoid arthritis and osteoarthritis induced upregulation of a few DNA damage checkpoint factors.

	Autoimmune diseases				
**GEO ID**	GSE55235	GSE55235	GSE81622	GSE57376	GSE27335
**Tissue**	Human synovial membrane	Human synovial membrane	Human PBMCs	Human skin	Human airway fibroblasts
**Comparison**	Rheumatoid arthritis vs. healthy controls	Osteoarthritis vs. healthy Controls	Systemic lupus erythematosus vs. healthy controls	Lesional skin vs. nonlesional skin in psoriasis patients	Asthma patient vs. healthy Controls
RAD9A					
RAD1					
HUS1					
MRE11A					
RAD50					
NBN	2.31	2.03			
RPA1					
RPA2					
RPA3					
ATRIP		2.18			
RAD17	2.27				
PARP1					
PARP2					
XRCC6					
XRCC5					
IL1A					
BRCA1	2.69	2.20			
TOPBP1					
TP53BP1					
MDC1					
H2AFX					
ATM					
ATR					
PRKDC					
CHEK1				2.19	
CHEK2					
**Up**	3/26	3/26	0	1/26	0
**Down**	0	0	0	0	0

- Genes with significant expression changes ($P < 0.05$) are shown here. Red text stands for upregulation ($FC \geq 2$). FC: fold change.

Supplementary table 10. The gene expression changes of DNA damage repair factors are focused in vascular diseases and autoimmune diseases. The autoimmune diseases have limited effects on the expression of DNA damage repair factors (4/42).

	**Autoimmune diseases**				
**GEO ID**	GSE55235	GSE55235	GSE81622	GSE57376	GSE27335
**Tissue**	Human Synovial membrane	Human Synovial membrane	Human PBMCs	Human skin	Human airway fibroblasts
**Comparison**	Rheumatoid arthritis vs. healthy controls	Osteoarthritis vs. healthy controls	Systemic lupus erythematosus vs. healthy controls	Lesional skin vs. nonlesional skin in psoriasis patients	Asthma patient vs. healthy controls
MGMT					
ALKBH2					
ALKBH3					
OGG1					
NEIL1					
MUTYH					
UNG					
SMUG1					
MPG					
APEX1					
APEX2					
POLB					
PNKP					
XPC					
RAD23B		0.35			
XPA					
ERCC2					
ERCC3					
ERCC1					
ERCC5					
ERCC4					
LIG1					
ERCC8					
ERCC6					
XRCC2					
XRCC3					
BRCA2					2.37
RAD52					
RAD51	2.03				
LIG4					
NHEJ1					
XRCC4				2.08	
XRCC1					
LIG3					
MLH1					
MLH3					
MSH2					
MSH3					
MSH6					
PMS1					
PMS2					
HMGB1					
**Up**	1/42	0	0	1/42	1/42
**Down**	0	1/42	0	0	0

- Genes with significant expression changes ($P < 0.05$) are shown here. Red text stands for upregulation ($FC \geq 2$), green text stands for downregulation ($FC \leq 0.5$). FC: fold change.

Supplementary table 11. The gene expression changes of DNA damage checkpoint factors are in digestive diseases. Human ulcerative colitis, Crohn's colitis and Crohn's ileitis induced upregulation of a few DNA damage checkpoint factors.

**Digestive diseases**					
**GEO ID**	GSE16879			GSE27411	GSE27411
**Tissue**	Human colonic mucosa	Human colonic mucosa	Human ileal mucosa	Human gastric corpus	Human gastric corpus
**Comparison**	Ulcerative colitis vs. healthy controls	Crohn's colitis vs. healthy controls	Crohn's ileitis vs. healthy controls	HP-Infection with corpus-predominant atrophic gastritis vs. healthy controls	Atrophic gastritis vs. healthy controls
RAD9A					
RAD1					
HUS1					
MRE11A		2.2			
RAD50					
NBN					
RPA1					
RPA2					
RPA3					
ATRIP					
RAD17					
PARP1					
PARP2					
XRCC6					
XRCC5					
IL1A	10.8	8.41	5.74		
BRCA1					
TOPBP1					
TP53BP1					
MDC1					
H2AFX		2.58			
ATM					
ATR					
PRKDC			3.61		
CHEK1	2.20	2.31		2.80	2.30
CHEK2					
**Up**	2/26	4/26	2/26	1/26	1/26
**Down**	0	0	0	0	0

- Genes with significant expression changes ($P < 0.05$) are shown here. Red text stands for upregulation (FC ≥2). FC: fold change

Supplementary table 12. The gene expression changes of DNA damage repair factors are focused in digestive diseases. Human ulcerative colitis and crohn's colitis induced upregulation of a few DNA damage repair factors.

	**Digestive diseases**				
**GEO ID**	GSE16879			GSE27411	GSE27411
**Tissue**	Human colonic mucosa	Human colonic mucosa	Human ileal mucosa	Human gastric corpus	Human gastric corpus
**Comparison**	Ulcerative colitis vs. healthy controls	Crohn's colitis vs. healthy controls	Crohn's ileitis vs. health y controls	HP-Infection with corpus-predominant atrophic gastritis vs. healthy controls	Atrophic gastritis vs. healthy controls
MGMT					
ALKBH2					
ALKBH3					
OGG1					0.39
NEIL1					
MUTYH					
UNG					
SMUG1					
MPG					
APEX1					
APEX2					
POLB					
PNKP					
XPC					
RAD23B			2.2		
XPA					
ERCC2					
ERCC3					
ERCC1					
ERCC5					
ERCC4					
LIG1	2.46	2.48			
ERCC8					
ERCC6					
XRCC2					
XRCC3					
BRCA2					
RAD52	0.43				
RAD51				2.29	2.36
LIG4					
NHEJ1					
XRCC4					2.92
XRCC1					
LIG3		2.16			
MLH1					
MLH3					
MSH2					
MSH3					
MSH6					
PMS1					
PMS2					
HMGB1					
**Up**	1/42	2/42	1/42	1/42	2/42
**Down**	1/42	0	0	0	1/42

- Genes with significant expression changes ($P < 0.05$) are shown here. Red text stands for upregulation ($FC \geq 2$), green text stands for downregulation ($FC \leq 0.5$). FC: fold change.

Supplementary table 13. The gene expression changes of DNA damage checkpoint factors are focused in metabolic diseases. Human pancreas islets of Type 2 diabetes induced downregulation of a few DNA damage checkpoint factors, and the human embryonic kidney cell treated with high glucose induced upregulation of a few DNA damage checkpoint factors.

	**Metabolic diseases**							**Metabolin treatment**			
GEO ID	GSE 30528	GSE 25724	GSE 23561	GSE 55100	GSE 6088	GSE 43760	GSE 60436	GSE 46262	GSE 15575	GSE 9490	GSE 13139
Tissue	Human glomeruli	Human pancreas islets	Human peripheral blood	Human PBMCs	Human T cell	Human vastus lateralis muscle	Human Fibrovascular membranes	Human vastus CD34+ progenitor endothelial cells	Human embryonic kidney cell	Human aortic smooth muscle cells	Human aortic endothelial cells
Comparion	Diabetic Kidney Disease vs. Healthy control	Type 2 diabetes vs. healthy control	Type 2 diabetes vs. healthy controls	Type 1 diabetes vs. healthy controls	Homozygous familial hypercholestero-lemia patients vs. health control	Metabolic syndrome vs. healthy control	Proliferative diabetic retinopathy vs. healthy Controls	High glucose (25mM)	High glucose (450mg/dl)	Homocysteine (100 μmol/L)	Oxidized LDL (12h)
RAD9A											
RAD1											
HUS1											
MRE11A					0.12						
RAD50											
NBN									2.84		
RPA1											
RPA2											
RPA3											
ATRIP											
RAD17		0.45									
PARP1											
PARP2											
XRCC6											
XRCC5		0.29									
IL1A								0.17	3.05		
BRCA1								2.01			
TopBP1							2.53				
TP53BP1											
MDC1											
H2AFX											
ATM											
ATR		0.50					0.37		2.48		
PRKDC											8.92
CHEK1							3.03				
CHEK2											
**Up**	0	0	0	0	0	0	2/26	1/26	3/26	0	1/26
**Down**	0	3/26	0	0	1/26	0	1/26	1/26	0	0	0

- Genes with significant expression changes ($P < 0.05$) are shown here. Red text stands for upregulation ($FC \geq 2$), green text stands for downregulation ($FC \leq 0.5$).

Supplementary table 14. The gene expression changes of DNA damage repair factors are in metabolic diseases. 1) Human pancreas islets of Type 2 diabetes induced downregulation of a few DNA damage repair factors; 2) Human peripheral blood of Type 2 diabetes and human aortic endothelial cells treated with oxidized LDL induced upregulation of a few DNA damage repair factors.

	**Metabolic diseases**							**Metabolin treatment**			
GEO ID	GSE 30528	GSE 25724	GSE 23561	GSE 55100	GSE 6088	GSE 43760	GSE 60436	GSE 46262	GSE 15575	GSE 9490	GSE 13139
Tissue	Human glomeruli	Human pancreas islets	Human peripheral blood	Human PBMCs	Human T cell	Human vastus lateralis muscle	Human Fibrovascular membranes	Human vastus CD34+ progenitor endothelial cells	Human embryonic kidney cell	Human aortic smooth muscle cells	Human aortic endothelia l cells
Comparion	Diabetic Kidney Disease vs. Healthy control	Type 2 diabetes vs. healthy control	Type 2 diabetes vs. healthy controls	Type 1 diabetes vs. healthy controls	Homozygous familial hypercholestero -lemia patients vs. health control	Metabolic syndrome vs. healthy control	Proliferative diabetic retinopathy vs. healthy Controls	High glucose (25mM)	High glucose (450mg/dl)	Homocysteine (100 μmol/L)	Oxidized LDL (12h)
MGMT											
ALKBH2											
ALKBH3											
OGG1									0.29		2.2
NEIL1											
MUTYH											
UNG							0.34				
SMUG1									0.44		
MPG											
APEX1		0.38									
APEX2											
POLB											
PNKP									0.48		
XPC											
RAD23B		0.44							2.29		
XPA											
ERCC2											
ERCC3											
ERCC1									0.27		
ERCC5							2.26				
ERCC4											
LIG1											
ERCC8											4.22
ERCC6							2.03				
XRCC2											
XRCC3											
BRCA2											
RAD52											
RAD51			2.25								
LIG4											
NHEJ1											
XRCC4											
XRCC1											
LIG3											
MLH1		0.48									
MLH3											
MSH2											
MSH3					0.31				3.51		
MSH6		0.47									
PMS1											
PMS2			2.23								
HMGB1											
**Up**	0	0	2/42	0	0	0	2/42	0	2/42	0	2/42
**Down**	0	4/42	0	0	1/42	0	1/42	0	4/42	0	0

- Genes with significant expression changes ($P < 0.05$) are shown here. Red text stands for upregulation ($FC \geq 2$), green text stands for downregulation ($FC \leq 0.5$).

Supplementary table 15. The gene expression changes of DNA damage checkpoint factors are analyzed in aging and ageing diseases. Human Hutchinson-Gilford progeria syndrome, old sepsis induced multiple organ failure, and the spleen of old mouse induced upregulation of a few DNA damage checkpoint factors.

**GEO ID**	GSE41751	GSE1297	GSE1297	GSE1297	GSE32614	GSE32614	GSE13205	GSE3253	GSE34378
**Tissue**	Human primary fibroblasts from skin	Human hippocampus	Human hippocampus	Human hippocampus	Human macular retina	Human peripheral retina	Human Skeletal muscle	Mouse brain	Mouse spleen
**Comparion**	Hutchinson-Gilford progeria syndrome vs. healthy age-matched control	Alzheimer's disease (incipient) vs. healthy control	Alzheimer's disease (moderate) vs. healthy control	Alzheimer's disease (severe) vs. healthy control	Old vs. young	Old vs. young	Old sepsis induced multiple organ failure vs. old control	Old mouse inject LPS vs. adult mouse inject LPS	Old mouse vs. young mouse
RAD9A	2.46								
RAD1									
HUS1									
MRE11A								3.01	
RAD50							2.1		
NBN									
RPA1									
RPA2		2.15	2.09						
RPA3									
ATRIP					3.77				
RAD17									
PARP1	2.2								
PARP2									
XRCC6									
XRCC5			2.92						
IL1A									
BRCA1	3.43								3.56
TOPBP1									
TP53BP1									
MDC1	2.57								
H2AFX						7.23	2.14		2.45
ATM									
ATR				0.26			5.13		
PRKDC	6.45								
CHEK1	2.51								3.4
CHEK2	3.07								2.56
**Up**	7/26	1/26	2/26	0	1/26	1/26	3/26	1/26	4/26
**Down**	0	0	0	1/26	0	0	0	0	0

- Genes with significant expression changes ($P < 0.05$) are shown here. Red text stands for upregulation ($FC \geq 2$), green text stands for downregulation ($FC \leq 0.5$). FC: fold change.

Supplementary table 16. The gene expression changes of DNA damage repair factors are analyzed in aging and ageing diseases. Human Hutchinson-Gilford progeria syndrome, old sepsis induced multiple organ failure, and the spleen of old mouse induced upregulation of a few DNA damage repair factors.

**GEO ID**	GSE41751	GSE1297	GSE1297	GSE1297	GSE32614	GSE32614	GSE13205	GSE3253	GSE34378
**Tissue**	Human primary fibroblasts from skin	Human hippocampe	Human hippocampe	Human hippocampus	Human macular retina	Human peripheral retina	Human Skeletal muscle	Mouse brain	Mouse spleen
**Comparion**	Hutchinson-Gilford progeria syndrome vs. healthy age-matched control	Alzheimer's disease (incipient) vs. healthy control	Alzheimer's disease (moderate) vs. healthy control	Alzheimer's disease (severe) vs. Healthy control	Old vs. young	Old vs. young	Old sepsis induced multiple organ failure vs. old control	Old mouse inject LPS vs. adult mouse inject LPS	Old mouse vs. young mouse
MGMT									
ALKBH2									
ALKBH3									
OGG1									
NEIL1									
MUTYH									
UNG									
SMUG1									
MPG									
APEX1									
APEX2							6.59	6.63	
POLB									
PNKP									
XPC									
RAD23B	0.38								
XPA									
ERCC2									
ERCC3									
ERCC1									
ERCC5									
ERCC4									
LIG1	4.96								2.85
ERCC8									
ERCC6									
XRCC2	3.41								
XRCC3									
BRCA2			0.40	2.39		0.1			2.96
RAD52		0.39							
RAD51	2.23								2.87
LIG4									
NHEJ1									
XRCC4							3		
XRCC1									
LIG3	2.17						2.5		
MLH1									
MLH3									
MSH2	2.48								
MSH3					2.51				
MSH6									
PMS1									
PMS2									
HMGB1									
**Up**	5/42	0	0	1/42	1/42	0	3/42	1/42	3/42
**Down**	1/42	1/42	1/42	0	0	1/42	0	0	0

- Genes with significant expression changes ($P < 0.05$) are shown here. Red text stands for upregulation ($FC \geq 2$), green text stands for downregulation ($FC \leq 0.5$). FC: fold change

Supplementary table 17. The gene expression changes of DNA damage checkpoint factors are focused in human cancers. 1) Human colorectal carcinoma, esophageal squamous cell carcinoma, intrahepatic cancer, ovarian carcinomas, clear cell renal cell carcinomas and Burkitt lymphoma induced upregulation of a few DNA damage checkpoint factors; 2) Adult male germ cell tumors have all downregulation of DNA damage checkpoint factors; and 3) Pancreatic tumors and prostate cancer have no expression changes in DNA damage checkpoint factors

	**Digestive system**					**Reproductive system**				**Respiratory system**	**Urinary system**	
GEO ID	GSE 77955	GSE 79973	GSE 62452	GSE 45670	GSE 45001	GSE7 0951	GSE 46602	GSE 36668	GSE 3218	GSE 75037	GSE 71963	GSE 9327
Cancer type	Human Colorectal carcinoma	Human Gastric cancer	Human Pancreatic tumors	Human Esophageal squamous cell carcinoma	Human Intrahepatic cancer	Human Breast cancer	Human Prostate cancer	Human Ovarian carcinomas	Human Adult male germ cell tumors	Human Non-small cell lung cancers	Human Clear cell renal cell carcinomas	Human Burkitt lymphoma
RAD9A						0.22		2.13		2.31	2.03	
RAD1									0.46			
HUS1												2.03
MRE11A	2.15	0.41										
RAD50	3.11					0.33			0.37			
NBN	2.24										2.02	
RPA1												
RPA2												
RPA3												
ATRIP				0.43				2.28				
RAD17									0.16			2.22
PARP1						0.40		2.73		2.06		
PARP2												
XRCC6												
XRCC5	2.05											2.78
IL1A		3.05		3.37					0.40	0.29		
BRCA1				3.27				2.73		2.35	2.04	3.55
TOPBP1	3.02							2.10	0.45			
TP53BP1		2.22		2.64								
MDC1						2.13			0.26			
H2AFX				2.18	2.01			2.55		2.36		
ATM												
ATR	5.59			2.12				2.04	0.38	0.28		
PRKDC				2.12	2.57			2.35				2.18
CHEK1		2.37		3.52	9.98			4.86		3.76	2.50	
CHEK2				2.17								
**Up**	6/26	3/26	0	8/26	3/26	1/26	0	9/26	0	5/26	4/26	5/26
**Down**	0	1/26	0	1/26	0	3/26	0	0	7/26	2/26	0	0

- Genes with significant expression changes (P < 0.05) are shown here. Red text stands for upregulation (FC ≥2), green text stands for downregulation (FC ≤ 0.5). FC: fold change.

Supplementary table 18. The gene expression changes of DNA damage repair factors are focused in human tumors. Human esophageal squamous cell carcinoma, intrahepatic cancer, ovarian carcinomas, clear cell renal cell carcinomas and Burkitt lymphoma induced upregulation of a few DNA damage repair factors.

	**Digestive system**					**Reproductive system**				**Respiratory system**	**Urinary system**	
GEO ID	GSE77955	GSE79973	GSE62452	GSE45670	GSE45001	GSE70951	GSE46602	GSE36668	GSE3218	GSE75037	GSE71963	GSE9327
Cancer type	Colorectal carcinoma	Gastric cancer	Pancreatic tumors	Esophageal squamous cell carcinoma	Intrahepatic cancer	Breast cancer	Prostate cancer	Ovarian carcinomas	Adult male germ cell tumors	Non-small cell lung cancers	Clear cell renal cell carcinomas	Burkitt lymphoma
MGMT												
ALKBH2												
ALKBH3						4.69						
OGG1								0.44		0.45	0.44	2.30
NEIL1												
MUTYH												
UNG												
SMUG1								2.16				
MPG												
APEX1									3.30			2.08
APEX2				2.18								
POLB												
PNKP												
XPC						2.25						
RAD23B												
XPA												
ERCC2												
ERCC3									0.48			
ERCC1										0.32		
ERCC5												
ERCC4		0.48				5.35		2.93				2.16
LIG1											2.17	
ERCC8						0.37		2.19				
ERCC6												
XRCC2										4.14	3.05	
XRCC3										2.83		0.21
BRCA2				2.93		0.39		6.11			2.78	
RAD52												
RAD51		2.03		2.58	2.43			4.08		2.20	3.35	
LIG4												
NHEJ1												
XRCC4		2.25										
XRCC1												
LIG3					2.36							
MLH1									0.46			
MLH3												3.63
MSH2					2.17	0.23		2.13				2.16
MSH3												
MSH6												
PMS1												
PMS2						0.34						2.14
HMGB1	3.67					0.20						
**Up**	1/42	2/42	0	3/42	3/42	3/42	0	6/42	1/42	3/42	4/42	6/42
**Down**	0	1/42	0	0	0	5/42	0	1/42	2/42	2/42	1/42	1/42

- Genes with significant expression changes ($P < 0.05$) are shown here. Red text stands for upregulation ($FC \geq 2$), green text stands for downregulation ($FC \leq 0.5$). FC: fold change.

Supplementary table 19. The gene expression changes of DNA damage checkpoint factors in cells treated with LPS, MRP8 and some inflammatory cytokines

GEO ID	GSE36287				GSE68942				GSE56681				GSE6257	GSE37624
Cell	Human keratinocytes				Human renal tubular epithelial cells				Human blood cell				Human lymphatic endothelial cells	Human umbilical vein endothelial cells
Treatment	IL-17A	TNF-a	IFN-r	IL-4	IFN-r	IFN-r+ IL6	IFN-r+ TNF-a	IFN-r+ IL6+ TNF-a	LPS	MRP8	TNF-a	IL-1b	TNF-a	IL-33
RAD9A														
RAD1														
HUS1														
MRE11A									0.43	0.27				
RAD50														
NBN		0.31	6.26	0.32				2.41	9.12	4.69	3.6	2.01	3.3	
RPA1														
RPA2														
RPA3														
ATRIP		0.47												
RAD17										0.49				
PARP1									0.48	0.44				
PARP2														
XRCC6														
XRCC5		0.49												
IL1A	2.05	5.64							38.58	124.85	48.75	64.45		6.96
BRCA1										0.49				
TOPBP1														
TP53BP1														
MDC1			0.44											
H2AFX										0.49				
ATM														
ATR														
PRKDC			0.46			0.26	2.30	2.19						
CHEK1			0.41											
CHEK2			0.49				5.57						3.65	
**Up**	1/26	1/26	1/26	0	0	0	2/26	2/26	2/26	2/26	2/26	2/26	2/26	1/26
**Down**	0	3/26	4/26	1/26	0	1/26	0	0	2/26	5/26	0	0	0	0

- Genes with significant expression changes (P < 0.05) are shown here. Red text stands for upregulation (FC ≥2), green text stands for downregulation (FC ≤ 0.5).

Supplementary table 20. The gene expression changes of DNA damage repair factors in cells treated with LPS, MRP8 and some inflammatory cytokines.

GEO ID	GSE36287				GSE68942				GSE56681				GSE6257	GSE37624
Cell	Human keratinocytes				Human renal tubular epithelial cells				Human blood cell				Human lymphatic endothelial cells	Human umbilical vein endothelial cells
Treatment	IL-17A	TNF-a	IFN-r	IL-4	IFN-r	IFN-r+ IL6	IFN-r+ TNF-a	IFN-r+ IL6+ TNF-a	LPS	MRP8	TNF-a	IL1b	TNF-a	IL-33
MGMT														
ALKBH2														
ALKBH3														
OGG1		0.44		0.41						0.25	0.5			
NEIL1					2.03									
MUTYH		0.49												
UNG														
SMUG1														
MPG					3.63	4.82		4.35						
APEX1		0.38												
APEX2										0.49				
POLB														
PNKP														
XPC														
RAD23B														
XPA		0.45							0.46	0.29				
ERCC2														
ERCC3														
ERCC1										0.4				
ERCC5														
ERCC4					0.34					0.37	0.42	0.49		
LIG1		0.37												
ERCC8		0.34		0.45										
ERCC6														
XRCC2						0.47								
XRCC3														
BRCA2							3.92						3.88	
RAD52							2.38	2.14						
RAD51			0.28											
LIG4							4.89							
NHEJ1														
XRCC4										0.34			2.88	
XRCC1									0.49	0.49				
LIG3							2.95							
MLH1		0.35												
MLH3							3.61							
MSH2						0.2		2.17	0.41	0.21	0.32			
MSH3		0.47			3.1	3.39				0.44				
MSH6			0.4						0.42	0.42	0.47			
PMS1														
PMS2							0.4	0.23						
HMGB1		0.48				0.21			0.36	0.4	0.46			
**Up**	0	0	0	0	3/42	2/42	5/42	3/42	0	0	0	0	2/42	0
**Down**	0	9/42	2/42	2/42	1/42	3/42	1/41	1/42	5/42	11/42	5/42	1/42	0	0

- Genes with significant expression changes ($P < 0.05$) are shown here. Red text stands for upregulation ($FC \geq 2$), green text stands for downregulation ($FC \leq 0.5$).

Supplementary table 21. The gene expression changes of DNA damage checkpoint factors in tissues where oxidative stress associated genes deleted.

**GEO ID**	GSE39629	GSE8969	GSE35124	GSE6623	GSE8726	GSE92530	GSE52550
**Tissue**	Mouse Esophageal epithelium	Mouse liver	Mouse liver	Mouse bone marrow hematopoietic stem cells	Mouse Erythroblasts	Mouse blood	Mouse muscle
**Comparison**	Nrf2-/- vs. Wild type	Nrf2 -/- vs. Wild type	Keap1 -/- vs. Wild type	FoxO1/3/4-/- vs. Wild type	Sod2-/- vs. Sod2+/+ mice	wild type mice during collagen induce arthritis vs. Ncf1-/- induce arthritis	Old muscle-specific PGC-1alpha knock-out mice vs. wild-type
RAD9A							
RAD1							
HUS1							
MRE11A							
RAD50							
NBN							
RPA1							
RPA2							
RPA3							
ATRIP							
RAD17							
PARP1							
PARP2							
XRCC6							
XRCC5							
IL1A	4.56			12.73			
BRCA1				0.11			
TOPBP1							
TP53BP1							
MDC1							
H2AFX							
ATM							
ATR				2.68			
PRKDC							
CHEK1				5.10			
CHEK2							
**Up**	1/26	0	0	3/26	0	0	0
**Down**	0	0	0	1/26	0	0	0

- Genes with significant expression changes ($P < 0.05$) are shown here. Red text stands for upregulation ($FC \geq 2$), green text stands for downregulation ($FC \leq 0.5$).

Supplementary table 22. The gene expression changes of DNA damage repair factors are focused where oxidative stress associated genes were deleted .

**GEO ID**	GSE39629	GSE8969	GSE35124	GSE6623	GSE8726	GSE92530	GSE52550
**Tissue**	Mouse Esophageal epithelium	Mouse liver	Mouse liver	Mouse bone marrow hematopoietic stem cells	Mouse Erythroblasts	Mouse blood	Mouse muscle
**Comparison**	Nrf2-/- vs. Wild type	Nrf2 -/- vs. Wild type	Keap1 -/- vs. Wild type	FoxO1/3/4-/- vs. Wild type	Sod2-/- vs. Sod2+/+	wild type mice during collagen induce arthritis vs. Ncf1-/- induce arthritis	Old muscle-specific PGC-1alpha knock-out mice vs. wild-type
MGMT							
ALKBH2							
ALKBH3							
OGG1							
NEIL1							
MUTYH							
UNG							
SMUG1							
MPG							
APEX1							
APEX2							
POLB							
PNKP							
XPC							
RAD23B			0.30				
XPA		0.26					
ERCC2							
ERCC3							
ERCC1							
ERCC5		0.48					
ERCC4							
LIG1							
ERCC8			0.45				
ERCC6							
XRCC2							
XRCC3							
BRCA2							
RAD52							
RAD51							
LIG4							
NHEJ1							
XRCC4							
XRCC1							
LIG3							
MLH1							
MLH3							
MSH2	0.48	2.62					
MSH3							
MSH6					2.27		
PMS1							
PMS2							
HMGB1							
**Up**	0	1/42	0	0	1/42	0	0
**Down**	1/42	2/42	2/42	0	0	0	0

- Genes with significant expression changes ($P < 0.05$) are shown here. Red text stands for upregulation ($FC \geq 2$), green text stands for downregulation ($FC \leq 0.5$).
